# Using *Drosophila* to identify naturally occurring genetic modifiers of amyloid beta 42- and tau-induced toxicity

**DOI:** 10.1093/g3journal/jkad132

**Published:** 2023-06-13

**Authors:** Ming Yang, Matthew Zinkgraf, Cecilia Fitzgerald-Cook, Benjamin R Harrison, Alexandra Putzier, Daniel E L Promislow, Adrienne M Wang

**Affiliations:** Department of Laboratory Medicine and Pathology, University of Washington School of Medicine, Seattle, WA 98195, USA; Department of Biology, Western Washington University, Bellingham, WA 98225, USA; Department of Laboratory Medicine and Pathology, University of Washington School of Medicine, Seattle, WA 98195, USA; Department of Laboratory Medicine and Pathology, University of Washington School of Medicine, Seattle, WA 98195, USA; Department of Biology, Western Washington University, Bellingham, WA 98225, USA; Department of Laboratory Medicine and Pathology, University of Washington School of Medicine, Seattle, WA 98195, USA; Department of Biology, University of Washington, Seattle, WA 98195, USA; Department of Biology, Western Washington University, Bellingham, WA 98225, USA

**Keywords:** *Drosophila*, neurodegeneration, DGRP, Alzheimer's disease, genome-wide association, multiple traits

## Abstract

Alzheimer's disease is characterized by 2 pathological proteins, amyloid beta 42 and tau. The majority of Alzheimer's disease cases in the population are sporadic and late-onset Alzheimer's disease, which exhibits high levels of heritability. While several genetic risk factors for late-onset Alzheimer's disease have been identified and replicated in independent studies, including the ApoE ε4 allele, the great majority of the heritability of late-onset Alzheimer's disease remains unexplained, likely due to the aggregate effects of a very large number of genes with small effect size, as well as to biases in sample collection and statistical approaches. Here, we present an unbiased forward genetic screen in *Drosophila* looking for naturally occurring modifiers of amyloid beta 42- and tau-induced ommatidial degeneration. Our results identify 14 significant SNPs, which map to 12 potential genes in 8 unique genomic regions. Our hits that are significant after genome-wide correction identify genes involved in neuronal development, signal transduction, and organismal development. Looking more broadly at suggestive hits (*P* < 10^−5^), we see significant enrichment in genes associated with neurogenesis, development, and growth as well as significant enrichment in genes whose orthologs have been identified as significantly or suggestively associated with Alzheimer's disease in human GWAS studies. These latter genes include ones whose orthologs are in close proximity to regions in the human genome that are associated with Alzheimer's disease, but where a causal gene has not been identified. Together, our results illustrate the potential for complementary and convergent evidence provided through multitrait GWAS in *Drosophila* to supplement and inform human studies, helping to identify the remaining heritability and novel modifiers of complex diseases.

## Introduction

Alzheimer's disease (AD) currently affects 6.2 million individuals over the age of 65 in the United States, with the US AD patient population projected to increase to 12.7 million by 2050 (2021 AD facts and figures 2021). Although familial AD, or early-onset AD (EOAD), is caused by rare and highly penetrant mutations in 1 of 3 genes [amyloid precursor protein (APP), presenilin 1 (PSEN 1), and presenilin 2 (PSEN 2)], a majority (>90%) of AD cases are sporadic and classified as late-onset AD (LOAD). In both forms of the disease, the abnormal processing of 2 proteins, APP and tau, are defining features, leading to the characteristic accumulation of extracellular amyloid beta plaques containing the peptide amyloid beta 42 (Aβ42) cleaved from APP and intracellular neurofibrillary tangles composed of hyperphosphorylated tau. While LOAD is non-Mendelian in nature, there is a significant genetic predisposition for the disease, with heritability estimated to be as high as 80% (Gatz *et al*. 2006). Thus far, 20 genetic risk factors have been identified for LOAD through international genome wide association studies (GWAS), the strongest of which is the apolipoprotein E (ApoE) ε4 allele (Harold *et al*. 2009; Seshadri *et al*. 2010; Hollingworth *et al*. 2011; Naj *et al*. 2011; Lambert *et al*. 2013; Kunkle *et al*. 2019). These 20 genes (including ApoE ε4) account for less than one third of the total genetic variance (Gatz *et al*. 2006; Ridge *et al*. 2016). The remaining heritability is likely due to contributions made by many genes with lesser impact, by very rare alleles, and/or by genes acting epistatically, all of which prove difficult to find through GWAS in highly heterogeneous human populations. Identification of true risk factors is complicated by analysis that relies on genome-wide testing of multiple hypotheses, increasing the likelihood of false positives. Thus far, researchers have typically corrected for multiple comparisons by applying a stringent threshold (*P* < 10^−8^) to determine significance, which, while cutting down on false positives, also makes it likely that there are many true modifiers that simply do not reach this threshold. While these canonical approaches focus on univariate analysis, linking single phenotypes with single SNPs or genes, segregating SNPs do not necessarily map to the causal gene, but rather implicate a region that may be associated with numerous genes or transcriptional control elements. More recently, multivariate and multitrait GWAS approaches have emerged that leverage multiple correlated traits to boost signal and increase sensitivity, allowing for detection of variants that have otherwise been missed by univariant screening (Julienne *et al*. 2021).

Improved approaches to identify true genetic modifiers of LOAD will not only help identify potential therapeutic targets, but will also shed light on the highly dimensional and polygenic processes that underlie disease progression, helping to identify at-risk populations that might benefit from early therapeutic intervention. The fruit fly *Drosophila melanogaster* has emerged as a powerful model organism in lab-based efforts to study neurodegeneration and to identify genes associated with human disease. Flies exhibit many conserved cellular processes, including those implicated in human LOAD, such as immune response, inflammation, and lipid metabolism, and nearly 70% of human disease-causing genes have orthologs in *Drosophila* (Chintapalli *et al*. 2007; Yamamoto *et al*. 2014). Neuronal expression of human Aβ42 in flies leads to decreased lifespan, progressive neurodegeneration, motor deficits, and accumulation of aggregates (Finelli *et al*. 2004; Iijima *et al*. 2004). Expression of Aβ42 in the neuronal and support cells of the *Drosophila* compound eye results in disorganization of the ommatidial array of the eye that is easily visible and quantifiable as a “rough eye” phenotype (Finelli *et al*. 2004; Iijima *et al*. 2004; Crowther *et al*. 2005; Fernandez-Funez *et al*. 2015).

Although much of what we have learned about AD progression has come from studies focused on the abnormal processing and accumulation of Aβ, the phosphorylation of tau has also been shown to be a key player in disease pathogenesis, and mounting evidence suggests that there may be a synergistic interaction between Aβ42 and tau (reviewed in Busche and Hyman 2020). Expression of human tau in the fly leads to hyperphosphorylation of tau and results in phenotypes similar to those seen with Aβ42 expression, including decreased lifespan, neurodegeneration, and ommatidial degeneration (Wittmann *et al*. 2001; Khurana 2008). While EOAD represents a very small proportion of the AD patient population, the majority of animal models of AD have focused on monogenic mutations associated with EOAD (mutations in APP, PSEN 1, and PSEN 2) to model AD (Drummond and Wisniewski 2017). Many of these animal models recapitulate key aspects of the disease, but all have failed to fully encompass the entire spectrum of human AD pathology (Claussnitzer *et al*. 2020; Qin *et al*. 2020). Further impacting translation of findings, these monogenic models are commonly studied in single genetic backgrounds and so do not account for the fact that disease pathogenesis occurs in a unique genetic background in each human being, often with a large number of SNPs that could modify disease progression.

To create an animal model of AD that more accurately reflects disease progression across different genetic backgrounds, we combined 2 previously published fly models that model the downstream pathology in both EOAD and LOAD—Aβ42 accumulation and hyperphosphorylation of tau [Supplementary Fig. 1 (Wittmann *et al*. 2001; Finelli *et al*. 2004)]—with a model of natural genetic variation, the *Drosophila* Genetic Reference Panel (DGRP) (Mackay *et al*. 2012). The DGRP is a library of more than 200 inbred and fully sequenced *Drosophila* lines derived from a wild-caught population, providing a powerful genetic tool with which to study the effects of natural variation on complex traits. Studies using the DGRP have successfully identified susceptibility loci for a number of disease-relevant traits such as protein folding, neurodegeneration, longevity, and stress resistance (Weber *et al*. 2012; He *et al*. 2014; Ivanov *et al*. 2015; Najarro *et al*. 2015; Chow *et al*. 2016; Zhou *et al*. 2016; Lavoy *et al*. 2018; Harrison *et al*. 2020). By expressing both Aβ42 and tau across the DGRP, measuring multiple metrics underlying the rough eye phenotype, and performing GWAS across multiple, correlated traits, our goal was to leverage the statistical power, environmental control, and repeated measures afforded by *Drosophila* to identify naturally occurring genetic modifiers of Aβ42- and tau-induced toxicity.

While human-based, qualitative scores of the rough eye phenotype have been successfully used to identify strong modifiers in multiple *Drosophila* models of neurodegenerative diseases, efforts to identify weaker modifiers of disease have been constrained by sensitivity of phenotypic classification (Lessing and Bonini 2009; Iijima-Ando and Iijima 2010; Fernandez-Funez *et al*. 2015). The emergent property of “roughness” is caused by changes to the structure of individual ommatidia such as increased pitting and fusion, as well as by changes to the relative spacing of individual ommatidia. Each of these disrupt regularity in the normally highly ordered array where individual ommatidia are of uniform size, circularity and roundness, with each centered a uniform distance from 6 nearest neighboring ommatidia (Tomlinson *et al*. 1988; Basler *et al*. 1991). Degenerative phenotypes are therefore associated with increased variation in distance from nearest neighboring ommatidia, as well as with fused and pitted ommatidia that have a larger area, a larger perimeter, and a loss of circularity (Finelli *et al*. 2004; Crowther *et al*. 2005; Fernandez-Funez *et al*. 2015). Manual assessment of the rough eye phenotype that emerges from these geometric features is usually performed by blinded individuals who qualitatively assess the severity of eye degeneration based on ommatidial disorganization (severity, area of eye affected), loss of pigment, or changes in eye size. In conjunction with the large number of the vital genes in flies that are involved in eye development, this approach has made it possible for the rough eye phenotype to be used to successfully screen for and identify strong enhancers and suppressors of multiple neurodegenerative diseases (Fernandez-Funez *et al*. 2000; Shulman and Feany 2003; Yang *et al*. 2005; VoSSfeldt *et al*. 2012; Chow *et al*. 2016). However, manual and qualitative assessment of the rough eye phenotype may lack the sensitivity to differentiate the more subtle and continuously distributed effects that result from weak modifiers of complex traits. Quantitative measurement of multiple features that contribute to the *Drosophila* rough eye phenotype could allow for detection of the phenotypic variation that is critical for identifying genetic variants with small effect size.

Automated image analysis allows for fast, sensitive and discrete quantification of multiple features of the rough eye phenotype based on changes to the geometry of individual ommatidia. It provides a multifaceted, reproducible, and sensitive readout of ommatidial integrity. Recent efforts to automate quantitative assessment of the rough eye phenotype have enabled researchers to measure attributes of the rough eye with increased sensitivity and reliability beyond that which can be quantified by the human eye. Programs such as Flyeye (Diez-Hermano *et al*. 2015) and Flynotyper (Iyer *et al*. 2016) use geometric features extracted from ommatidial images but require manual identification of the region of interest (ROI) for analysis or focus on only one feature, such as the spatial distribution of ommatidia, to extract a score of “orderliness.” An even more recent approach makes use of machine learning techniques and an image classification algorithm to assign input images into categorical classes. However, this requires significant pretraining steps and results can be difficult to interpret (Diez-Hermano *et al*. 2020).

To quickly and efficiently leverage ommatidial geometry from multiple levels, we developed an automated and interpretable image analysis pipeline that detects individual ommatidia and extracts multiple geometrical properties within and between ommatidia. This pipeline analyzes images of fly eyes to quantify 16 different metrics associated with the geometric organization of the ommatidium, including the area, radius, and perimeter of individual ommatidia, as well as inter-ommatidial distance to the nearest neighboring ommatidium. We then used the 14 metrics that were both heritable and correlated with human scoring to map associated SNPs across multiple traits. We observe significant natural variation across the DGRP in each of the traits analyzed, and GWA analyses across all traits identified numerous SNPs significantly associated with individual traits, as well as suggestive genes associated with numerous traits. This list of putative modifiers is significantly and specifically enriched for genes involved in transmembrane transport, cellular adhesion and growth, as well as neuronal morphogenesis, and we identify a number of genes that have previously been functionally implicated in response to both Aβ and tau, highlighting potential mechanisms by which Aβ42 and tau interact. Furthermore, our list of candidate modifiers is also significantly enriched for genes that have been implicated in human GWA studies of AD, providing validation for an approach by which to interrogate and validate true modifiers that trend toward, but fail to reach, genome-wide significance across numerous human GWAS. Together, these results highlight the potential of an approach that makes use of multiple quantitative measures, and allows us to combine the strengths of forward genetic screens in model organisms with the power of human GWAS to identify novel risk factors with small effect size.

## Materials and methods

### *Drosophila* stocks and culture

All flies were reared on standard cornmeal-agar-sugar-yeast CT food at 25°C with 50–60% relative humidity under a 12-hr light-dark cycle. The 162 DGRP lines used in this study were obtained from the Bloomington *Drosophila* Stock Center (BDSC, Bloomington, Indiana). The Aβ42- and tau-expressing “R32” line was created by recombining the AB42 transgene under control of the upstream activation sequence (UAS) UAS-Aβ42 (Finelli *et al*. 2004) with the glass multimer reporter Gal4 driver (GMR-Gal4) (BDSC #1104, Bloomington, Indiana) on chromosome II and then crossing those flies with flies carrying UAS-tau(0N4R) on chromosome III (Wittmann *et al*. 2001). All crosses were performed after transgenic lines were backcrossed for 6 generations to the *w^1118^* background (BDSC #5905, Bloomington, Indiana) and the transgenes were maintained over the Cyo and TM6, Tb balancers. The genotype of the resulting triple transgenic R32 line was confirmed by PCR, and expressions of Aβ42 and tau were confirmed by Western blot (Supplementary Fig. 1). A single isogenized line was used as our triple transgenic donor line for initial analyses, and a second isogenized line derived from the first was used for later experiments.

### Phenotypic analysis

#### Automated quantification of ommatidial degeneration

**Fig. 1.**
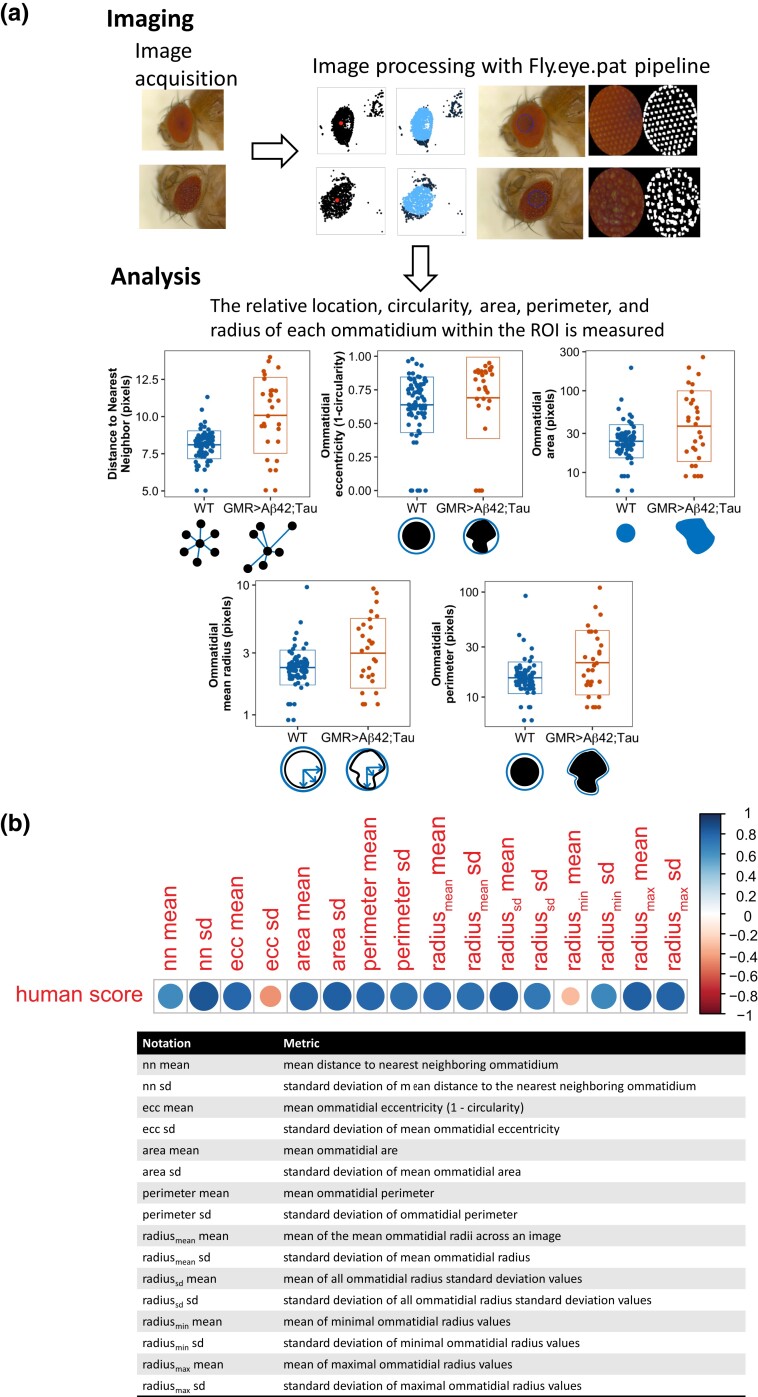
Expression of human Aβ42 and tau in the *Drosophila* eye results in quantifiable degeneration. a) Automated image analysis pipeline. Images are acquired, processed to identify individual ommatidium within an ROI, and 5 ommatidial measurements are extracted including ommatidial radius, perimeter, and circularity, as well as relative location. From these measurements, 14 features are calculated, including distance to the nearest neighbor, minimal and maximal radii, mean values, and standard deviation within an image and across replicates. Central measurements (nn mean, ecc mean, area mean, radius_mean_ mean, and perimeter mean) from a WT fly and a GMR > Aβ42; tau expressing fly from our triple-transgenic donor line in the *w1118* background is shown in the bottom panels. b) Heat map showing degree of correlation between the BLUP of machine-generated scores and human-generated scores (top, *P* < 0.05 for all traits except radius_min_ mean). Size and color of each trait based on the Spearman's correlation coefficient (*R*). Bottom, table with metric notation and descriptions.

Our automated image analysis pipeline (Fig. 1a) measured ommatidial degeneration in fly eye images using the R programming language. This pipeline consists of 2 main steps: (1) automated ROI selection and (2) image feature quantification.

1. Automated ROI selection: Colored images were converted to grayscale using the top-hat morphological transform function from the R package EBImage (Pau *et al*. 2010). Our program then selected the ROI using an algorithm developed in Diez-Hermano *et al*. 2020 (Diez-Hermano *et al*. 2020). Briefly, this algorithm detected high-intensity pixels in an image to locate the centroid. The distance between every pixel and the centroid was calculated, and pixels with distances larger than 0.8 quantile were discarded. A confidence level ellipse drawn on the remaining pixels was extracted as the final ROI.
2. Image feature quantification: Within the ROI, individual ommatidia were identified and marked as a pixel cluster using image segmentation techniques. Basic measurements such as position coordinates, area, perimeter, radius, including mean radius (radius_mean_), minimum radius (radius_min_), maximal radius (radius_max_), and the standard deviation (SD) of the radius (radius_SD_) were computed for each identified ommatidium using functions from EBImage (Pau *et al*. 2010).

Based on these basic ommatidial metrics, we then calculated the arithmetic mean and SD for each metric across all ommatidia detected within an ROI of an image. This resulted in 16 trait metrics for each image: mean distance to nearest-neighboring ommatidium (nn mean), SD of mean distance to the nearest-neighboring ommatidium (nn SD), mean ommatidial eccentricity (ecc mean), standard deviation of mean ommatidial eccentricity (ecc SD), mean ommatidial area (area mean), standard deviation of mean ommatidial area (area SD), mean ommatidial perimeter (perimeter mean), standard deviation of ommatidial perimeter (perimeter SD), mean of the mean ommatidial radii across an image (radius_mean_ mean), standard deviation of mean ommatidial radius (radius_mean_ SD), mean of all ommatidial radius standard deviation values (radius_SD_ mean), standard deviation of all ommatidial radius standard deviation values (radius_SD_ SD), mean of minimal ommatidial radius values (radius_min_ mean), standard deviation of minimal ommatidial radius values (radius_min_ SD), mean of maximal ommatidial radius values (radius_max_ mean), and standard deviation of maximal ommatidial radius values (radius_max_ SD).

**Fig. 2.**
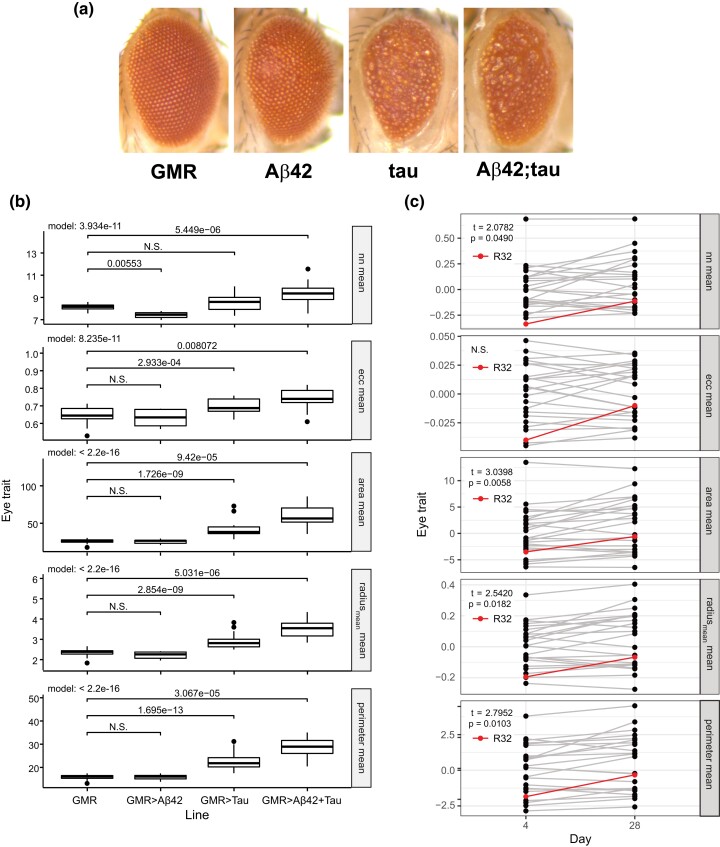
Age and Aβ42 expression exacerbate tau-induced degeneration. a) Representative images (upper panels) from 28-day-old flies including a phenotypically WT control fly carrying only the GMR-Gal4 driver (GMR), a fly expressing a single copy of human Aβ42 driven by the GMR driver (Aβ42), a fly expressing a single copy of the 0N4R isoform of human tau driven by the GMR driver (tau), and our stably expressing fly line expressing both human Aβ42 and tau driven by the GMR driver (Aβ42; tau). b) Quantification of central ommatidial traits is shown (nn mean, ecc mean, area mean, radius_mean_ mean, perimeter mean). Data presented are the relative effect of genotype compared to the GMR control based on a fixed-effect model. c) Degree of ommatidial degeneration in flies expressing GMR > Aβ42; tau in 23 DGRP backgrounds and in our triple transgenic donor stock in the *w^1118^* background (R32, red line) on day 4 (left) and day 28 (right). Each plot shows the BLUP for each line across 5 central traits (nn mean, ecc mean, area mean, radius_mean_ mean, perimeter mean) over age. Age exacerbates degeneration of the R32 line in all 5 central traits and 4 central traits show significant worsening with age on average (paired sample *t*-test, with *t*-test statistic and *P*-value shown).

Our automated fly eye image processing pipeline represents a novel approach to quantify the *Drosophila* rough eye phenotype. Unlike previous studies which used the total fly eye size or area (He *et al*. 2014; Chow *et al*. 2016), our pipeline starts with an automated ROI selection procedure and then evaluates fly eye degeneration based on ommatidium-level metrics. Although expression of both Aβ42 and tau lead to significant morphological defects in the fly eye, there is no obvious reduction in overall eye size, volume, or area. In contrast, we observed overt changes in geometric properties of individual ommatidia, and we were able to detect effects across ommatidia within a single image (Figs. 1a, 2a and b and Supplementary Fig. 4). In addition, the *Drosophila* eye is a 3D organ by nature, while eye imaging necessitates the capture of a 2D image. This makes the measurement of eye area and volume substantially dependent on the position of the fly eye under the camera. Our ROI selection algorithm identifies areas of the rounded ommatidium that are within the focal plane, minimizing the confounding effect of fly eye positioning during the imaging process. Within each ROI, our algorithm characterizes not only ommatidial organization across the eye through analysis of the variance of distance from one ommatidium to its nearest neighbors, but also the variation across geometric properties of individual ommatidium such as ommatidial area, perimeter, and circularity.

Through manual inspection, we found that most eye images in our study showed pitting, fusion, and collapse of ommatidia (Supplementary Fig. 5), which were captured by the ommatidial area, ommatidial perimeter, and ommatidial radius-related metrics (Fig. 1b). The average values of these metrics were in general larger in defective eyes than in wild-type (WT) eyes, which is consistent with the detection of fused and pitted ommatidia—ommatidia that would then have a larger area, a larger perimeter, less circularity, and less uniform radii. The exception to this was the metric of ommatidial minimal radius, whose values were smaller in the defective eyes (Fig. 1a and Supplementary Fig. 6). Furthermore, the nearest-neighbor distances between ommatidia can be utilized to depict their spatial organization (Iyer *et al*. 2016), in which case a defective eye with an irregular arrangement exhibits an increased mean and variance of the nearest neighbor distance distribution (Fig. 1a). Together, our pipeline implemented an automated approach to quantify the extent of fly eye degeneration with multiple metrics at a finer scale.

Validation of our pipeline was performed by comparing the best linear unbiased predictor (BLUP) of human scores with the BLUP of machine-generated scores for 200 images of ommatidial arrays with varying degrees of degeneration. We found significant correlation between the degree of degeneration in 15 of 16 traits assessed by our pipeline and human assessment (Fig. 1b, Spearman's *ρ* ranged from −0.444 to 0.860). This approach allowed us to measure both centrality and dispersion of 6 measurements (nn, radius, area, perimeter, circularity, distance to nearest neighbor) and resulted in 16 traits that together encompass the degree of degeneration of individual ommatidia and the ommatidial array as a whole, key elements that contribute to the rough-eye phenotype. We chose to focus on the 14 traits that showed significant heritability (*H*^2^ > 0.05, Supplementary Fig. 3) and that significantly correlated with human scoring. For ease of presentation, we further classified these 14 traits into what we consider “central” measurements (nn mean, ecc mean, area mean, radius_mean_ mean, and perimeter mean) and “dispersion” measurements (nn SD, ecc SD, area SD, radius_mean_ SD, perimeter SD, radius_max_ mean, radius_max_ SD, radius_min_ mean, and radius_min_ SD). Importantly, these measurements do not significantly correlate with other eye phenotypes caused by proteostatic disruption in the Drosophila eye, indicating that our measurements are specific to Aβ42- and tau-induced pathology (Supplementary Fig. 7).

#### Quantification of degeneration caused by Aβ42 and tau

To determine the extent to which simultaneous expression of Aβ42 and tau exacerbates the rough eye phenotype, we expressed Aβ42 and tau in the fly eye both independently, and together, using the GMR-Gal4 driver. Control flies expressing one copy of GMR-Gal4 in the absence of UAS-responsive elements were used as a WT control. Flies were aged out for 28 d and the eyes assessed for ommatidial degeneration (see *Automated quantification of ommatidial degeneration* above). To assess the influence of transgenes on eye degradation, we used a fixed-effect interaction model to quantify the individual additive effects of Aβ42 and tau and the nonadditive interaction between transgenes.

#### DGRP screening for ommatidial degeneration

Experimental F_1_-generation flies were produced by crossing unmated virgin females from 162 DGRP lines to males from the GMR > Aβ42;tau (R32) line. F_1_ progeny were collected 0–48 hr after eclosion under light CO_2_ anesthesia, allowed to mate for 48 hr and then separated by sex and genotype. Female flies carrying GMR-Gal4, UAS-Aβ42 and UAS-tau(0N4R) were transferred to vials with up to 20 flies per vial and aged out to 28d of age, transferring flies to fresh food every 2–3 d. On day 28, experimental flies were lightly anesthetized, and a single eye/fly was imaged at 50 × using a Stemi 508 stereomicroscope with an Axiocam 105 color camera (Zeiss) attached. Only lines that produced more than 10 good-quality images per line were used, resulting in images from 162 DGRP lines. Lines were run in groups containing between 8 and 49 lines. For each group, a heterozygous R32 line in the *w^1118^* background was used as a positive control, and flies resulting from crosses between females and males of the DGRP line 441 were used as a negative control (WT eye). Images acquired were fed into the analysis pipeline (see *Automated quantification of ommatidial degeneration*) to quantify the degree of ommatidial degeneration in flies expressing both Aβ42 and tau under control of the eye-specific GMR-Gal4 promoter across different genetic backgrounds.

#### Estimation of the best linear unbiased predictors and broad-sense heritability

The phenotypic variance at 28 d was assessed in a mixed linear model using the lme4 package implemented in R (Bates *et al*. 2015), using restricted maximum likelihood and specified as follows: Y*_ijk_* = *µ* + *G_i_* + *B _j_* + *L_k_* + ε*_ijk_* where *μ* is the general mean; *G* is the effect of image group *i*, considered as fixed; *B* is the effect of the batch *j*, considered as fixed; and *L* is the effect of the line *k*, considered as random. When comparing different lines over time, a similar model was applied to samples from a 4 d experiment but without the fixed effects associated with group and batch.

The variance component estimates resulting from this analysis were used to estimate the broad-sense heritability (*H^2^*) using the following equation: *H^2^* = $\sigma_{line}^{2}/\;\sigma_{total}^{2}$, where the variance of the lines ($\sigma_{line}^{2}$) represents the variance of the replicates of each line in our model, and variance total ($\sigma_{total}^{2}$) represents the sum of the total variance of the model. The 95% confidence interval around the point estimate *H^2^* was estimated using a bootstrap procedure (bootmer function from the lme4 package) with 1,000 simulations. Mixed linear models were also used to estimate the BLUPs of the effect of lines on each phenotypic trait across image groups and experimental batches. These BLUP estimates were used for the subsequent statistical and genomic analysis.

### Genome-wide association

#### Single-SNP association analysis

Genetic variants of DGRP lines and annotation were downloaded from the DGRP website (http://dgrp2.gnets.ncsu.edu/data.html). The raw data included approximately 4.4 million variants of 205 DGRP lines. Discarding genetic variants with missing rate >0.2 or minor allele frequency <0.05 in the 162 DGRP lines, approximately 1.87 million genetic variants were retained. The genes associated with genetic variants were determined using the variant annotation based FB5.57.

To account for potential confounding factors such as population structure, DGRP inversion status, and *Wolbachia* infection status, we first used PLINK (v1.90; Purcell *et al.* 2007) to perform a principal component (PC) analysis on all retained genetic variants of the 162 DGRP lines and found that the first 3 PCs could separate inversion status distribution among genotypes. We then further tested PC importance via their corresponding eigenvalue significance with a Tracy–Widom test in the R package AssocTests (Wang *et al.* 2020). The first 4 PCs were kept as important at an α = 0.05 significance level.

Finally, we used a linear additive model implemented in PLINK to perform a genome-wide association analysis on the BLUP estimates for each trait and included PC1 to PC4 and *Wolbachia* infection status as covariates. The raw *P* value for each genetic variant generated by PLINK was corrected for multiple testing with the p.adjust function in R, applying the Benjamini & Hochberg (BH) method (Benjamini and Hochberg 1995). Two sets of SNPs were identified from the genome wide association study (GWAS) analysis. First, SNPs that had a *P*.adjust < 0.05 were designated as having statistically significant associations with the focal trait. Second, suggestive associations were designated as SNPs that had a raw *P* value of < 1e−5, in line with previous DGRP studies (Mackay *et al*. 2012).

### Enrichment analyses

#### Gene ontology enrichment analysis

Using the genes associated with suggestive SNPs (*P* value < 1e-5), we performed gene ontology (GO) enrichment analysis with the R package clusterProfiler [v3.16.1 (Yu *et al*. 2012) and the genome annotation for Fly from Bioconductor (org.Dm.eg.db; v3.11.4)]. Since the GO database for *Drosophila* contains 1,000s of potential functions, this package controls for the effect of multiple testing using the Benjamini & Hochberg method (Benjamini and Hochberg 1995) to report the adjusted *P* value (p.adjust) and the number of enriched genes for each significant GO term (*P*.adjust < 0.05). The GO enrichment results were further characterized using the Wang method of semantic similarity (Wang *et al*. 2007) and clustered using binary cut as implemented in the R package simplifyEnrichment (v1.5.2) (Gu and Hübschmann 2022).

#### Enrichment of AD-associated human orthologs

Using the genes associated with suggestive SNPs (*P* < 10^−5^), we wanted to determine if the DGRP results were enriched for *Drosophila* genes that were orthologs to human genes previously associated with AD. Due to the complex orthologous relationships (ranging from one-to-one and many-to-many) between *Drosophila* and humans, we used a permutation test to determine enrichment. The permutation test was conducted by randomly sampling 207 fly genes from the *Drosophila* genome (FlyBase Release 6.32; https://flybase.org) and merging each random set with the DIOPT version 8 (score ≥ 3) (Hu *et al.* 2011) to identify human orthologs. For each iteration, the number of fly-human orthologous pairs that had previously been associated with AD traits was recorded. Associations between human genes and AD traits were based on the NHGRI-EBI GWAS catalog [https://www.ebi.ac.uk/gwas/, data retrieved May 3, 2020 (Buniello *et al.* 2019)]. The permutation test was run for 10^6^ iterations and the *P* value for enrichment was determined as the proportion of permutation runs for which the number of fly-human orthologs in the permuted data (*ho_perm_*) was greater than or equal to the observed (red dashed line) number of fly-human orthologs (*ho_rand_*) linked to human AD [*P* value = #(*ho_perm_* ≥ *ho_obs_*)/10^6^].

### Fluorescence quantification

We rank ordered the lines for each trait and identified 12 DGRP lines in which expression of Aβ42 and tau led to a “high” degree of degeneration (among the highest 1/3 of samples with a ranking of over 108 of 162 lines), and 12 lines in which expression of Aβ42 and tau led to a “lower” degree of degeneration (among the lowest 1/3 of samples with a ranking of less than 58 out of 162 lines). Virgin females from those DGRP lines were crossed to male flies homozygous for GMR-Gal4 and an UAS responsive red fluorescent protein (UAS-RFP) or to male flies homozygous for the UAS-RFP only, to produce experimental F_1_ flies expressing GMR > RFP in a heterozygous DGRP background and F_1_ control flies carrying UAS-RFP in a heterozygous DGRP background. Four to 5 biological replicates of 3 28-day-old mated female flies of each genotype were snap frozen in liquid nitrogen, homogenized in 50 µl 25 mM Tris, 150 mM NaCl, 1% Triton X-100, 0.1% SDS, 1X protease-inhibitors (Thermo-Fisher A32965), and centrifuged at 14k rcf for 5 min, in a dark room. Supernatants were loaded onto a 96-well plate and fluorescence was measured using a Biotek Synergy H1 plate reader with 580 nm excitation and 610–635 nm emission wavelengths. Arbitrary fluorescence units (AFUs) for the experimental flies were normalized to the mean AFU from the control (non-RFP-expressing) flies.

### RNA extraction and RT-qPCR

We extracted RNA from 3 biological replicates of 6–10 28-day-old flies carrying GMR > Aβ42; tau in the heterozygous DGRP background from 6 DGRP lines in which expression of Aβ42 and tau led to a “high” degree of degeneration (criterion described above), and 7 lines in which expression of Aβ42 and tau led to a “lower” degree of degeneration. Briefly, whole flies from each line were snap frozen in liquid nitrogen and RNA was extracted using the miRNeasy kit (Qiagen) with the following modifications: flies were homogenized in Qiazol reagent (Qiagen) by vortexing with stainless steel bearings and DNAse treatment was performed in a column using DNAseI (Qiagen). RNA was eluted in 30 µl of RNAse-free water and was quantified using a Nanodrop spectrophotometer. RNA quality was assessed via formaldehyde-agarose gel electrophoresis. cDNA was synthesized using the High-Capacity RNA-to-cDNA kit (Thermo-Fisher) as per manufacturer's protocol. For non-RT samples (-RT), the same protocol was followed for each sample and run alongside the + RT sample but without the reverse transcriptase. To measure relative transcript levels, qPCR was run on the -RT and + RT samples using NEBNext High-fidelity PCR master mix (New England Biolabs) with SYBR green-labeled primers directed toward tau and the housekeeping gene SDHA. The qPCR was performed using the BioRad CFX Connect RT-PCR detection system (Biorad). The cycling parameters for SDHA were: 95°C 3 min (95°C 30 s, 72°C 30 s, 72°C 1 min) × 40, melt curve: 72°C to 95°C, in 0.5°C increments. The PCR primers for SDHA included Forward (5′—CACCGATTCCGCGCTAAGAA—3′), and Reverse (5′—AGCCCTCGGTGATGAGACAT—3′). The cycling parameters for tau0N4R were: 95°C 3 min, (95°C 30 s, 52°C 30 s, 72°C 1 min) × 40, melt curve: 52°C to 95°C, in 0.5°C increments. The PCR primers used for tau0N4R included: forward (5′—CCATGCCAGACCTGAAGAAT—3′) and reverse (5′—TCTTGGCTTTGGCGTTCT—3′). Contributions of gDNA were controlled for by determining amplification efficiency for each run and calculating the percent of signal from gDNA. Samples with less than 4% signal from gDNA were used in the analysis and ΔCT values were calculated based on the difference between the mean Cq (SDHA) and mean Cq (tau) for each sample.

## Results

### Quantification of Aβ- and tau-induced degeneration in the fly eye

Our pipeline accurately identified ommatidia within a ROI and measured geometric features of individual ommatidium within the ROI including the radii, perimeter, circularity, and relative location. These values were then analyzed for downstream metrics such as the mean and standard deviation of each measurement as well as the distance to nearest neighboring ommatidium (see *Materials and methods*). Flies expressing Aβ42 and tau in the eye exhibited a noticeable rough eye phenotype caused by fused and pitted ommatidia that corresponded with increased ommatidial area, perimeter, and radii measurements as well as increased variation in each of these measurements (Fig. 1a and Supplementary Fig. 4). Altogether, we obtained a readout of 16 metrics which together encompass the integrity and degree of degeneration of individual ommatidia as well as the ommatidial array (Fig. 1a). When measured across genetic backgrounds, we find these 16 traits are highly, but not completely, correlated to each other (Supplementary Fig. 2), consistent with the idea that there exist both shared and independent processes and genetic loci involved in trait phenotype. Broad-sense heritability estimates for each trait indicate that all 16 traits are modestly heritable, with 15 showing a heritability *H*^2^ ≥ 0.05 (Supplementary Fig. 3). These phenotypic measurements specifically reflect toxicity induced by expression of Aβ42 and Tau in different genetic backgrounds, not an inherent property of DGRP strains, as those measurements in the WT fly eyes did not show the same degree of between-strain difference (Supplementary Fig. 4). We compared the scores generated by our pipeline to those assessed by humans for 200 images that span the severity of degeneration using the BLUP for each line. Human scoring was on a scale of 0 (completely WT), to 4 (severely affected) based on the degree of ommatidial pitting, fusion and spacing (Supplementary Fig. 5). Of the 16 traits measured by our pipeline, 15 showed significant correlation between the individual traits assessed by our pipeline and by humans (Fig. 1b, *P* < 0.01, Spearman’s *ρ*). We chose to focus our analysis on traits that were both significantly correlated to human scores as well as traits with a *H*^2^ > 0.05, leaving 14 traits (ecc mean, nn mean, area mean, radius_mean_ mean, perimeter mean, nn SD, area SD, radius_mean_ SD, perimeter SD, radius_min_ SD, radius_max_ mean, radius_max_ SD, radius_SD_ mean, radius_SD_ SD) that form the basis of our subsequent analyses. We further subdivided these traits into 5 “central” traits (ecc mean, nn mean, area mean, radius_mean_ mean, perimeter mean) and 9 “dispersion” traits (nn SD, area SD, radius_mean_ SD, perimeter SD, radius_min_ SD, radius_max_ mean, radius_max_ SD, radius_SD_ mean, and radius_SD_ SD).

### Expression of both Aβ42 and tau in the fly eye exacerbates toxicity and worsens with age

We quantified the degree of degeneration in flies expressing Aβ42 alone, tau alone and in flies simultaneously expressing both Aβ42 and tau under control of the GMR driver in the *w^1118^* background (our R32 triple transgenic donor line) at 28 days of age. To assess the influence of transgenes on eye degradation, we used a fixed-effect interaction model to quantify the individual additive effects of Aβ42 and tau and the nonadditive interaction between transgenes. While the Aβ42 strain we used has been shown to exhibit an age-dependent rough eye phenotype (Finelli *et al*. 2004), we found that expression of Aβ42 alone in a *w^1118^* background resulted in minimal disruption of eye morphology, exhibiting a nonsignificant effect (*P* > 0.05) in 4 out of 5 of our central trait measurements. Conversely, the expression of tau alone in the same *w^1118^* background significantly affected the ommatidial organization of the fly eye across all 14 traits (Fig. 2a and b). Simultaneous expression of both Aβ42 and tau significantly increased the degree of degeneration beyond what was measured in flies expressing Aβ42 or tau alone (Fig. 2a and b), and all 14 traits measured exhibited a significant epistatic effect from both transgenes on eye phenotype when assessed using a fixed-effect interaction model (*P* < 0.001, Fig. 2b and Supplementary Fig. 8).

To determine the effect of age on Aβ and tau-induced degeneration in diverse genetic backgrounds and in the *w^1118^* background (R32), we followed a single cohort of flies and measured degeneration at 4 d post-eclosion and again at 28 d post-eclosion. We found, on average, age led to a significant increase in degeneration based on BLUP values in 4 of the 5 central traits measured (Fig. 2c, *P* < 0.05 Student’s *t*-test), with expression in the *w^1118^* background also consistently affected by age (Fig. 2c, red line). While age-dependent degeneration has been reported in the *w^1118^* background (Finelli *et al*. 2004; Crowther *et al*. 2005; Fernandez-Funez *et al*. 2015), expression within the DGRP backgrounds indicates that not all lines exhibit an age-dependent effect for all traits, with some lines exhibiting an inverse age-effect, or no effect at all (Fig. 2c, gray lines).

### Aβ42- and tau-induced rough eye phenotype is dependent on genetic background

**Fig. 3.**
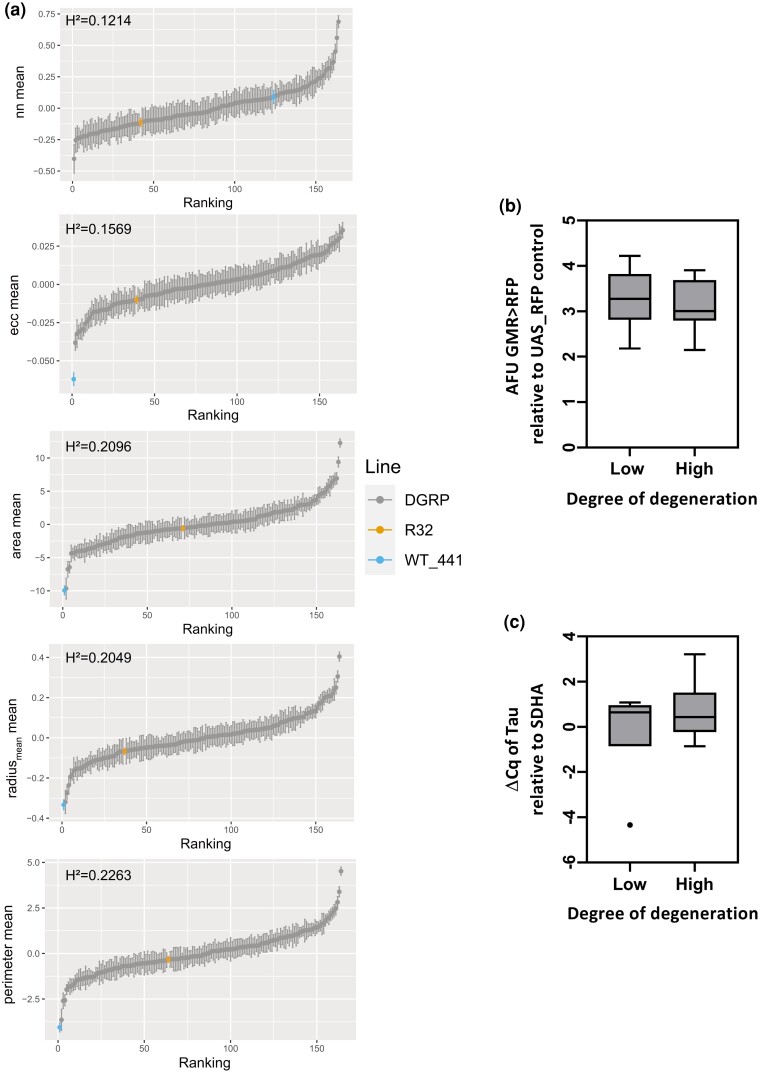
There is significant variation in Aβ- and tau-induced toxicity due to genetic background. Rank-ordered scores for 5 central traits show significant variation in degree of degeneration for each trait across DGRP lines (data from all traits available in Supplementary Fig. 8). Gray box and whisker plots are BLUP values derived from replicates within a line of the DGRP with error bars indicating the SE for each BLUP. The yellow boxplot is the BLUP value for the triple transgenic donor line (R32) and the blue boxplot is a representative WT eye from a nontransgenic DGRP line 441. Significance was determined by broad-sense heritability estimates (H^2^) b) Expression of RFP driven by GMR-Gal4 in the DGRP background of 12 lines ranked as highly degenerated (High) when Aβ and tau is expressed, and RFP expression driven by GMR-Gal4 in the DGRP background of 11 lines ranked as having a low degree of degeneration (low) when Aβ and tau are expressed. Fluorescence was measured using 580 nm excitation and 610–635 nm emission wavelengths. AFUs for the experimental flies were normalized to the mean AFU from the control (non-RFP-expressing) flies and the resulting AFU value is shown as a box and whisker plot. c) Box and whisker plots showing expression of tau driven by GMR-Gal4 in the DGRP background of 6 lines ranked as highly degenerated (high) and 7 lines ranked as less degenerated (low) based on ranked PC1 values. dCq was determined relative to the reference gene SDHA.

Using a linear mixed model, we assessed the effect of natural genetic variation on the Aβ42- and tau-induced degenerative eye phenotype by determining the broad sense heritability of the BLUPs for each of the traits in the 162 DGRP backgrounds tested (gray boxplots, Fig. 3a), including a WT eye (blue boxplot) and our triple transgenic donor line in the *w^1118^* background (R32, orange boxplot) for reference. We observed significant genetic variation and heritability in the degree of eye degeneration caused by expression of Aβ42 and tau in all 14 traits measured, with genetic background modifying the degree of degeneration compared to our lab-based strain (R32, orange boxplot) in both directions (Fig. 3a and Supplementary Fig. 3, Supplementary Fig. 9).

Although these results are consistent with the hypothesis that genetic background affects the processing and pathogenic mechanisms induced by Aβ42 and tau, the rough eye phenotype in Aβ42 and tau-expressing flies has been shown to be dose-dependent, and we wanted to determine the extent to which the effect of natural variation we observed was due to differences in expression of the transgenes. Flies homozygous for GMR-Aβ42 or for tau emerge at a reduced frequency in our triple transgenic line, indicating lethality (Supplementary Fig. 10, χ^2^ = 548.688, df = 3, *P* < 0.0001). Among those flies that do survive, homozygous flies exhibit severely affected eyes with a significantly decreased eye volume and increased degeneration (Supplementary Fig. 10). To ensure our measurements of variation on the effect of background on ommatidial integrity reflected the modulation of pathogenesis rather than natural variation in transgene expression, we performed 2 experiments to compare transgene (GMR-Gal4 and UAS-tau) induction in backgrounds with both high levels of degeneration and in lines with low levels of degeneration. The level of GMR-Gal4 expression was determined by quantification of a UAS-responsive fluorescent reporter. Quantification and comparison of fluorescence between DGRP backgrounds that exhibited low levels of degeneration (low) and backgrounds that exhibited high levels of degeneration (high) revealed that there is no significant effect of DGRP background on the Gal4 driver strength (Fig. 3b, Student's *t*-test, *P* = *0.67*). To confirm that comparable levels of Gal4 expression in High and Low lines resulted in comparable levels of transgene expression, we performed RT-qPCR measuring tau in High and Low lines. Our RT-qPCR results indicate there is no significant difference in the level of tau expression between highly-degenerated and less-degenerated DGRP lines (Fig. 3c, Student's *t*-test *P* = 0.42).

### Multitrait SNP-level genome-wide association analysis reveals modifiers of Aβ42- and tau-mediated toxicity

**Fig. 4.**
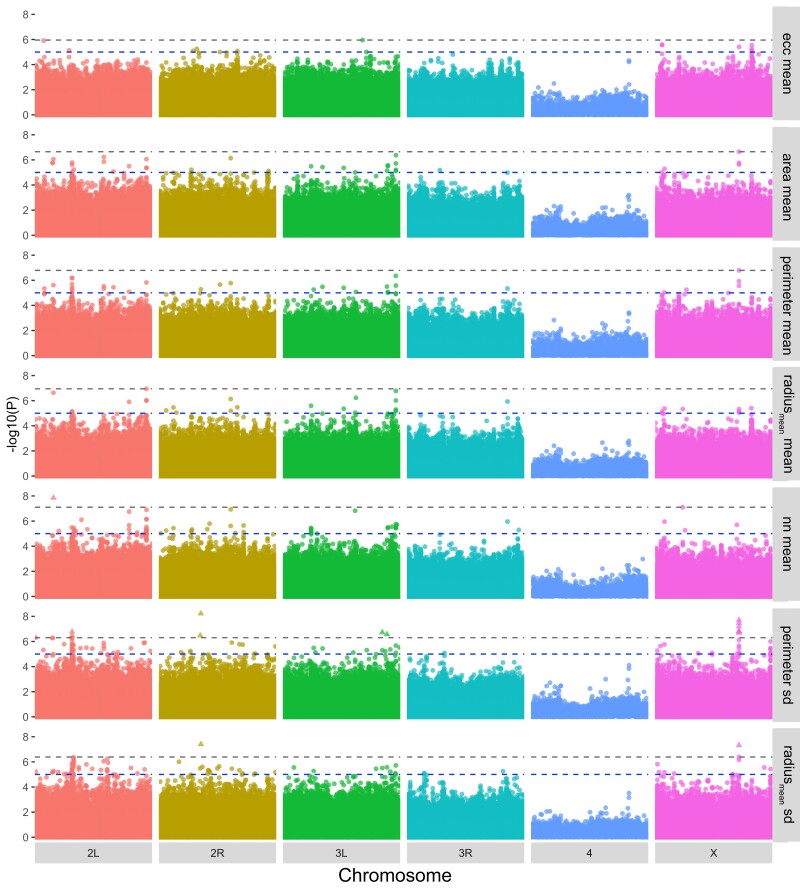
GWAS across traits identifies numerous SNPs associated with Aβ-and tau-induced ommatidial degeneration. Stacked Manhattan plots of SNP-level GWAS for 7 of 14 traits. Data for all 14 traits are shown in Supplementary Fig. 10. Each point corresponds to a SNP along the *Drosophila* chromosomes and its −log10 SNP-level *P* value [−log10 (*P*)]. Suggestive association of *P* < 1 × 10^−5^ as well as the BH-corrected false discovery rate (FDR) FDR = 0.05 are both indicated (upper and lower dashed lines, respectively).

Given the significant effect of genetic background on the distribution of fly eye scores in the traits measured, we hypothesized that natural genetic variation associated with genes in DGRP lines might underlie the pattern we observed. To test this, we performed a GWAS across each trait to determine if any genetic variants were significantly associated with the degree of ommatidial degeneration. Our GWAS identified 297 unique, suggestive SNPs, insertions, or deletions with *P* < 10^−5^ across the 14 traits measured (Fig. 4, Table 1 and Supplementary Fig. 11, Supplementary Table 1). After correcting for multiple testing, 14 unique SNPs reach statistical significance (*P*.*adjust* < 0.05; Fig. 4 and Table 1). Six of these SNPs are clustered in a 545 bp region on the X chromosome with the 8 remaining significant SNPs mapping to unique chromosomal locations (Table 1). While 2 of the 14 significant SNPs reached significance in both the perimeter SD and radius_mean_ SD traits and one reached significance in the nn mean trait, the remaining 11 significant SNPs are associated with variation in ommatidial perimeter (perimeter SD). Looking more broadly at our suggestive and significant SNPs, we find that 54% of SNPs (161/297) identified across the 14 traits are uniquely identified from a single trait, with the remaining 46% of SNPs reaching *P* < 10^−5^ in 2 or more traits (Supplementary Fig. 12). Interestingly, there is a single significant SNP (X_4359575_SNP) that reaches a suggestive *P* value in 12 out of 14 traits, and we find this SNP in our list of 16 associations that retain significance after BH-correction (Table 1).

**Table 1.** Significant SNP associations identified.

SNP	Flybase ID	Gene	Name	*P*	*P*_adjust_	Trait
2L_12758562_SNP	manualID: FBgn0032456	*MRP*	Multidrug-Resistance like Protein 1	1.404E−08	0.026549191	mean nn
2L_16046650_SNP	FBgn0000182	*BicC*	Bicaudal C	0.000000168	0.039559051	SD perimeter
2L_16058280_SNP	FBgn0013433	*beat-Ia*	Beaten path la	3.229E−07	0.048539694	SD perimeter
2R_16204841_SNP	FBgn0034501	*CG13868*	CG13868	3.337E−07	0.048539694	SD perimeter
2R_16322804_SNP	FBgn0086604	*side-VIII*	Sidestep VIII	5.963E−09	0.011275842	SD perimeter
2R_16322804_SNP	FBgn0086604	*side-VIII*	Sidestep VIII	3.971E−08	0.044513387	SD mean_radius
3L_7331392_SNP	FBgn0035766	*eco*	Establishment of Cohesion	1.891E−07	0.039559051	SD perimeter
3L_8030270_SNP	FBgn0011817	*nmo*	Nemo	2.693E−07	0.046294335	SD perimeter
X_4359575_SNP	FBgn0052773	*lncRNA:CR32773*	Long noncoding RNA:CR32773	1.982E−08	0.018739493	SD perimeter
X_4359702_SNP	FBgn0000179	*bi*	Bifid	6.387E−08	0.030194032	SD perimeter
X_4360110_SNP	FBgn0000179	*bi*	Bifid	1.514E−07	0.039559051	SD perimeter
X_4360113_INS	FBgn0000179	*bi*	Bifid	1.514E−07	0.039559051	SD perimeter
X_4360117_SNP	FBgn0052773	*lncRNA:CR32773*	Long noncoding RNA:CR32773	2.092E−07	0.039559051	SD perimeter
X_4360120_INS	FBgn0000179	*bi*	Bifid	1.686E−07	0.039559051	SD perimeter
X_4361594_SNP	FBgn0052773	*lncRNA:CR32773*	Long noncoding RNA:CR32773	3.394E−08	0.021393151	SD perimeter
X_4361594_SNP	FBgn0052773	*lncRNA:CR32773*	Long noncoding RNA:CR32773	4.708E−08	0.044513387	SD mean_radius

List of significant SNPs with the corresponding annotated gene, gene name, *P*-value, BH-corrected *P*-value (P_adjust_), and trait measured. Significance was determined by *P*_adjust_ values where *P* < 0.05.

We mapped the 14 significant SNPs and 283 suggestive SNPs to functionally annotated genes using Flybase release 5.57. The 283 suggestive SNPs mapped to 207 genes and the 14 significant SNPs mapped to 9 unique genes significantly associated with Aβ- and tau-induced ommatidial degeneration. Ten of our significantly associated variants map to genomic locations containing more than one gene. In those cases, we have listed the primary gene annotation (gene 1) as output by the DGRP GWAS pipeline (Table 1 and Supplementary Table 1), (Mackay *et al*. 2012). *Bifid* and *lncRNA:CR32773*, genes involved in eye development and size determination respectively, are found on opposite strands of the X chromosome spanning the region represented by 6 of our significantly associated SNPs. A single significant SNP (2L_12758562_SNP) was not annotated to any particular gene in our analysis, but falls within a noncoding region of *Multidrug-Resistance like Protein 1* (*MRP*), whose mammalian ortholog is a transporter that has been shown to export Aβ from the endothelium of the blood brain barrier (Krohn *et al*. 2011; Jepsen *et al*. 2021). Two of our significant hits, *beaten path-Ia* (*beat-la*) and *sidestep-VIII* (*side-VIII*), are members of 2 immunoglobulin superfamilies that play a role in neuromuscular development (Li *et al*. 2017). In addition to *Bifid*, other significant hits (*eco*, *nemo*, and *BicC*) are associated with cellular proliferation and organ development (Williams *et al*. 2003; Chicoine *et al*. 2007; Merino *et al*. 2009). Interestingly, *eco* has also been identified as a modifier of *Drosophila* longevity, and *nemo* has recently emerged as a novel modifier of the protein aggregation disease spinal bulbar muscular atrophy (Paik *et al*. 2012; Todd *et al*. 2015). While CG13868 has no known function, it is differentially expressed in brain pacemaker neurons and contains a transcriptional binding site for factors that drive circadian cycles in *Drosophila* (Rivas *et al*. 2021). Altogether, genes mapped by significant SNPs appear to represent neuronal and developmental processes, including neuronal development, organ development and signal tranSDuction (Table 1 and Supplementary Table 1).

**Fig. 5.**
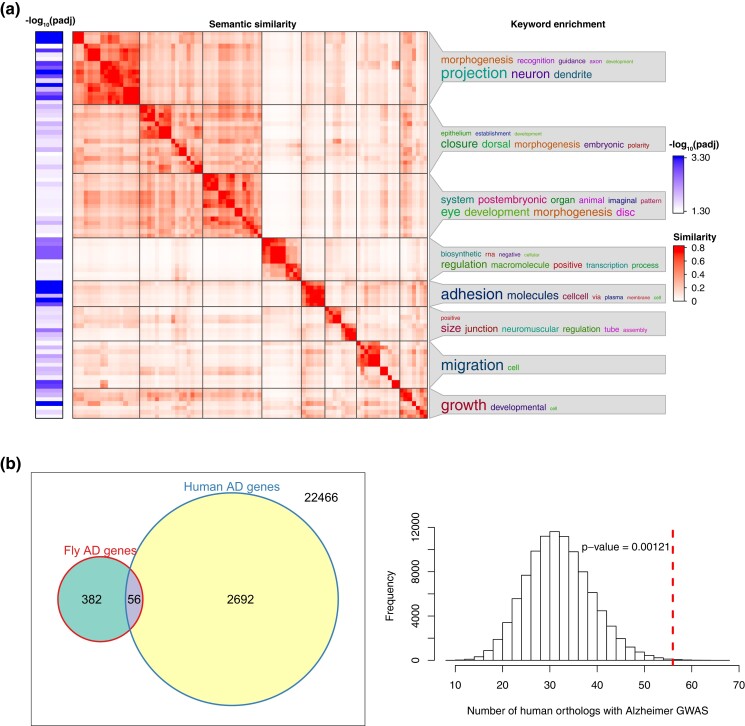
Suggestive SNPs are enriched for genes involved in neuronal and organismal development as well as for gene orthologs identified in human AD GWAS. a) Biological processes identified by GO term analysis on suggestive genes ordered by semantic similarity to identify relationships between nested terms. Inverse log10 of the adjusted *P* value is shown on the left, while enrichment for key words is shown on the right. Larger text correlates with higher representation of the key word. b, left panel) Venn diagram portraying the overlap between genes implicated in human GWAS for AD and human orthologs of fly genes identified as suggestive in our screen. The box encompasses the total number of orthologous relationships between fly and human genes. The right hand circle represents the 2,748 orthologous relationships where the human ortholog has been implicated in AD through GWA studies and the left hand circle represents the 438 orthologs mapped from our suggestive gene results. The purple intersect highlights the overlap and represents the number of orthologous genes where both the fly and human orthologs have been implicated in AD. b, right panel) A histogram showing the results of the permutation test and the expected distribution for the number of orthologous relationships (i.e. fly and human gene pairs) where both genes have been implicated in AD (permutation test, *P* = 0.00121, where *P* = 1 + the number of randomized values equal to or greater than the observed value of 56, divided by 1 + the number of permutations).

To determine if there were any functional gene groups over-represented within our data, we performed GO enrichment analysis on the 207 genes associated with our suggestive SNPs (*P* < 10^−5^). This analysis revealed significant enrichment in a number of biological processes, molecular functions, and cellular components (Supplementary Table 2, *P*.*adjust* < 0.05). Since many of the biological processes we identified are nested in hierarchical categories, we clustered our results based on the Wang method of semantic similarity. The most significant enrichment in biological processes occurred in genes related to neuronal projection morphogenesis, cell-cell adhesion, growth, and eye development (Fig. 5a and Supplementary Table 2). These biological processes are mirrored in our molecular function and cellular component results, with our suggestive genes exhibiting significant enrichment in genes involved in ion and neurotransmitter transmembrane transport and in genes associated with the plasma membrane.

To determine the extent to which the results of our screen represent potential modifiers or risk factors for disease in humans, we identified human orthologs for each of our suggestive genes and queried the number of orthologs that have a suggestive or significant association with AD in published human GWA studies. Of the 388 human genes identified via orthologous relationships to *Drosophila* genes mapped in our screen, 56 have previously been associated with AD in the NHGRI-EBI GWAS catalog, including 4 genes in well-established AD loci (*ADAM10, ECHDC3, CELF1, SCARB2*) (Kamboh 2022), and 20 more loci that have been identified in 2 or more studies (*AFF1, AFGFG2, ASIC2, CELF2, DLG2, DLG4, FOXL1, FOXQ1, GALNT17, HIVEP3, HS3ST4, KNCIP1, KNCIP4, MUC2, RBFOX1, SCARB1, SDK1, SGIP1, SLC28A1, SPA17*). We used a permutation test to compare the number of genes in our data set with the number of orthologous human AD genes one would expect from randomly sampling 207 genes from the fly genome. A million permutations of randomly sampled genes resulted in a distribution centered around 32. Our identification of 56 genes in our dataset that have been associated with AD in humans shows significant enrichment for AD associated genes (permutation test, *P* = 0.00121), representing an 80% increase in the number of genes associated with AD in human GWAS studies than expected by random sampling (Fig. 5b).

## Discussion

We have found that expression of Aβ42 exacerbates tau-mediated degeneration in the fly eye, and that there is natural variation in response to Aβ and tau-induced degeneration. We confirmed that phenotypic differences observed are due to differences in the effect of genetic background on Aβ42- and tau-induced toxicity rather than on differences in expression of our transgenes, and identified a number of genes that significantly associate with ommatidial disorganization caused by Aβ42 and tau. Our analysis of suggestive genes implicates processes specific to neuronal development and maintenance in governing the changes observed across backgrounds. We also find enrichment in our suggestive genes for AD-associated human orthologs. The results from these enrichment analyses provide bioinformatic validation for our approach, further establishing *Drosophila* as a valuable and tractable model from which to validate and inform human GWA studies.

The amyloid cascade hypothesis has long dominated our understanding of AD, and there is a wealth of evidence linking altered Aβ production to aggregation and toxicity. However, accumulating evidence highlights the effects of tau, as well as alternative mechanisms such as inflammation, dysregulated Ca2 + signaling, transcriptional dysregulation, and altered neuronal activation as potential causative factors underlying disease pathogenesis in LOAD. Several of these dysregulated pathways have been shown to be exacerbated by expression of both Aβ and tau. Aβ can induce and increase tau oligomer formation and seeding (Lasagna-Reeves *et al*. 2010; Vasconcelos *et al*. 2016) as well as enhance transcriptional changes induced by tau (Pickett *et al*. 2019; Sierksma *et al*. 2020), and there is evidence that down-regulating tau in an amyloidogenic mouse model decreases characteristic inflammation (Pickett *et al*. 2019). Relatively few animal models exist that allow researchers to explore potential interactions between these 2 proteins and much of what we know about disease pathogenesis comes from monogenic disease models based on EOAD. Data from our model combining 2 pathogenic effectors of AD indicate that expression of tau drives a majority of the rough eye phenotype observed and that expression of Aβ42 exacerbates degeneration in a nonlinear fashion. This provides additional support for a synergistic interaction between Aβ and tau, though the mechanism by which expression of these 2 proteins leads to exacerbated degeneration is unclear.

While expression of Aβ has been reported to lead to age-dependent ommatidial degeneration in *Drosophila* models of AD, the expression of tau in the eye alone does not exhibit the same age-dependent changes, despite progressive age-related degeneration reported in other tissue types (Jackson *et al*. 2002; Finelli *et al*. 2004; Khurana 2008). Our analysis indicates that susceptibility to age-dependent changes in response to Aβ and tau may vary depending on genetic background. We performed our screen at 28 days of age, an age that encompasses both developmental and age-dependent effects on eye degeneration, and a significant rough eye phenotype is observed upon eclosion in our triple transgenic donor flies. Although we see an overall worsening of eye phenotype with age across traits, this is not the case in all lines, indicating that there may be a genetic component regulating the age-dependent degeneration that is separate from the developmental degeneration observed on eclosion. Although our aging study was underpowered to be able to dissect variants underlying ommatidial disorganization at 4 d of age vs 28 days of age, a comparison of variants associated with developmental effects vs age-specific effects may prove informative.

Polymorphisms that affect complex traits likely affect multiple traits, and GWAS across multiple, correlated traits is emerging as a powerful tool to help dissect complex genetic traits (Bonnemaijer *et al*. 2019; Julienne *et al*. 2021; Merrick *et al*. 2021). By looking for genes with pleiotropic effects, we hoped to increase the accuracy of our findings, reducing error and increasing sensitivity for causal variants within a quantitative trait locus. We performed GWAS analysis across a set of correlated traits associated with the rough eye phenotype rather than using 1 summarized score for 3 reasons. First, complex traits are controlled by many genes and environmental factors, whose macroscopic phenotypic characterization may relate to distinct biological processes (Sella and Barton 2019). Measuring the phenotype across multiple metrics may better capture the underlying biology and may serve to increase discovery of underlying causal variants (Pendergrass *et al*. 2011; O’Reilly *et al*. 2012). Second, genome-wide association analyses provide a powerful tool in dissecting complex traits. However, their success depends on the underlying genetic architecture and trait heritability, and it remains a challenging task in the case of a genetic architecture composed of a large number of small-effect loci or a trait with low heritability (Bush and Moore 2012). The genetic architecture of AD in humans has been suggested to be multifactorial, with large numbers of genes with small effect size (Andrews *et al*. 2023). The degenerative fly eye-related traits in our study showed low heritability, making it more difficult to detect small-effect loci. To enhance the ability to detect signals from small-effect loci, one approach is to increase sample size, which is often costly and sometimes not feasible. Another option is to take a multitrait approach, analyzing multiple related traits jointly (Chhetri *et al*. 2019; Julienne *et al*. 2021). One of the advantages of multitrait GWAS is that missing information in one phenotype in the multitrait set can be complemented by the other phenotypes, boosting the discovery of genetic variants associated with traits of interest (Ritchie *et al*. 2015). Also, multitrait GWAS improves the ability to detect susceptible pleiotropic genetic variants even when the traits have low correlation (Broadaway *et al*. 2016). Third, while there are several statistical frameworks for multitrait GWAS analysis (O’Reilly *et al*. 2012; Zhou and Stephens 2014), we took a more direct and interpretable approach. We performed GWAS on each trait to obtain significant associated genetic variants and their mapped genes. The analysis presented here allows us to identify loci common across traits as well as those unique to specific traits.

Perhaps not surprisingly, given the correlation of the traits analyzed, 46% of our suggestive SNPs are suggestive in 2 or more traits, potentially representing core processes involved in eye development (Supplementary Fig. 12) while the remaining SNPs may represent specific processes that shape independent features. Interestingly, 8 of 16 of our significant SNPs (*P*_adj_ < 0.05, Table 1) represent loci that reach *P* < 10^−5^ in 8 or more traits, and many of these appear to affect development. *Bifid* and *lncRNA:CR32773* are genes on opposing strands of the same genomic region, both of which had a suggestive gene-level *P* value in 12 of the 14 traits analyzed, suggesting that one or both may play a significant role in eye morphology in response to Aβ and tau expression. In fact, *Bifid* is one of the genes driving enrichment in transcriptionally-associated molecular function and biological process GO categories (Supplementary Table 2), and plays a key role in *Drosophila* eye development, primarily through control of cell proliferation (Tsai *et al*. 2015). While the function of *lncRNA:CR32773* is less well understood, it has also been linked to control of animal size (Hevia *et al*. 2017; Watanabe and Riddle 2021). *Beat-Ia* and s*ide*-*VIII* are 2 additional genes with developmental roles mapped by our significant hits. These 2 genes are members of an immunoglobulin superfamily formed by paralogs of Beaten Path (Beat) and Sidestep (Side), a ligand-receptor pair that plays a central role in motor axon guidance. Researchers have identified 14 Beats paralogs and 8 Side paralogs, all neuronally expressed with specific temporal and spatial transcriptional profiles (Pipes *et al*. 2001; Li *et al*. 2017). Although many of the binding partners between the 2 groups have been identified, several Beat genes and Side genes are considered orphans with no binding partners in the other subfamily (Özkan *et al*. 2013; Li *et al*. 2017). *Beat-Ia* is the founding member of this superfamily, with a well-characterized effect on defasciculation and projection of motor axons to muscle targets requiring binding to its partner *sidestep* (Fambrough and Goodman 1996; Siebert *et al*. 2009). The association of eye phenotype with *side-VIII* is especially interesting, given that its binding partner is unknown, and unlike the other side proteins, its expression pattern is tightly restricted to a subset of neurons in the CNS (Li *et al*. 2017). Beyond *beat-Ia*, a number of other Beats proteins are mapped from our suggestive SNPs including *beat-Ic, beat-IIIc,* and *beat-Vc*. The functions of these specific Beat proteins are yet to be clearly defined, but their expression across numerous neural subtypes indicates they may act in combination and in regions to guide axonal processes.

The identification of developmental modifiers is also reflected in our GO analysis. Many of our enriched Biological Process GO terms are related to neuronal and organismal development, with the most significant enrichment seen in processes associated with neuronal projection morphogenesis. It is not surprising that our screen has identified several developmental modifiers, given the degree of degeneration observed upon eclosion in our triple transgenic flies. However, our results may offer important insights that extend beyond development and into pathogenesis.

Transcriptional regulation and RNA biosynthesis is another broad process implicated in our GO analysis, offering a potential mechanism by which synergistic interactions between Aβ and tau might occur. Our enriched Molecular Function GO terms also highlight the role of transmembrane transport, consistent with the enrichment we see for the proteins associated with the plasma membrane in our Cellular Compartments GO analysis. Imbalances in metal homeostasis have been shown to be closely related to onset and progression of AD (Bush 2013; Liu *et al*. 2019), and appropriate ionic trafficking and segregation creates and maintains the plasma membrane potential that regulates neurotransmitter release and is fundamental to neuronal function. In human AD patients, ionic disturbances are thought to occur early and contribute to dysregulated neuronal signaling. Recent proteomic analysis of the entorhinal cortex, a region that has been implicated as a site of early dysfunction in AD, has revealed a pattern of enrichment in ion transport proteins in AD patients (Jia *et al*. 2021) similar to what we observed in flies. Six of the genes driving significance in our MF enrichment are orthologs of human genes that code for members of the solute carrier superfamily (*Ndae1, DAT, CNT1, CNT2, List, Oatp26F*). In particular, *Ndae1* and *DAT* have been shown to have direct effects on neurotransmitter uptake and release through anion exchange and dopamine transport, respectively (Romero *et al*. 2000; Pörzgen *et al*. 2001).

Human GWA studies for AD associations have helped to identify numerous susceptibility loci, mostly from European populations (Marioni *et al*. 2018; Jansen *et al*. 2019; Kunkle *et al*. 2019). Approximately 20 of these have emerged as well-accepted risk factors that map to genes whose effect on AD-associated traits have been replicated or validated to varying extents. The enrichment detected in our suggestive dataset provides meaningful validation of our approach as one that can independently detect gene associations that have been identified by others.

It also provides an opportunity to mine a subset of genes associated with AD in humans with variable association strength, to identify causal gene associations, and to understand potential mechanisms by which causal genes affect susceptibility to disease. Four of our suggestive genes (*kuz, CG6984, bru2,* and *CG40006*) represent *Drosophila* orthologs of well-accepted risk factors in European populations (ADAM10, ECHDC3, CELF1, and SCARB2, respectively). ADAM10 is an alpha-secretase with a role in processing the APP, as well as in cleaving a wide range of substrates, including numerous cell-adhesion and membrane-bound proteins (Kuhn *et al*. 2016) as well as tau (Henriksen *et al*. 2013). ADAM10-derived tau fragments in CSF have been shown to inversely correlate with cognitive function in humans (Henriksen *et al*. 2013), and blocking ADAM10 in a nontransgenic AD mouse model leads to AD-like pathology, including hyperphosphorylation of tau and accumulation of Aβ aggregates (Epis *et al*. 2010). Given that there is no APP to cleave in our *Drosophila* model of AD, it is possible that the phenotype association detected in our assay is based on *kuz* interactions with tau. Moving forward, specificity and activity of the *Drosophila* metalloprotease will need to be determined.

*Drosophila* has been used for over a century to help identify cellular processes, mechanistic pathways, genes, and phenotypes that translate to humans (reviewed in Schneider 2000; Mackay and Anholt 2006; Gonzalez 2013; McGurk *et al*. 2015), and our results illustrate the utility of convergent data between species, providing independent evidence for both causal gene-associations and for putative modifiers that have not reached significance in human studies due to population-biased sampling. Although mapping SNPs to causal genes has thus far has been based on proximity to the associated SNP, many associations occur in nonprotein coding regions and it has been estimated that only about a third of trait-associated SNPs found in human GWAS have a functional association with the nearest gene (Porcu *et al*. 2019). *Drongo*, for example, is the *Drosophila* ortholog of AGFG2. Although AGFG2 was not associated with AD in 2 major meta-analyses (Jansen *et al*. 2019; Kunkle *et al*. 2019), it has recently been identified as being transcriptionally upregulated in AD patients in several independent datasets (Fernandez *et al*. 2021). NYAP1 is a gene-locus that has reached significance in a number of human AD GWAS. While there is some evidence that NYAP1 may be the causal gene, the phenotype-associated locus that maps to NYAP1 contains 53 genes including AGFG2 (Kunkle *et al*. 2019). The identification of this gene in a model organism with significant genetic similarity, but different genomic organization, provides independent and complementary support for this gene being of interest. The results presented here can also provide independent validation for GWAS results in under-studied human populations. ERO1A and UGT1A10 are 2 examples of genes that have been identified in AD GWAS specifically in African American populations, which were also identified as orthologous loci in our study (Mez *et al*. 2017). UGT1A10, a UDP-glucoronosyltransferase that plays a role in dopaminergic metabolism in human brain, and the prosurvival gene ERO1A have both been shown to be upregulated in response to tau (Frost *et al*. 2014; Poirier *et al*. 2019).

The results presented here make use of a model system that replicates synergistic interactions between Aβ42 and tau, allowing us to identify many modifiers that may operate through modulation of Aβ42 and tau interactions, highlighting developmental components and functional mechanisms by which naturally occurring genetic variation modifies susceptibility to Aβ42 and tau-induced toxicity. Neurons are uniquely susceptible to alterations in ion and neurotransmitter metabolism and uptake, and the enrichment seen in our suggestive gene list highlights the importance of neuronal morphogenesis and maintenance in AD. Throughout the EBI-GWAS catalog, over 2,000 human genes have been reported as being significantly or suggestively associated with AD. While some of these may be loci that are incorrectly attributed to a single nearby gene, there are also likely many true modifiers that do not reach genome-wide significance. While direct genetic validation is required for definitive identification of true modifiers, our findings illustrate the potential for unbiased multitrait screens in *Drosophila* to supplement and inform human studies, or vice versa, drawing from several sources and analyses to identify novel candidate modifiers of AD.

## Supplementary Material

jkad132_Supplementary_Data

## Data Availability

All strains used are available upon request. The authors affirm that all data necessary for confirming the conclusions of the article are present within the article, figures, tables, and Supplementary Materials. The R script used for the data analysis pipeline is available on our Github site: https://github.com/mingwhy/AD_fly_eye. Supplemental material available at G3 online.
