# Supplementary material for: Using Drosophila to identify naturally occurring genetic modifiers of amyloid beta 42- and tau-induced toxicity
Source: G3 (Bethesda). 2023 Jun 13;13(9):jkad132. doi: 10.1093/g3journal/jkad132 (PMC10468303; doi:10.1093/g3journal/jkad132)
Supplement: jkad132_Supplementary_Data [file jkad132_supplementary_data.zip › Figure_S10_G3-2023-404168.docx]

**Figure S10**

**
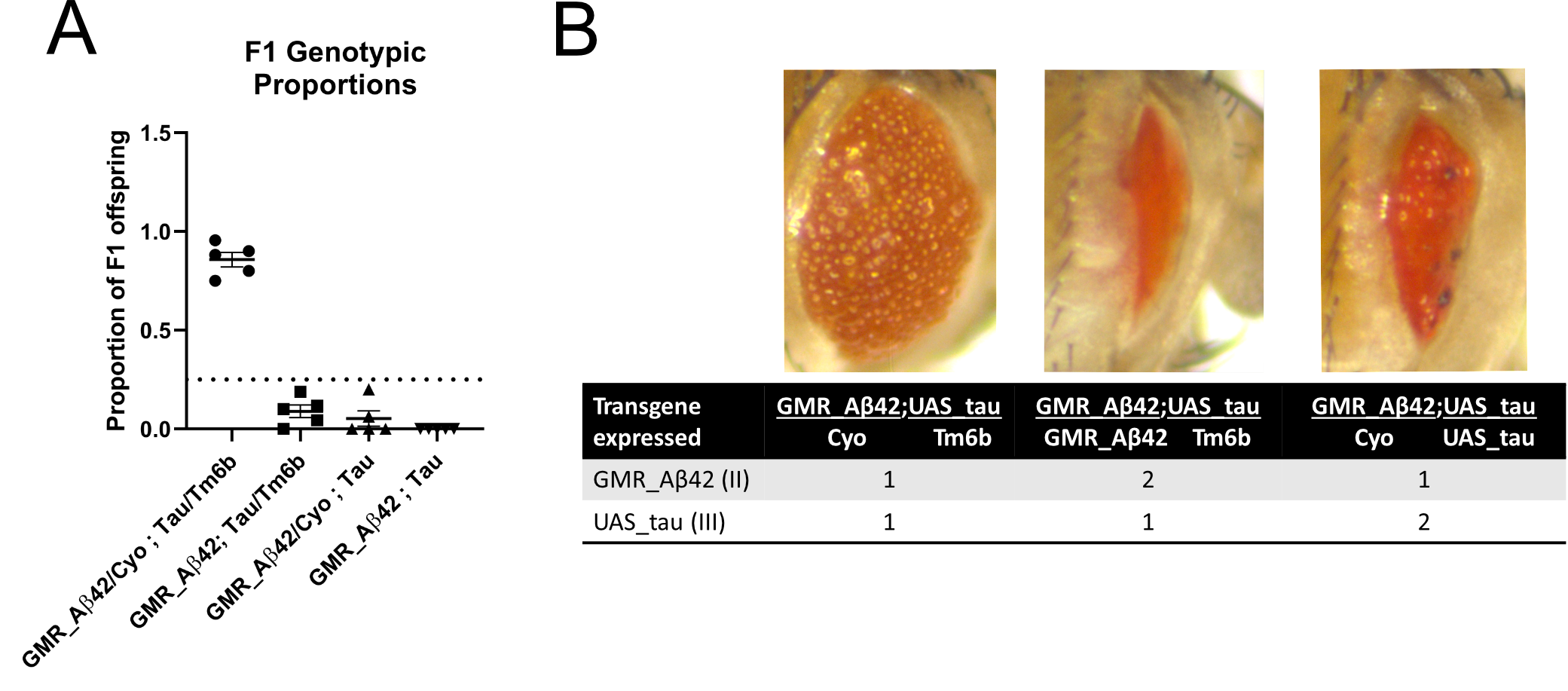
**

**Supplementary Figure S10. Homozygosity for either GMR and Aβ42 or tau results in increased toxicity and lethality.** A) Flies homozygous for either GMR_Ab42 or Tau emerge at a reduced rate. Proportion of flies of each genotype is indicated. Proportions were calculated as the number of flies of that genotype divided by the total number of F1 offspring. Data from 5 biological replicates is shown. Dashed line indicates a 0.25 proportion of the offspring, which is the expected ratio for each genotype shown. B) Homozygosity of either the second or the third chromosome results in severe eye toxicity. Above are representative images of flies with the given genotype. Below is a table indicating the number of transgenes carried by the individual fly.
