# Supplementary material for: Using Drosophila to identify naturally occurring genetic modifiers of amyloid beta 42- and tau-induced toxicity
Source: G3 (Bethesda). 2023 Jun 13;13(9):jkad132. doi: 10.1093/g3journal/jkad132 (PMC10468303; doi:10.1093/g3journal/jkad132)
Supplement: jkad132_Supplementary_Data [file jkad132_supplementary_data.zip › Figure_S11_G3-2023-404168.docx]

**Figure S11**


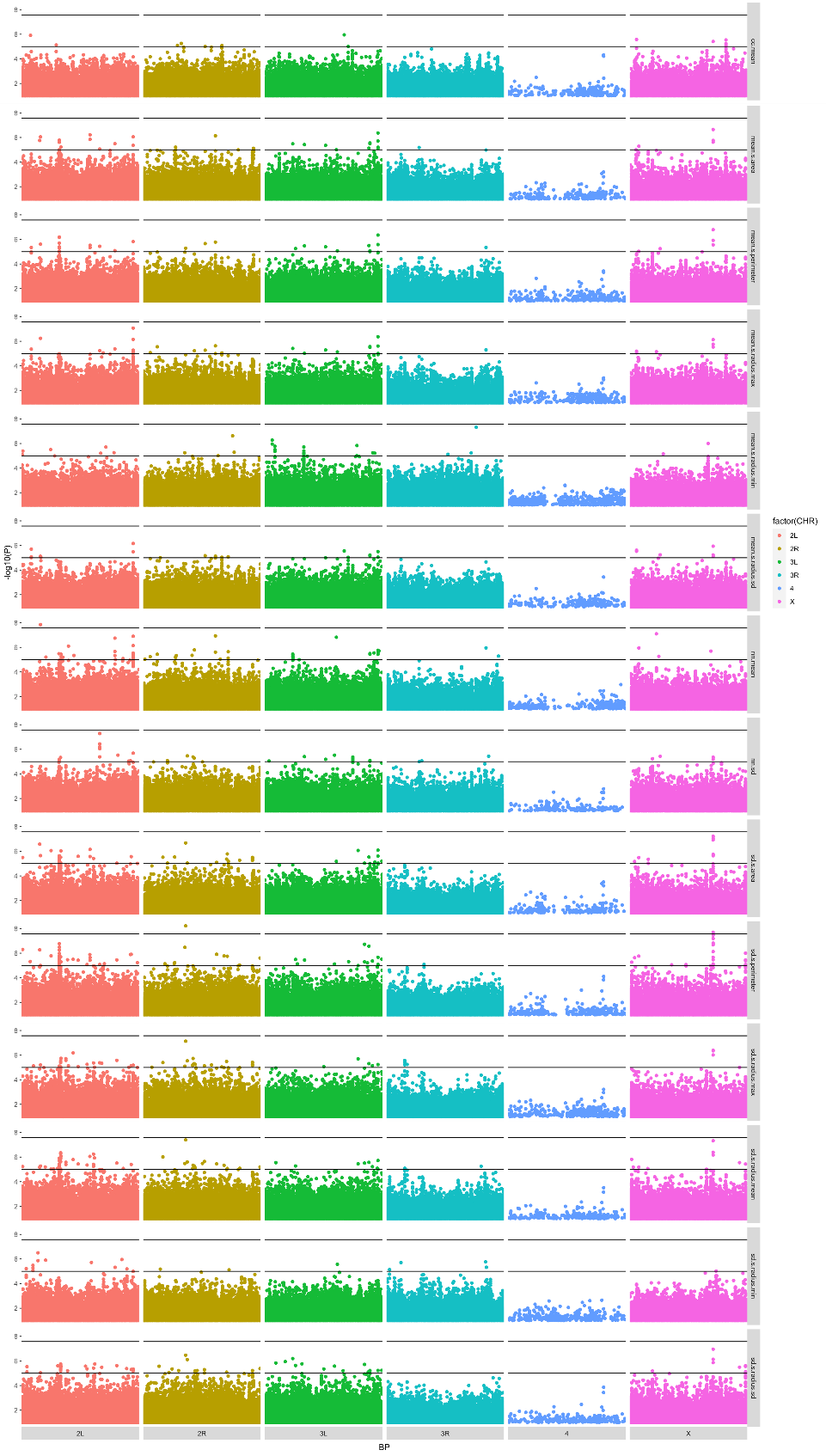


**Supplementary Figure S11. Stacked Manhattan plots of SNP-level GWAS analysis for all fourteen traits**. Each point corresponds to a SNP along the *Drosophila* chromosomes and its -log10 SNP-level P value (-log10 (P)). Arbitrary significance of *P* < 10^-5^ as well as the Benjamini-Hochberg corrected FDR are both indicated.
