# Supplementary material for: Using Drosophila to identify naturally occurring genetic modifiers of amyloid beta 42- and tau-induced toxicity
Source: G3 (Bethesda). 2023 Jun 13;13(9):jkad132. doi: 10.1093/g3journal/jkad132 (PMC10468303; doi:10.1093/g3journal/jkad132)
Supplement: jkad132_Supplementary_Data [file jkad132_supplementary_data.zip › Figure_S12_G3-2023-404168.docx]

**Figure S12**

**
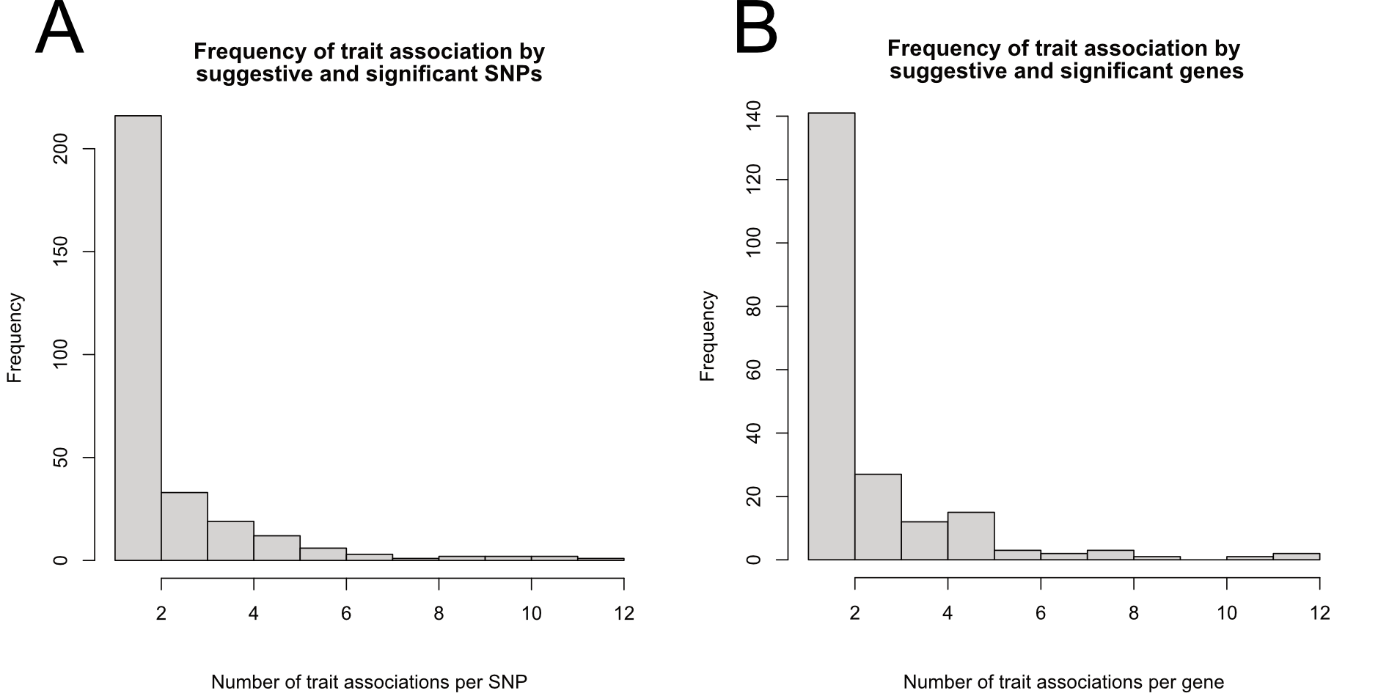
**

**Supplementary Figure S12. Frequency of trait association by suggestive and significant SNPs and genes.** A) Histogram depicting the number of SNPs that reach nominal significance (*P* < 10^-5^) in one or more traits. 161 out of 297 suggestive SNPs reach *P* < 10^-5^ in a single trait and 55 out of 297 reaching *P* < 10^-5^ in two traits. At the other end of the histogram, one out of 297 suggestive SNPs reach *P* < 10^-5^ in 12 of our 14 traits. B) Histogram depicting the number of genes mapped that reach *P* < 10^-5^ in one or more traits. 104 out of 207 suggestive genes reach *P* < 10^-5^ in a single trait and 37 out of 207 reaching *P* < 10^-5^ in two traits. At the other end of the histogram, two genes reach *P* < 10^-5^ in 12 of our 14 traits.
