# Supplementary material for: Using Drosophila to identify naturally occurring genetic modifiers of amyloid beta 42- and tau-induced toxicity
Source: G3 (Bethesda). 2023 Jun 13;13(9):jkad132. doi: 10.1093/g3journal/jkad132 (PMC10468303; doi:10.1093/g3journal/jkad132)
Supplement: jkad132_Supplementary_Data [file jkad132_supplementary_data.zip › Figure_S1_G3-2023-404168.docx]

**Figure S1**

**
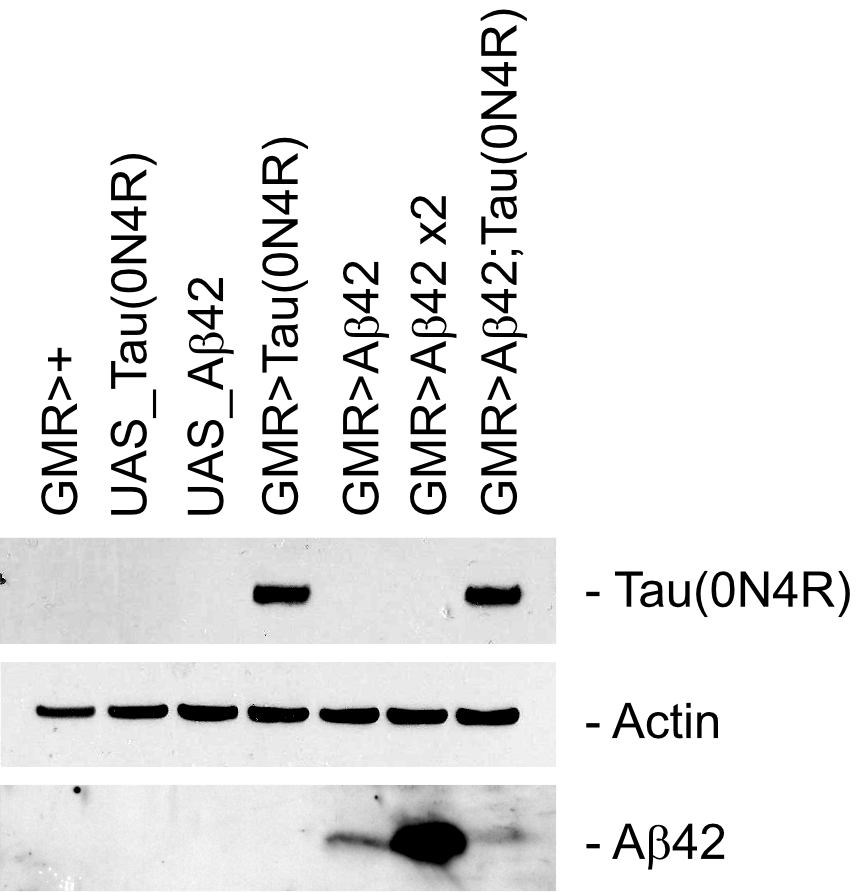
**

**Supplementary Figure S1. Validation of protein expression in AD fly model.** Western blot analysis of protein lysates prepared from the heads of flies expressing GMR-Gal4 alone (GMR>+), carrying UAS_Aβ42 or UAS_tau alone, GMR-Gal4 driving tau only (GMR>tau), GMR-Gal4 driving Aβ42 only (GMR>Aβ42), GMR-Gal4 driving two copies of Aβ42 (GMR>Aβ42x2), and our triple transgenic line with GMR-Gal4 driving expression of both Aβ42 and tau (GMR>Aβ42;tau). The blot was probed with antibodies specific to human tau, Aβ42 (6E10), and to actin as the loading control.
