# Supplementary material for: Using Drosophila to identify naturally occurring genetic modifiers of amyloid beta 42- and tau-induced toxicity
Source: G3 (Bethesda). 2023 Jun 13;13(9):jkad132. doi: 10.1093/g3journal/jkad132 (PMC10468303; doi:10.1093/g3journal/jkad132)
Supplement: jkad132_Supplementary_Data [file jkad132_supplementary_data.zip › Figure_S2_G3-2023-404168.docx]

**Figure S2**

**
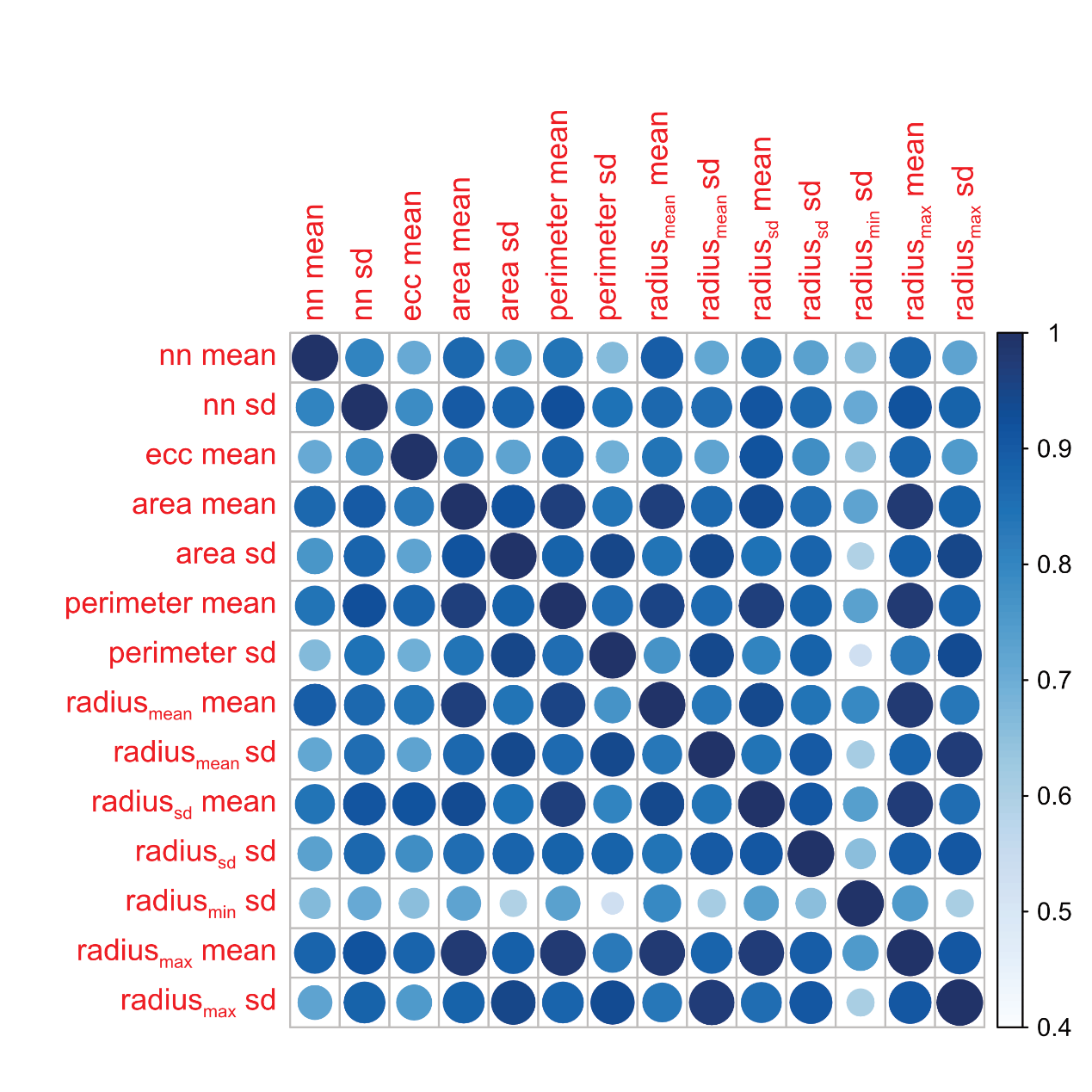
**

**Supplementary Figure S2. Correlation between traits.** Correlation of each of the 14 traits with saturation of color indicating positive (blue) and negative (red) correlations. Data based on trait BLUP values across the 162 DGRP lines measured.
