# Supplementary material for: Using Drosophila to identify naturally occurring genetic modifiers of amyloid beta 42- and tau-induced toxicity
Source: G3 (Bethesda). 2023 Jun 13;13(9):jkad132. doi: 10.1093/g3journal/jkad132 (PMC10468303; doi:10.1093/g3journal/jkad132)
Supplement: jkad132_Supplementary_Data [file jkad132_supplementary_data.zip › Figure_S3_G3-2023-404168.docx]

**Figure S3**

**
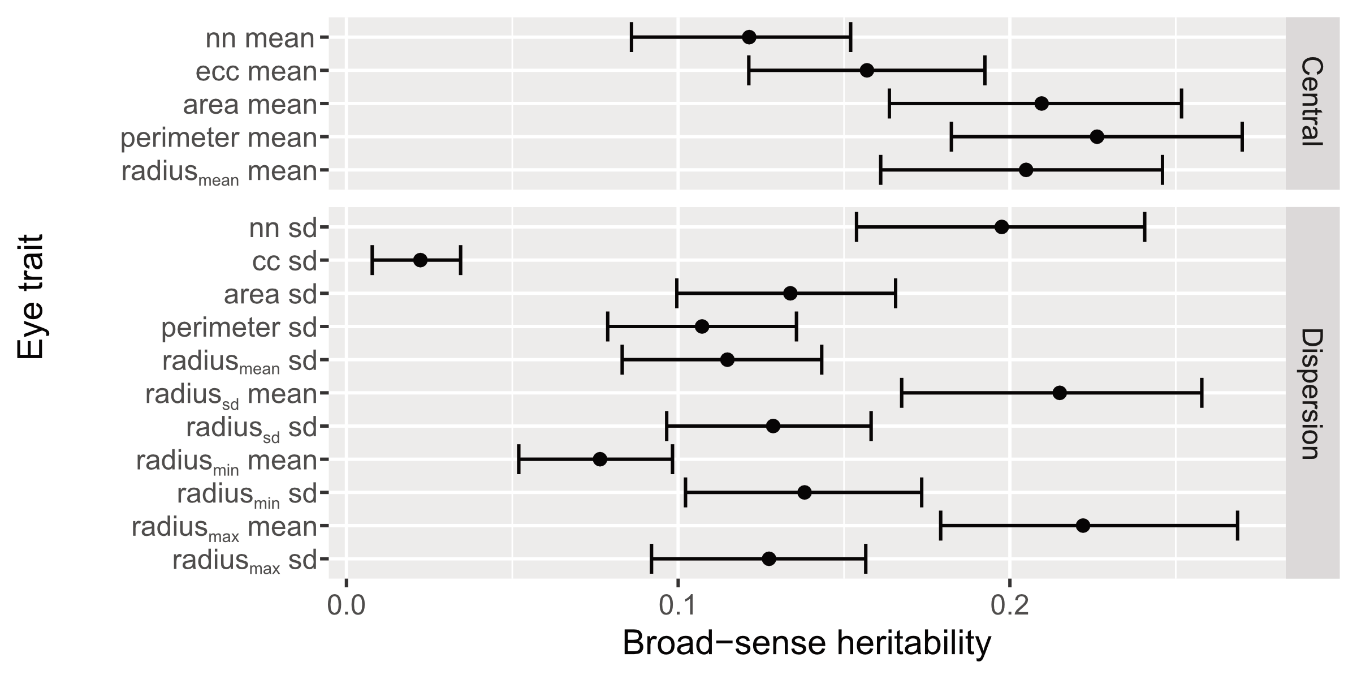
**

**Supplementary Figure S3. Broad-sense heritability estimates of extracted traits.** We estimated the broad sense heritability of each of the extracted traits. H^2^+SE for each trait measured is shown. Further analyses focus on the 14 traits with an H^2^ between 0.1 and 0.25.
