# Supplementary material for: Using Drosophila to identify naturally occurring genetic modifiers of amyloid beta 42- and tau-induced toxicity
Source: G3 (Bethesda). 2023 Jun 13;13(9):jkad132. doi: 10.1093/g3journal/jkad132 (PMC10468303; doi:10.1093/g3journal/jkad132)
Supplement: jkad132_Supplementary_Data [file jkad132_supplementary_data.zip › Figure_S4_G3-2023-404168.docx]

**Figure S4**


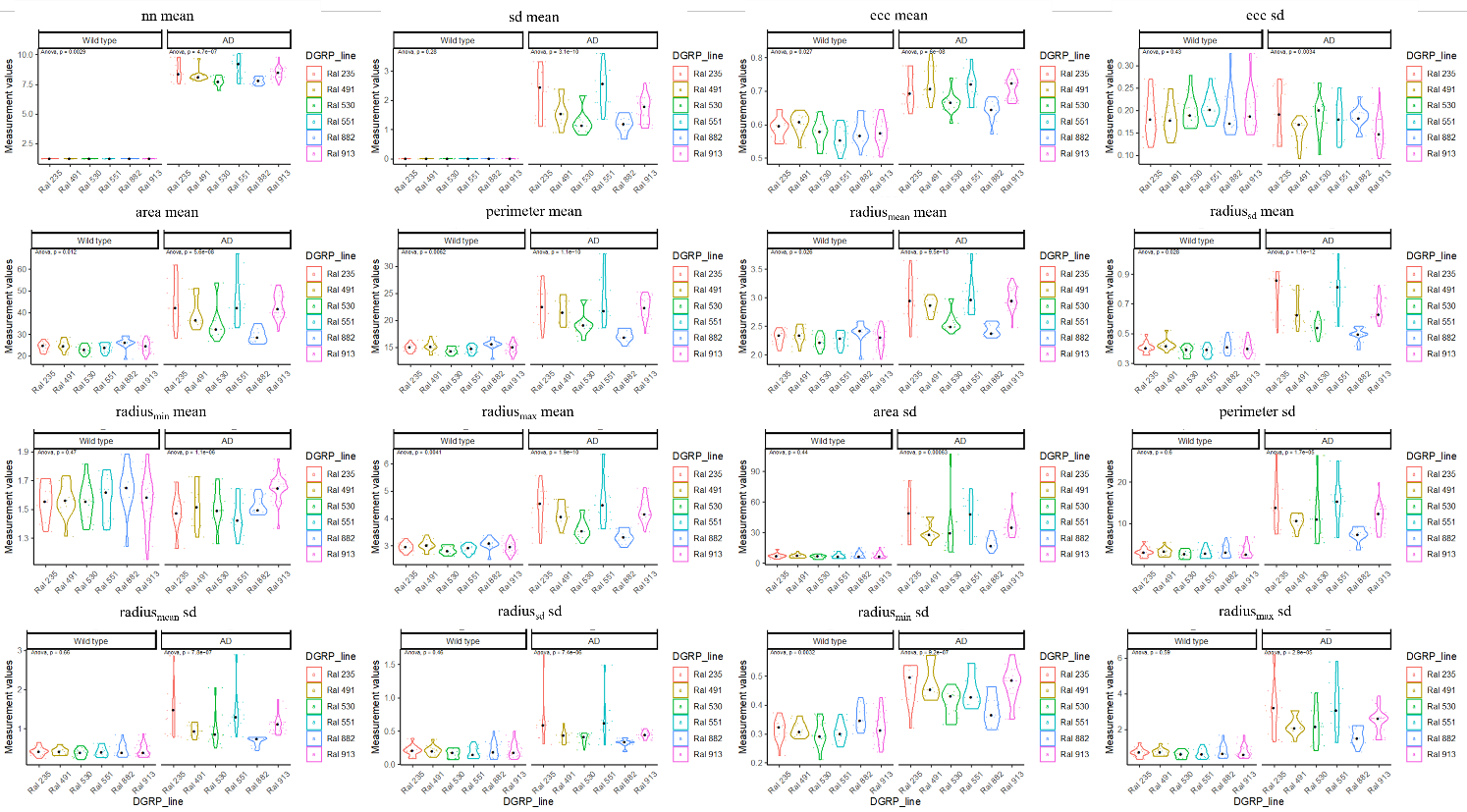


**Supplementary Figure S4. Phenotypic measurements reflect the toxicity induced by AB42 and Tau in different genetic backgrounds and are not an inherent property of DGRP strains.** Each panel figure shows one fly eye trait metric in six DGRP strains (right, denoted as Wild-type) and their respective Aβ42- and tau-expressing transgenic flies (left, denoted as AD). The larger black dot in each violin plot represents the median and the P value associated with each figure was computed via the ANOVA.
