# Supplementary material for: Using Drosophila to identify naturally occurring genetic modifiers of amyloid beta 42- and tau-induced toxicity
Source: G3 (Bethesda). 2023 Jun 13;13(9):jkad132. doi: 10.1093/g3journal/jkad132 (PMC10468303; doi:10.1093/g3journal/jkad132)
Supplement: jkad132_Supplementary_Data [file jkad132_supplementary_data.zip › Figure_S5_G3-2023-404168.docx]

**Figure S5
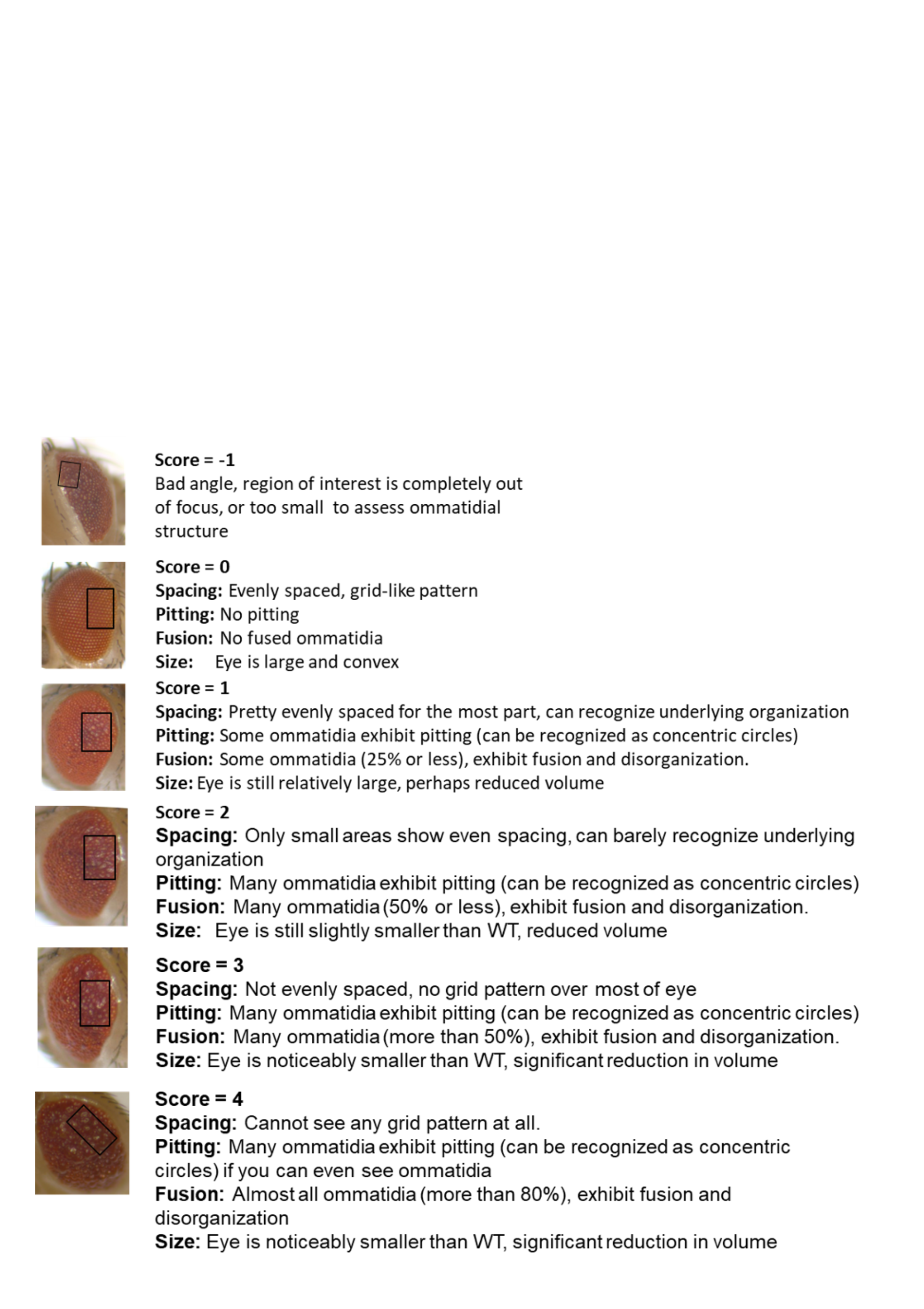
**

**Supplementary Figure S5. Rubric used for human scoring.** Rubric and representative eye images used to guide human analysis of eye degeneration. Briefly, humans blinded to genotype assigned a score based upon the above rubric for each of 200 images of fly eyes at varying degrees of degeneration. Scores of 0 correspond to a perfectly crystalline ommatidial structure while images receiving scores of -1 were censored.
