# Supplementary material for: Using Drosophila to identify naturally occurring genetic modifiers of amyloid beta 42- and tau-induced toxicity
Source: G3 (Bethesda). 2023 Jun 13;13(9):jkad132. doi: 10.1093/g3journal/jkad132 (PMC10468303; doi:10.1093/g3journal/jkad132)
Supplement: jkad132_Supplementary_Data [file jkad132_supplementary_data.zip › Figure_S6_G3-2023-404168.docx]

**Figure S6**

**
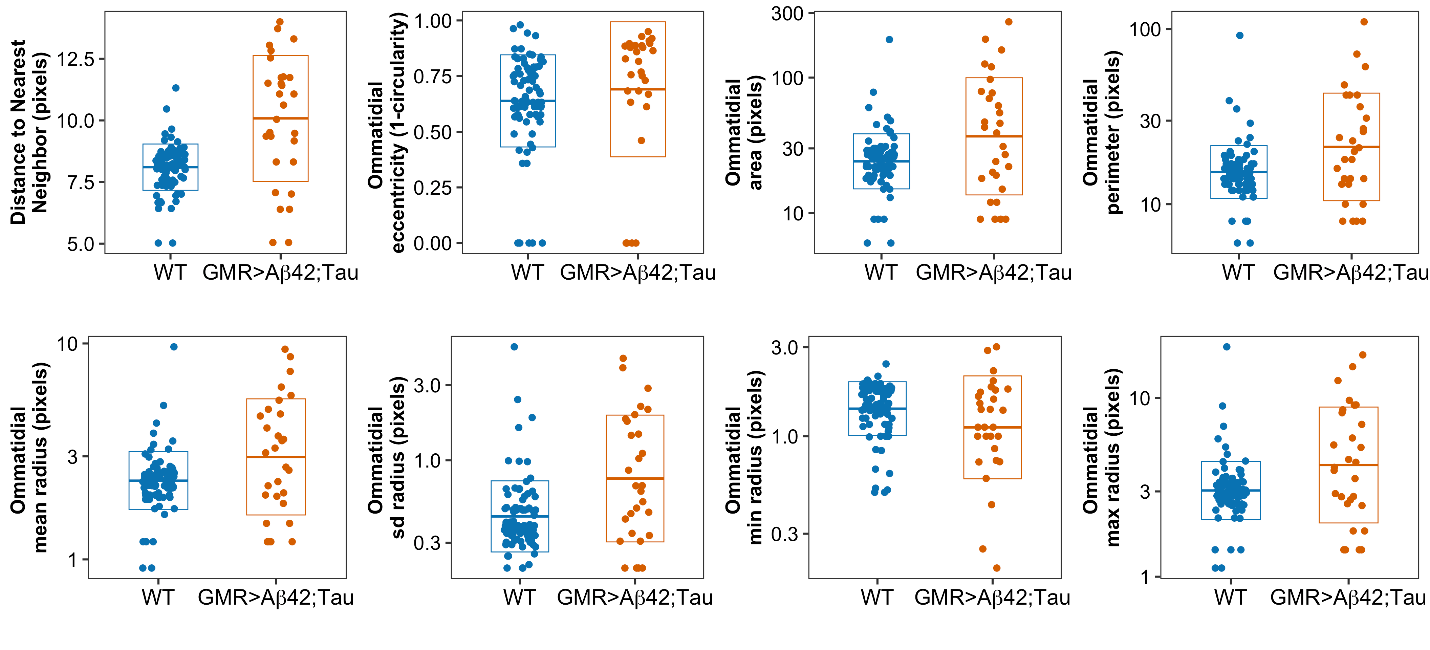
**

**Supplementary Figure S6. Expression of Aβ42 and tau in the fly eye leads to quantifiable morphological changes at the level of individual ommatidium.** Our analysis pipeline extracts five ommatidial measurements including ommatidial radius, perimeter, and circularity, as well as relative location. From these measurements, 14 features are calculated, including distance to the nearest neighbor, minimal and maximal radii, mean values, and standard deviation within an image and across replicates. Central measurements include nn mean, ecc mean, area mean, radius_mean_ mean and perimeter mean, and dispersion measurements include nn sd, ecc sd, area sd, radius_mean_ sd, perimeter sd, radius_max_ mean, radius_max_ sd, radius_min_ mean, radius_min_ sd
