# Supplementary material for: Using Drosophila to identify naturally occurring genetic modifiers of amyloid beta 42- and tau-induced toxicity
Source: G3 (Bethesda). 2023 Jun 13;13(9):jkad132. doi: 10.1093/g3journal/jkad132 (PMC10468303; doi:10.1093/g3journal/jkad132)
Supplement: jkad132_Supplementary_Data [file jkad132_supplementary_data.zip › Figure_S7_G3-2023-404168.docx]

**Figure S7**

**
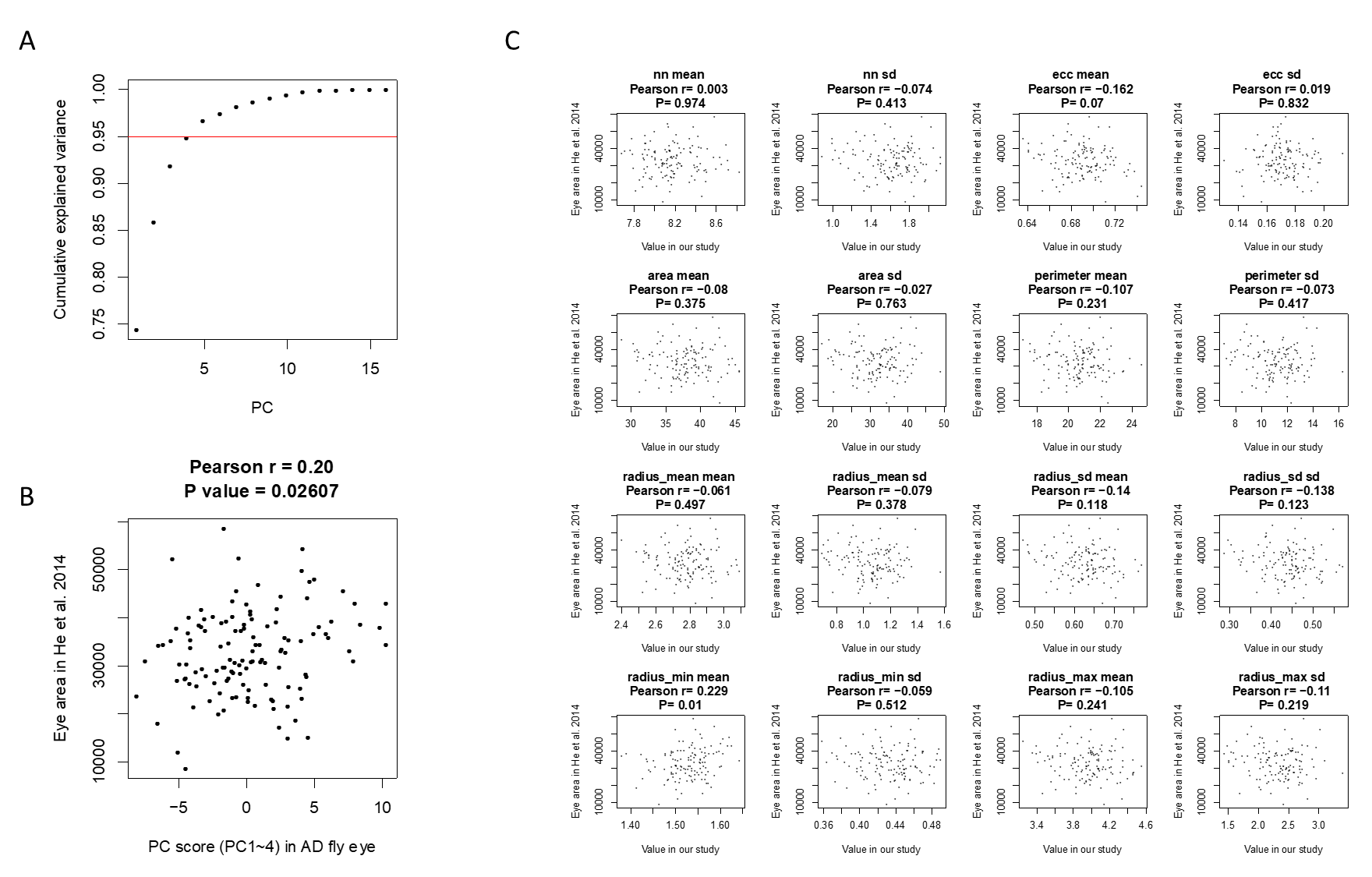
**

**Supplementary Figure S7. Correlation of mean eye area size measured in He et al. (2014) with ommatidial traits measured across DGRP lines.** The mean fly eye area size values in He et al. (2014) were extracted from Table S1 (He et al. 2014). Their study constructed transgenic flies through the expression of the mutant preproinsulin (hINSC96Y) in the fly eye imaginal disc in 178 DGRP fly lines, 126 out of which overlapped with our study. (A) Explained variance using PCA on the 16 fly eye ommatidial traits values used in our study. The red horizontal line indicated the 95% variance explained cutoff to prioritize PC1 to PC4. (B) The correlation between our fly eye score and the mean eye area size in He et al. (2014) for the 126 overlapped fly DGRP lines. The PC eye score on x-axis was computed as the sum of PC1 to PC4. Each dot indicates one DGRP line, as mean values across images per line. (C) The correlation between each individual fly eye metric used in our study and the mean eye area size in He et al. (2014) for the 126 overlapped fly DGRP lines. In each panel, each dot indicates one DGRP line, as mean values across images per line.
