# Supplementary material for: Using Drosophila to identify naturally occurring genetic modifiers of amyloid beta 42- and tau-induced toxicity
Source: G3 (Bethesda). 2023 Jun 13;13(9):jkad132. doi: 10.1093/g3journal/jkad132 (PMC10468303; doi:10.1093/g3journal/jkad132)
Supplement: jkad132_Supplementary_Data [file jkad132_supplementary_data.zip › Figure_S8_G3-2023-404168.docx]

**Figure S8**

**
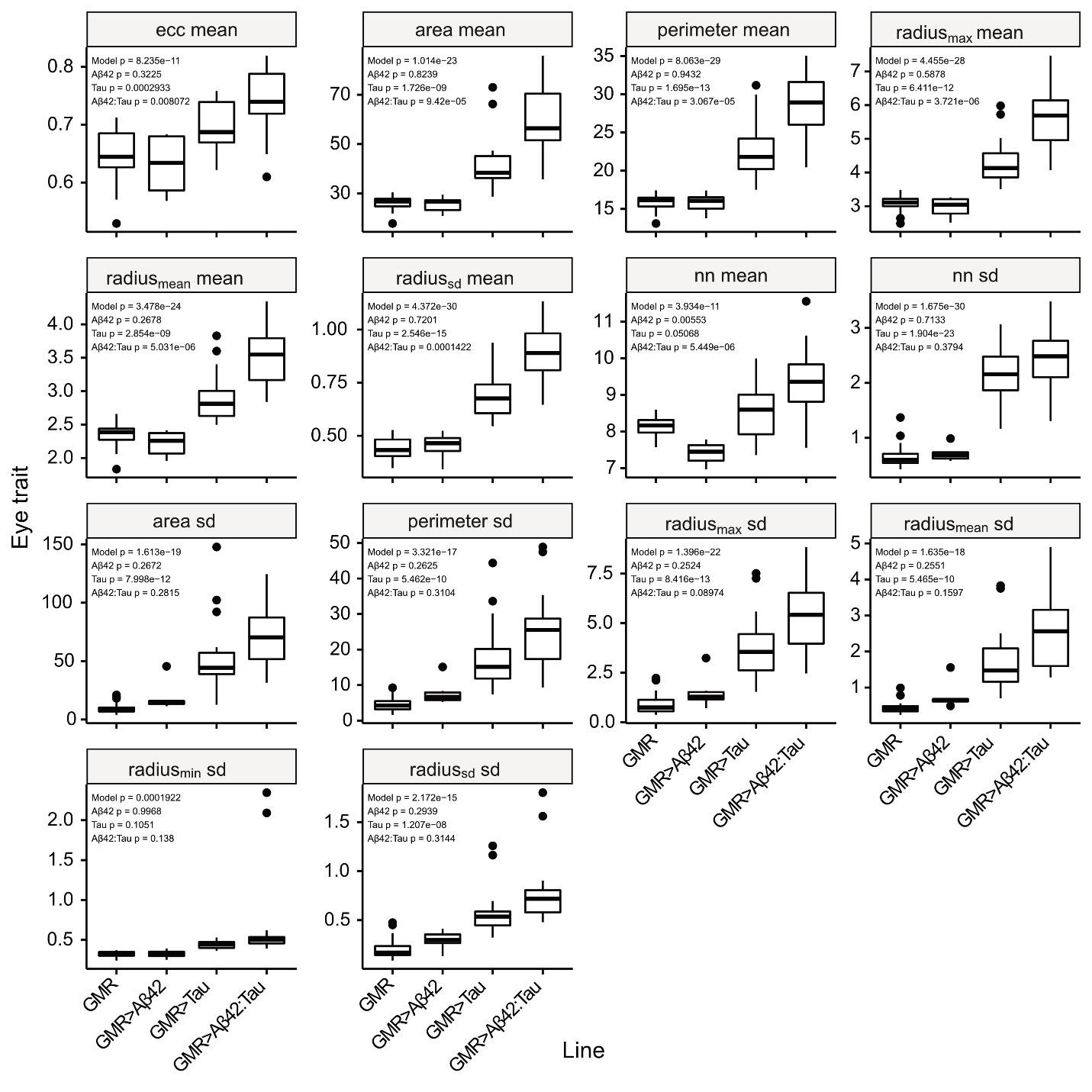
**

**Supplementary Figure S8. Expression of Ab42 exacerbates tau-induced degeneration across traits.** Quantification of rough eye phenotype across all 14 traits in flies expressing GMR-Gal4 alone, GMR-Gal4 and Ab42 (GMR>Ab42), GMR-Gal4 and UAS_tau (GMR>Tau), and GMR-Gal4, UAS_Ab42 and UAS_tau (GMR>Ab42;tau) reveals a significant effect of GMR-gal4 and Ab42 in 2 out of 14 traits, while expression of GMR-ga4 and Tau alone results in significant degeneration as measured in 12 out of 14 traits. A synergistic effect of Ab42 and tau in the fly eye is detected in all 14 traits based on a fixed-effect interaction model to quantify the individual additive effects of Aβ42 and tau, and the non-additive interaction between transgenes.
