# Supplementary material for: Using Drosophila to identify naturally occurring genetic modifiers of amyloid beta 42- and tau-induced toxicity
Source: G3 (Bethesda). 2023 Jun 13;13(9):jkad132. doi: 10.1093/g3journal/jkad132 (PMC10468303; doi:10.1093/g3journal/jkad132)
Supplement: jkad132_Supplementary_Data [file jkad132_supplementary_data.zip › Figure_S9_G3-2023-404168.docx]

**Figure S9**

**
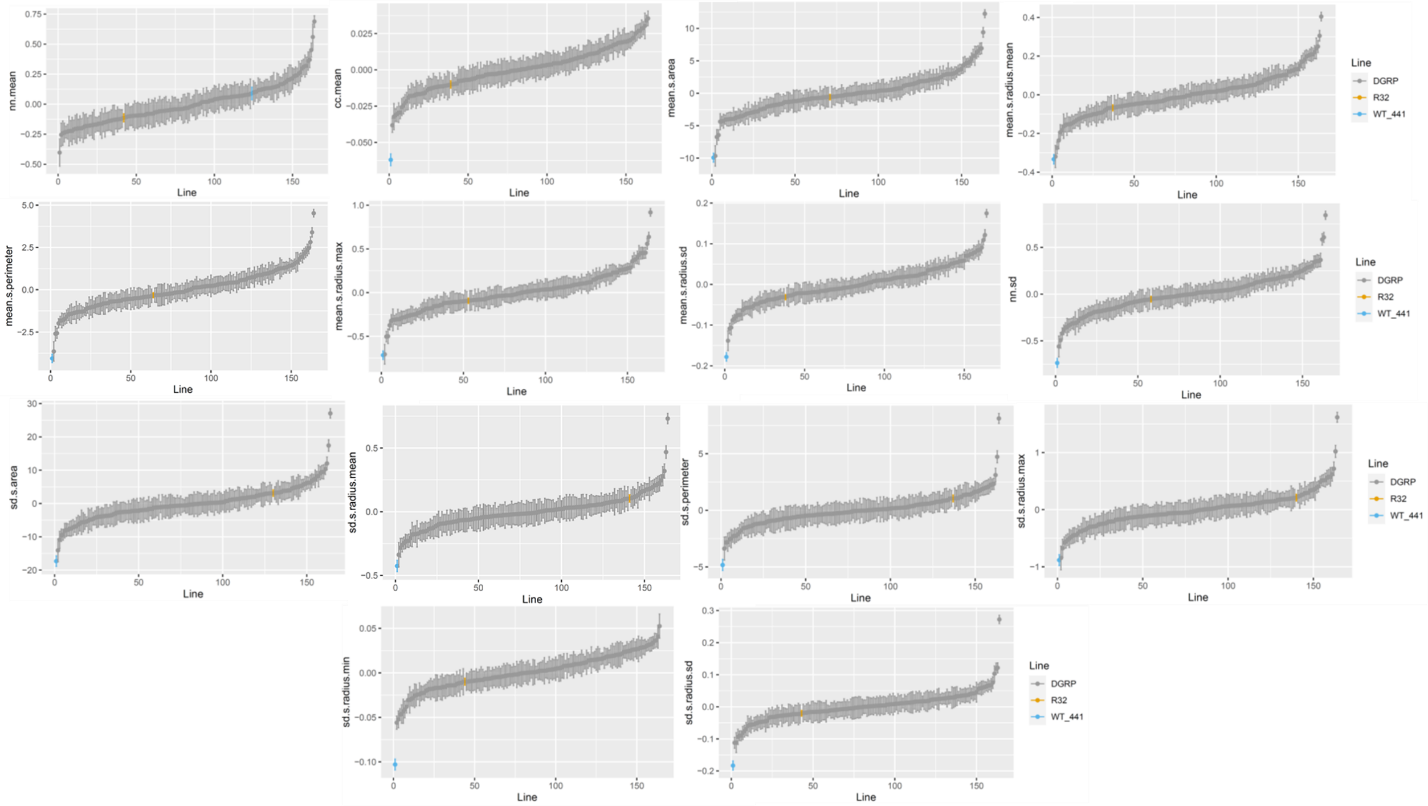
**

**Supplementary Figure S9. Genetic variation across all traits.** Rank-ordered BLUP scores for the 14 traits measured show variation in degree of degeneration across DGRP lines. Gray box and whisker plots are values derived from the BLUP within a line of the DGRP, yellow plot is the score for our triple transgenic donor line and the blue plot is a representative WT eye from a non-transgenic DGRP line 441.
