# Supplementary material for: Using Drosophila to identify naturally occurring genetic modifiers of amyloid beta 42- and tau-induced toxicity
Source: G3 (Bethesda). 2023 Jun 13;13(9):jkad132. doi: 10.1093/g3journal/jkad132 (PMC10468303; doi:10.1093/g3journal/jkad132)
Supplement: jkad132_Supplementary_Data [file jkad132_supplementary_data.zip › Table_S1_G3-2023-404168.docx]

**Supplementary Table S1**

| **Supplementary Table 1. Suggestive SNPs (*P* < 1x10^-5^) for each trait ranked by *P*-value.** | | | | | | | |
| --- | --- | --- | --- | --- | --- | --- | --- |
| SNP | *P* | *P*_adjust_ | Flybase ID1 | Flybase ID2 | Flybase ID3 | Flybase ID4 | Flybase ID5 |
| **nn mean** | | | | | | | |
| 2L_12758562_SNP | 1.4E-08 | 0.026549 | manualID: FBgn0032456 |  |  |  |  |
|  |  |  |  |  |  |  |  |
| X_14814258_SNP | 7.86E-08 | 0.055878 | FBgn0264078 | FBgn0030598 |  |  |  |
| 2R_20201951_SNP | 1.16E-07 | 0.055878 |  |  |  |  |  |
| 2L_9235056_SNP | 1.26E-07 | 0.055878 | FBgn0041092 |  |  |  |  |
| 3L_2992563_SNP | 1.5E-07 | 0.055878 |  |  |  |  |  |
| 2L_6792476_SNP | 1.77E-07 | 0.055878 | FBgn0015777 |  |  |  |  |
| 2L_9234952_DEL | 7.11E-07 | 0.165922 | FBgn0041092 |  |  |  |  |
| 2L_9234970_DEL | 7.11E-07 | 0.165922 | FBgn0041092 |  |  |  |  |
| 2L_17739964_SNP | 7.9E-07 | 0.165922 | FBgn0015609 |  |  |  |  |
| 3R_5213921_SNP | 1.1E-06 | 0.191332 | FBgn0000723 |  |  |  |  |
| X_11869322_SNP | 1.11E-06 | 0.191332 | FBgn0263111 |  |  |  |  |
| 2R_17334309_SNP | 1.6E-06 | 0.230775 | FBgn0010415 |  |  |  |  |
| 3L_937224_SNP | 1.74E-06 | 0.230775 | FBgn0264574 |  |  |  |  |
| 3L_9537168_SNP | 1.94E-06 | 0.230775 | FBgn0036010 |  |  |  |  |
| X_3990745_SNP | 2E-06 | 0.230775 | FBgn0029693 |  |  |  |  |
| 2L_6792635_SNP | 2.16E-06 | 0.230775 | FBgn0015777 |  |  |  |  |
| 2R_4982686_SNP | 2.25E-06 | 0.230775 | FBgn0082981 | FBgn0083124 | FBgn0033374 | FBgn0086672 | FBgn0082928 |
| 2R_20201950_SNP | 2.44E-06 | 0.230775 |  |  |  |  |  |
| 3L_937216_SNP | 2.82E-06 | 0.230775 | FBgn0264574 |  |  |  |  |
| 3L_8795173_SNP | 2.82E-06 | 0.230775 | FBgn0085455 | FBgn0035929 |  |  |  |
| 2L_9236598_SNP | 2.91E-06 | 0.230775 | FBgn0041092 | FBgn0051885 |  |  |  |
| 3L_8204998_SNP | 3.17E-06 | 0.230775 | FBgn0035857 |  |  |  |  |
| 2L_15972494_SNP | 3.26E-06 | 0.230775 |  |  |  |  |  |
| 3L_937348_SNP | 3.46E-06 | 0.230775 | FBgn0264574 |  |  |  |  |
| 2L_16367015_SNP | 3.52E-06 | 0.230775 | FBgn0028841 |  |  |  |  |
| 3L_13835541_SNP | 3.56E-06 | 0.230775 | FBgn0036380 | FBgn0040812 |  |  |  |
| 3L_8204999_SNP | 3.57E-06 | 0.230775 | FBgn0035857 |  |  |  |  |
| 3L_937304_DEL | 3.63E-06 | 0.230775 | FBgn0264574 |  |  |  |  |
| 2R_12224005_SNP | 3.64E-06 | 0.230775 | FBgn0262511 | FBgn0010611 |  |  |  |
| 3L_13845620_SNP | 4.03E-06 | 0.230775 | FBgn0029167 |  |  |  |  |
| 2L_9233022_SNP | 4.29E-06 | 0.230775 | FBgn0041092 |  |  |  |  |
| 2R_17061349_SNP | 4.44E-06 | 0.230775 | FBgn0261850 |  |  |  |  |
| 2L_1904738_SNP | 4.54E-06 | 0.230775 | FBgn0031374 |  |  |  |  |
| 2L_6792661_SNP | 4.62E-06 | 0.230775 | FBgn0015777 |  |  |  |  |
| 2R_14832723_SNP | 4.65E-06 | 0.230775 | FBgn0034408 |  |  |  |  |
| 2L_9233934_SNP | 5.03E-06 | 0.230775 | FBgn0041092 |  |  |  |  |
| 3R_8960011_SNP | 5.12E-06 | 0.230775 | FBgn0266578 | FBgn0038118 | FBgn0260793 |  |  |
| 3L_13836564_SNP | 5.34E-06 | 0.230775 | FBgn0040812 | FBgn0043550 |  |  |  |
| X_15179092_SNP | 5.38E-06 | 0.230775 |  |  |  |  |  |
| 2L_9237343_SNP | 5.51E-06 | 0.230775 | FBgn0041092 | FBgn0051885 |  |  |  |
| 2L_9236502_SNP | 5.58E-06 | 0.230775 | FBgn0041092 | FBgn0051885 |  |  |  |
| 2L_9236506_SNP | 5.58E-06 | 0.230775 | FBgn0041092 | FBgn0051885 |  |  |  |
| 2L_9236488_DEL | 5.58E-06 | 0.230775 | FBgn0051885 | FBgn0041092 |  |  |  |
| 2L_16407492_SNP | 5.59E-06 | 0.230775 | FBgn0028523 |  |  |  |  |
| 2R_11069546_SNP | 5.67E-06 | 0.230775 | FBgn0013467 |  |  |  |  |
| 3L_13853337_SNP | 5.87E-06 | 0.230775 | FBgn0029167 | FBgn0036381 |  |  |  |
| 2R_14722590_SNP | 5.89E-06 | 0.230775 | FBgn0034398 | FBgn0034399 |  |  |  |
| 2L_9233896_SNP | 5.95E-06 | 0.230775 | FBgn0041092 |  |  |  |  |
| 2L_16409030_SNP | 5.98E-06 | 0.230775 | FBgn0028523 |  |  |  |  |
| 2L_13738864_SNP | 6.14E-06 | 0.232324 |  |  |  |  |  |
| 2L_16653424_SNP | 6.35E-06 | 0.235259 | FBgn0259735 |  |  |  |  |
| 2R_14722541_SNP | 6.71E-06 | 0.244153 | FBgn0034398 | FBgn0034399 |  |  |  |
| 2L_9236507_SNP | 7.23E-06 | 0.257992 | FBgn0041092 | FBgn0051885 |  |  |  |
| 2R_4969547_SNP | 7.47E-06 | 0.261549 | FBgn0033371 | FBgn0025709 |  |  |  |
| 3L_13845621_SNP | 7.75E-06 | 0.266317 | FBgn0029167 |  |  |  |  |
| 2L_7914671_SNP | 8.16E-06 | 0.275642 | FBgn0085450 |  |  |  |  |
| 3L_13845635_SNP | 8.99E-06 | 0.292042 | FBgn0029167 |  |  |  |  |
| 2L_9233980_SNP | 9.08E-06 | 0.292042 | FBgn0041092 |  |  |  |  |
| 2R_2645497_SNP | 9.11E-06 | 0.292042 | FBgn0033101 | FBgn0033100 |  |  |  |
| 2R_10290277_SNP | 9.4E-06 | 0.296157 |  |  |  |  |  |
| 2L_16305028_SNP | 9.72E-06 | 0.297766 | FBgn0028525 | FBgn0015338 |  |  |  |
| 2L_6855504_SNP | 9.76E-06 | 0.297766 | FBgn0086451 |  |  |  |  |
| SNP | *P* | *P*_adjust_ | Flybase ID1 | Flybase ID2 | Flybase ID3 | Flybase ID4 | Flybase ID5 |
| **ecc mean** | | | | | | | |
| 3L_4169461_SNP | 1.11E-06 | 0.994071 | FBgn0261274 | FBgn0265722 |  |  |  |
| 2L_11329080_SNP | 1.23E-06 | 0.994071 |  |  |  |  |  |
| X_11276301_SNP | 2.64E-06 | 0.994071 | FBgn0001624 |  |  |  |  |
| X_11276302_SNP | 2.65E-06 | 0.994071 | FBgn0001624 |  |  |  |  |
| X_6434631_SNP | 2.88E-06 | 0.994071 | FBgn0085446 |  |  |  |  |
| X_4359575_SNP | 3.88E-06 | 0.994071 | FBgn0052773 | FBgn0000179 |  |  |  |
| 2R_1564800_SNP | 5.6E-06 | 0.994071 | FBgn0033033 |  |  |  |  |
| X_6434627_SNP | 5.67E-06 | 0.994071 | FBgn0085446 |  |  |  |  |
| 2L_15427353_SNP | 7.39E-06 | 0.994071 |  |  |  |  |  |
| X_6434608_SNP | 7.86E-06 | 0.994071 | FBgn0085446 |  |  |  |  |
| 2R_1501145_SNP | 8.03E-06 | 0.994071 |  |  |  |  |  |
| 2R_3264241_SNP | 8.24E-06 | 0.994071 | FBgn0033159 |  |  |  |  |
| 3L_4735932_SNP | 9.87E-06 | 0.994071 | FBgn0035574 |  |  |  |  |
| 2R_18613061_SNP | 9.95E-06 | 0.994071 | FBgn0028371 |  |  |  |  |
| SNP | *P* | *P*_adjust_ | Flybase ID1 | Flybase ID2 | Flybase ID3 | Flybase ID4 | Flybase ID5 |
| **area mean** | | | | | | | |
| X_4359575_SNP | 2.24E-07 | 0.278256 | FBgn0052773 | FBgn0000179 |  |  |  |
| 3L_937224_SNP | 4.28E-07 | 0.278256 | FBgn0264574 |  |  |  |  |
| 2L_2924056_SNP | 6.01E-07 | 0.278256 | FBgn0041111 |  |  |  |  |
| 2R_20201951_SNP | 7.21E-07 | 0.278256 |  |  |  |  |  |
| 2L_9235056_SNP | 8.74E-07 | 0.278256 | FBgn0041092 |  |  |  |  |
| 2L_12758562_SNP | 8.83E-07 | 0.278256 |  |  |  |  |  |
| 2L_2924011_SNP | 1.41E-06 | 0.297913 | FBgn0041111 |  |  |  |  |
| 2L_16046650_SNP | 1.62E-06 | 0.297913 | FBgn0000182 | FBgn0266459 |  |  |  |
| X_4359702_SNP | 1.73E-06 | 0.297913 | FBgn0000179 | FBgn0052773 |  |  |  |
| 2L_12632220_SNP | 1.73E-06 | 0.297913 | FBgn0032439 |  |  |  |  |
| 2L_12632223_SNP | 1.73E-06 | 0.297913 | FBgn0032439 |  |  |  |  |
| 3L_937216_SNP | 1.92E-06 | 0.302082 | FBgn0264574 |  |  |  |  |
| X_4361594_SNP | 2.37E-06 | 0.325246 | FBgn0052773 | FBgn0000179 |  |  |  |
| 2L_16043209_SNP | 2.41E-06 | 0.325246 | FBgn0266459 | FBgn0000182 |  |  |  |
| 3L_8204998_SNP | 2.75E-06 | 0.3409 | FBgn0035857 |  |  |  |  |
| 3L_8204999_SNP | 3.13E-06 | 0.3409 | FBgn0035857 |  |  |  |  |
| 2L_6792476_SNP | 3.17E-06 | 0.3409 | FBgn0015777 |  |  |  |  |
| 3L_13835541_SNP | 3.25E-06 | 0.3409 | FBgn0036380 | FBgn0040812 |  |  |  |
| 3L_1578538_SNP | 3.76E-06 | 0.374511 | FBgn0035237 |  |  |  |  |
| 2L_9234952_DEL | 4.3E-06 | 0.374755 | FBgn0041092 |  |  |  |  |
| 2L_9234970_DEL | 4.3E-06 | 0.374755 | FBgn0041092 |  |  |  |  |
| 3L_20562243_SNP | 4.36E-06 | 0.374755 |  |  |  |  |  |
| X_11869322_SNP | 5.11E-06 | 0.420288 | FBgn0263111 |  |  |  |  |
| 2L_16367015_SNP | 5.88E-06 | 0.452087 | FBgn0028841 |  |  |  |  |
| 2R_14722541_SNP | 6.12E-06 | 0.452087 | FBgn0034398 | FBgn0034399 |  |  |  |
| 3L_937304_DEL | 6.22E-06 | 0.452087 | FBgn0264574 |  |  |  |  |
| 3R_16318170_SNP | 6.51E-06 | 0.455723 |  |  |  |  |  |
| 3L_8030270_SNP | 7.33E-06 | 0.479549 | FBgn0011817 |  |  |  |  |
| 2R_9040903_INS | 7.6E-06 | 0.479549 | FBgn0263998 | FBgn0020370 |  |  |  |
| 3L_937348_SNP | 7.61E-06 | 0.479549 | FBgn0264574 |  |  |  |  |
| 2L_4294028_SNP | 8.62E-06 | 0.510561 | FBgn0010473 |  |  |  |  |
| 2L_16054772_SNP | 9.5E-06 | 0.510561 | FBgn0013433 |  |  |  |  |
| 2R_14722590_SNP | 9.84E-06 | 0.510561 | FBgn0034398 | FBgn0034399 |  |  |  |
| 3L_2992563_SNP | 9.86E-06 | 0.510561 |  |  |  |  |  |
| X_11193212_DEL | 9.9E-06 | 0.510561 | FBgn0265595 |  |  |  |  |
| SNP | *P* | *P*_adjust_ | Flybase ID1 | Flybase ID2 | Flybase ID3 | Flybase ID4 | Flybase ID5 |
| **Radius_mean_ mean** | | | | | | | |
| 2L_9235056_SNP | 1.14E-07 | 0.147306 | FBgn0041092 |  |  |  |  |
| 3L_937224_SNP | 1.65E-07 | 0.147306 | FBgn0264574 |  |  |  |  |
| 2L_12758562_SNP | 2.34E-07 | 0.147306 |  |  |  |  |  |
| 3L_3114013_SNP | 5.85E-07 | 0.230273 | FBgn0026189 | FBgn0266437 |  |  |  |
| 2R_20201951_SNP | 7.33E-07 | 0.230273 |  |  |  |  |  |
| 3L_937216_SNP | 9.7E-07 | 0.230273 | FBgn0264574 |  |  |  |  |
| 2L_9234952_DEL | 9.74E-07 | 0.230273 | FBgn0041092 |  |  |  |  |
| 2L_9234970_DEL | 9.74E-07 | 0.230273 | FBgn0041092 |  |  |  |  |
| 3R_5213921_SNP | 1.17E-06 | 0.235236 | FBgn0000723 |  |  |  |  |
| 2L_6792476_SNP | 1.24E-06 | 0.235236 | FBgn0015777 |  |  |  |  |
| 3L_13835541_SNP | 2.54E-06 | 0.437157 | FBgn0036380 | FBgn0040812 |  |  |  |
| 2R_3250007_SNP | 3.37E-06 | 0.48787 | FBgn0033159 |  |  |  |  |
| 2R_12224005_SNP | 3.44E-06 | 0.48787 | FBgn0262511 | FBgn0010611 |  |  |  |
| X_636240_DEL | 3.93E-06 | 0.48787 | FBgn0021764 |  |  |  |  |
| X_11869322_SNP | 4.23E-06 | 0.48787 | FBgn0263111 |  |  |  |  |
| 3L_20562243_SNP | 4.35E-06 | 0.48787 |  |  |  |  |  |
| X_14814258_SNP | 4.62E-06 | 0.48787 | FBgn0264078 | FBgn0030598 |  |  |  |
| X_4361594_SNP | 4.64E-06 | 0.48787 | FBgn0052773 | FBgn0000179 |  |  |  |
| 3L_937304_DEL | 5.17E-06 | 0.514244 | FBgn0264574 |  |  |  |  |
| 2R_11069546_SNP | 6.06E-06 | 0.544672 | FBgn0013467 |  |  |  |  |
| 3L_937348_SNP | 6.43E-06 | 0.544672 | FBgn0264574 |  |  |  |  |
| 2R_20201950_SNP | 6.51E-06 | 0.544672 |  |  |  |  |  |
| X_11276301_SNP | 7.29E-06 | 0.544672 | FBgn0001624 |  |  |  |  |
| 2L_16046650_SNP | 7.33E-06 | 0.544672 | FBgn0000182 | FBgn0266459 |  |  |  |
| 2L_16043209_SNP | 7.37E-06 | 0.544672 | FBgn0266459 | FBgn0000182 |  |  |  |
| X_4359575_SNP | 7.49E-06 | 0.544672 | FBgn0052773 | FBgn0000179 |  |  |  |
| X_4359702_SNP | 8.83E-06 | 0.587809 | FBgn0000179 | FBgn0052773 |  |  |  |
| 2R_12601327_SNP | 9.03E-06 | 0.587809 | FBgn0050463 |  |  |  |  |
| 3L_2992563_SNP | 9.05E-06 | 0.587809 |  |  |  |  |  |
| X_11276302_SNP | 9.58E-06 | 0.587809 | FBgn0001624 |  |  |  |  |
| SNP | *P* | *P*_adjust_ | Flybase ID1 | Flybase ID2 | Flybase ID3 | Flybase ID4 | Flybase ID5 |
| **Perimeter mean** | | | | | | | |
| X_4359575_SNP | 1.63E-07 | 0.308984 | FBgn0052773 | FBgn0000179 |  |  |  |
| 3L_937224_SNP | 4.44E-07 | 0.32241 | FBgn0264574 |  |  |  |  |
| 2L_16046650_SNP | 6.44E-07 | 0.32241 | FBgn0000182 | FBgn0266459 |  |  |  |
| 2L_16043209_SNP | 6.82E-07 | 0.32241 | FBgn0266459 | FBgn0000182 |  |  |  |
| X_4359702_SNP | 1.2E-06 | 0.419615 | FBgn0000179 | FBgn0052773 |  |  |  |
| 2L_9235056_SNP | 1.5E-06 | 0.419615 | FBgn0041092 |  |  |  |  |
| 2R_20201951_SNP | 1.66E-06 | 0.419615 |  |  |  |  |  |
| 2L_16042873_SNP | 0.000002 | 0.419615 | FBgn0000182 | FBgn0266459 |  |  |  |
| 2R_18613061_SNP | 2.2E-06 | 0.419615 | FBgn0028371 |  |  |  |  |
| 2L_12758562_SNP | 2.44E-06 | 0.419615 |  |  |  |  |  |
| 3L_937216_SNP | 2.64E-06 | 0.419615 | FBgn0264574 |  |  |  |  |
| X_4361594_SNP | 2.75E-06 | 0.419615 | FBgn0052773 | FBgn0000179 |  |  |  |
| 2L_2924056_SNP | 3.02E-06 | 0.419615 | FBgn0041111 |  |  |  |  |
| 3L_8030270_SNP | 3.17E-06 | 0.419615 | FBgn0011817 |  |  |  |  |
| 3L_1578538_SNP | 3.33E-06 | 0.419615 | FBgn0035237 |  |  |  |  |
| 2L_4294028_SNP | 3.64E-06 | 0.419615 | FBgn0010473 |  |  |  |  |
| 3L_20562243_SNP | 3.98E-06 | 0.419615 |  |  |  |  |  |
| 2L_16054772_SNP | 4.07E-06 | 0.419615 | FBgn0013433 |  |  |  |  |
| 3R_5213921_SNP | 4.51E-06 | 0.419615 | FBgn0000723 |  |  |  |  |
| 2L_11426355_SNP | 4.55E-06 | 0.419615 |  |  |  |  |  |
| 2L_2924011_SNP | 4.66E-06 | 0.419615 | FBgn0041111 |  |  |  |  |
| 2L_16058280_SNP | 5.11E-06 | 0.428493 | FBgn0013433 |  |  |  |  |
| 2R_16322804_SNP | 5.23E-06 | 0.428493 | FBgn0086604 |  |  |  |  |
| 3L_14200065_SNP | 5.56E-06 | 0.428493 | FBgn0013718 |  |  |  |  |
| X_15419876_SNP | 5.67E-06 | 0.428493 | FBgn0022710 |  |  |  |  |
| 2L_6792476_SNP | 8.23E-06 | 0.560646 | FBgn0015777 |  |  |  |  |
| 3L_3114013_SNP | 8.4E-06 | 0.560646 | FBgn0026189 | FBgn0266437 |  |  |  |
| 3L_8204998_SNP | 8.63E-06 | 0.560646 | FBgn0035857 |  |  |  |  |
| 2L_16054897_SNP | 9.11E-06 | 0.560646 | FBgn0013433 |  |  |  |  |
| X_11846956_SNP | 9.31E-06 | 0.560646 | FBgn0263111 |  |  |  |  |
| 3L_8204999_SNP | 9.78E-06 | 0.560646 | FBgn0035857 |  |  |  |  |
| SNP | *P* | *P*_adjust_ | Flybase ID1 | Flybase ID2 | Flybase ID3 | Flybase ID4 | Flybase ID5 |
| **radius_max_ mean** | | | | | | | |
| 2L_9235056_SNP | 8.44E-08 | 0.159654 | FBgn0041092 |  |  |  |  |
| 3L_937224_SNP | 4.26E-07 | 0.232873 | FBgn0264574 |  |  |  |  |
| 2L_12758562_SNP | 5.76E-07 | 0.232873 |  |  |  |  |  |
| 2L_9234952_DEL | 7.06E-07 | 0.232873 | FBgn0041092 |  |  |  |  |
| 2L_9234970_DEL | 7.06E-07 | 0.232873 | FBgn0041092 |  |  |  |  |
| X_4359575_SNP | 7.39E-07 | 0.232873 | FBgn0052773 | FBgn0000179 |  |  |  |
| X_4359702_SNP | 1.74E-06 | 0.447723 | FBgn0000179 | FBgn0052773 |  |  |  |
| 2R_20201951_SNP | 2.34E-06 | 0.447723 |  |  |  |  |  |
| 3L_937216_SNP | 2.44E-06 | 0.447723 | FBgn0264574 |  |  |  |  |
| 3L_8204998_SNP | 2.7E-06 | 0.447723 | FBgn0035857 |  |  |  |  |
| 2R_12224005_SNP | 2.8E-06 | 0.447723 | FBgn0262511 | FBgn0010611 |  |  |  |
| X_4361594_SNP | 2.92E-06 | 0.447723 | FBgn0052773 | FBgn0000179 |  |  |  |
| 3L_8204999_SNP | 3.08E-06 | 0.447723 | FBgn0035857 |  |  |  |  |
| 3L_13835541_SNP | 3.82E-06 | 0.502336 | FBgn0036380 | FBgn0040812 |  |  |  |
| 2L_6792476_SNP | 4.17E-06 | 0.502336 | FBgn0015777 |  |  |  |  |
| 2L_11426355_SNP | 4.34E-06 | 0.502336 |  |  |  |  |  |
| 3R_5213921_SNP | 4.98E-06 | 0.502336 | FBgn0000723 |  |  |  |  |
| 3L_20562243_SNP | 5.1E-06 | 0.502336 |  |  |  |  |  |
| 2L_9234342_SNP | 5.27E-06 | 0.502336 | FBgn0041092 |  |  |  |  |
| 2R_18613061_SNP | 5.31E-06 | 0.502336 | FBgn0028371 |  |  |  |  |
| 2L_4294028_SNP | 5.74E-06 | 0.503427 | FBgn0010473 |  |  |  |  |
| 2R_17336952_INS | 5.86E-06 | 0.503427 | FBgn0010415 |  |  |  |  |
| X_11276301_SNP | 6.38E-06 | 0.521828 | FBgn0001624 |  |  |  |  |
| 2L_9233022_SNP | 6.62E-06 | 0.521828 | FBgn0041092 |  |  |  |  |
| X_14814258_SNP | 7.03E-06 | 0.53174 | FBgn0264078 | FBgn0030598 |  |  |  |
| 3L_3114013_SNP | 7.35E-06 | 0.534271 | FBgn0026189 | FBgn0266437 |  |  |  |
| 2R_11069546_SNP | 8.46E-06 | 0.546182 | FBgn0013467 |  |  |  |  |
| X_11276302_SNP | 8.64E-06 | 0.546182 | FBgn0001624 |  |  |  |  |
| 2L_4878745_SNP | 8.87E-06 | 0.546182 | FBgn0000286 |  |  |  |  |
| 2R_3250007_SNP | 8.91E-06 | 0.546182 | FBgn0033159 |  |  |  |  |
| 3L_1578538_SNP | 9.56E-06 | 0.546182 | FBgn0035237 |  |  |  |  |
| 3L_937304_DEL | 9.87E-06 | 0.546182 | FBgn0264574 |  |  |  |  |
| 2L_9233980_SNP | 9.98E-06 | 0.546182 | FBgn0041092 |  |  |  |  |
| SNP | *P* | *P*_adjust_ | Flybase ID1 | Flybase ID2 | Flybase ID3 | Flybase ID4 | Flybase ID5 |
| **radius_sd_ mean** | | | | | | | |
| 2L_9235056_SNP | 6.94E-07 | 0.683529 | FBgn0041092 |  |  |  |  |
| X_4359575_SNP | 1.15E-06 | 0.683529 | FBgn0052773 | FBgn0000179 |  |  |  |
| 2L_11426355_SNP | 0.000002 | 0.683529 |  |  |  |  |  |
| X_11276301_SNP | 2.47E-06 | 0.683529 | FBgn0001624 |  |  |  |  |
| 3L_4169461_SNP | 2.8E-06 | 0.683529 | FBgn0261274 | FBgn0265722 |  |  |  |
| X_11276302_SNP | 2.99E-06 | 0.683529 | FBgn0001624 |  |  |  |  |
| 3L_937224_SNP | 3.16E-06 | 0.683529 | FBgn0264574 |  |  |  |  |
| 2L_9234952_DEL | 3.26E-06 | 0.683529 | FBgn0041092 |  |  |  |  |
| 2L_9234970_DEL | 3.26E-06 | 0.683529 | FBgn0041092 |  |  |  |  |
| X_15419876_SNP | 5.79E-06 | 0.683529 | FBgn0022710 |  |  |  |  |
| X_4359702_SNP | 5.91E-06 | 0.683529 | FBgn0000179 | FBgn0052773 |  |  |  |
| 3L_8204998_SNP | 6.05E-06 | 0.683529 | FBgn0035857 |  |  |  |  |
| 2R_18613061_SNP | 6.83E-06 | 0.683529 | FBgn0028371 |  |  |  |  |
| 3L_8204999_SNP | 6.87E-06 | 0.683529 | FBgn0035857 |  |  |  |  |
| 2R_20201951_SNP | 7.53E-06 | 0.683529 |  |  |  |  |  |
| X_4361594_SNP | 7.61E-06 | 0.683529 | FBgn0052773 | FBgn0000179 |  |  |  |
| 2L_12758562_SNP | 7.63E-06 | 0.683529 |  |  |  |  |  |
| 2R_4969547_SNP | 8.48E-06 | 0.683529 | FBgn0033371 | FBgn0025709 |  |  |  |
| 2L_11426419_SNP | 8.62E-06 | 0.683529 |  |  |  |  |  |
| 3L_937216_SNP | 8.95E-06 | 0.683529 | FBgn0264574 |  |  |  |  |
| 2R_3250007_SNP | 9.14E-06 | 0.683529 | FBgn0033159 |  |  |  |  |
| 2L_12826039_INS | 9.2E-06 | 0.683529 |  |  |  |  |  |
| 3L_4735932_SNP | 9.31E-06 | 0.683529 | FBgn0035574 |  |  |  |  |
| 2L_11426428_SNP | 9.46E-06 | 0.683529 |  |  |  |  |  |
| 2L_11426418_SNP | 9.72E-06 | 0.683529 |  |  |  |  |  |
| 2R_12601327_SNP | 9.95E-06 | 0.683529 | FBgn0050463 |  |  |  |  |
| SNP | *P* | *P*_adjust_ | Flybase ID1 | Flybase ID2 | Flybase ID3 | Flybase ID4 | Flybase ID5 |
| **nn sd** | | | | | | | |
| 2L_4294028_SNP | 5.21E-08 | 0.098557 | FBgn0010473 |  |  |  |  |
| 2L_4294043_DEL | 3.64E-07 | 0.344251 | FBgn0010473 |  |  |  |  |
| 2L_4294050_SNP | 6.31E-07 | 0.397797 | FBgn0010473 |  |  |  |  |
| 2L_4294053_SNP | 8.95E-07 | 0.423293 | FBgn0010473 |  |  |  |  |
| 2L_9235056_SNP | 2.06E-06 | 0.542944 | FBgn0041092 |  |  |  |  |
| 2L_7225324_SNP | 2.97E-06 | 0.542944 | FBgn0259111 |  |  |  |  |
| 3L_2692607_SNP | 2.99E-06 | 0.542944 | FBgn0264606 |  |  |  |  |
| 2R_16521194_SNP | 3.42E-06 | 0.542944 | FBgn0250850 | FBgn0034530 |  |  |  |
| 3R_6268403_SNP | 3.66E-06 | 0.542944 |  |  |  |  |  |
| X_15419876_SNP | 3.7E-06 | 0.542944 | FBgn0022710 |  |  |  |  |
| 3L_1578538_SNP | 3.93E-06 | 0.542944 | FBgn0035237 |  |  |  |  |
| 2L_4294037_SNP | 4.09E-06 | 0.542944 | FBgn0010473 |  |  |  |  |
| 2R_1716201_SNP | 4.14E-06 | 0.542944 | FBgn0085414 |  |  |  |  |
| 3L_5460050_SNP | 4.33E-06 | 0.542944 |  |  |  |  |  |
| X_4361594_SNP | 4.4E-06 | 0.542944 | FBgn0052773 | FBgn0000179 |  |  |  |
| 2L_16292838_SNP | 4.59E-06 | 0.542944 | FBgn0032582 | FBgn0266204 | FBgn0028896 |  |  |
| 2R_1731235_SNP | 5.18E-06 | 0.561922 | FBgn0085414 |  |  |  |  |
| X_14281613_SNP | 5.77E-06 | 0.561922 | FBgn0052600 |  |  |  |  |
| 3L_5460054_SNP | 5.92E-06 | 0.561922 |  |  |  |  |  |
| X_4359575_SNP | 6.31E-06 | 0.561922 | FBgn0052773 | FBgn0000179 |  |  |  |
| 3L_20562243_SNP | 6.51E-06 | 0.561922 |  |  |  |  |  |
| 2L_16042873_SNP | 6.69E-06 | 0.561922 | FBgn0000182 | FBgn0266459 |  |  |  |
| 2L_16043209_SNP | 7.44E-06 | 0.561922 | FBgn0266459 | FBgn0000182 |  |  |  |
| 3L_5460128_SNP | 7.93E-06 | 0.561922 |  |  |  |  |  |
| 3L_8204998_SNP | 8.12E-06 | 0.561922 | FBgn0035857 |  |  |  |  |
| 2R_13705136_SNP | 8.28E-06 | 0.561922 | FBgn0259211 |  |  |  |  |
| 2L_8915684_SNP | 8.43E-06 | 0.561922 | FBgn0085427 |  |  |  |  |
| 3R_1685103_SNP | 8.65E-06 | 0.561922 | FBgn0037391 |  |  |  |  |
| 3L_10518320_SNP | 8.85E-06 | 0.561922 | FBgn0052062 |  |  |  |  |
| 2L_8682092_SNP | 9.1E-06 | 0.561922 | FBgn0032058 |  |  |  |  |
| 3L_8204999_SNP | 9.21E-06 | 0.561922 | FBgn0035857 |  |  |  |  |
| SNP | *P* | *P*_adjust_ | Flybase ID1 | Flybase ID2 | Flybase ID3 | Flybase ID4 | Flybase ID5 |
| **area sd** | | | | | | | |
| X_4359575_SNP | 6.07E-08 | 0.076269 | FBgn0052773 | FBgn0000179 |  |  |  |
| X_4361594_SNP | 8.88E-08 | 0.076269 | FBgn0052773 | FBgn0000179 |  |  |  |
| X_4359702_SNP | 1.21E-07 | 0.076269 | FBgn0000179 | FBgn0052773 |  |  |  |
| 2R_16322804_SNP | 2.12E-07 | 0.083266 | FBgn0086604 |  |  |  |  |
| 2L_12632220_SNP | 2.64E-07 | 0.083266 | FBgn0032439 |  |  |  |  |
| 2L_12632223_SNP | 2.64E-07 | 0.083266 | FBgn0032439 |  |  |  |  |
| 2L_2924056_SNP | 7.05E-07 | 0.159924 | FBgn0041111 |  |  |  |  |
| 3L_937224_SNP | 8.15E-07 | 0.159924 | FBgn0264574 |  |  |  |  |
| 3L_6357491_SNP | 8.63E-07 | 0.159924 |  |  |  |  |  |
| 2L_14593489_SNP | 8.86E-07 | 0.159924 |  |  |  |  |  |
| 2L_16367015_SNP | 9.3E-07 | 0.159924 | FBgn0028841 |  |  |  |  |
| 2R_4730821_SNP | 1.65E-06 | 0.200609 | FBgn0024189 |  |  |  |  |
| X_4360110_SNP | 1.8E-06 | 0.200609 | FBgn0000179 | FBgn0052773 |  |  |  |
| X_4360113_INS | 1.8E-06 | 0.200609 | FBgn0000179 | FBgn0052773 |  |  |  |
| X_4360120_INS | 1.95E-06 | 0.200609 | FBgn0000179 | FBgn0052773 |  |  |  |
| 2L_12758562_SNP | 2.26E-06 | 0.200609 |  |  |  |  |  |
| X_4360117_SNP | 2.31E-06 | 0.200609 | FBgn0052773 | FBgn0000179 |  |  |  |
| 2L_16046650_SNP | 2.33E-06 | 0.200609 | FBgn0000182 | FBgn0266459 |  |  |  |
| 3L_937216_SNP | 2.46E-06 | 0.200609 | FBgn0264574 |  |  |  |  |
| 2L_20049203_SNP | 2.6E-06 | 0.200609 | FBgn0032840 |  |  |  |  |
| 2L_2924011_SNP | 2.69E-06 | 0.200609 | FBgn0041111 |  |  |  |  |
| 2L_9014438_SNP | 2.73E-06 | 0.200609 | FBgn0032088 | FBgn0032089 |  |  |  |
| 2R_14617445_SNP | 2.93E-06 | 0.200609 | FBgn0053147 | FBgn0034381 |  |  |  |
| 3L_8030270_SNP | 2.96E-06 | 0.200609 | FBgn0011817 |  |  |  |  |
| 2R_12980044_SNP | 3.05E-06 | 0.200609 | FBgn0010591 | FBgn0010226 | FBgn0034191 |  |  |
| 2L_16309272_SNP | 3.08E-06 | 0.200609 | FBgn0000339 | FBgn0011708 |  |  |  |
| 2R_8899844_SNP | 3.15E-06 | 0.200609 |  |  |  |  |  |
| 2L_10131205_SNP | 3.21E-06 | 0.200609 | FBgn0265002 |  |  |  |  |
| X_11846956_SNP | 3.26E-06 | 0.200609 | FBgn0263111 |  |  |  |  |
| 2L_16045456_SNP | 3.28E-06 | 0.200609 | FBgn0000182 | FBgn0266459 |  |  |  |
| 2R_8899836_SNP | 3.35E-06 | 0.200609 |  |  |  |  |  |
| 2R_8899595_SNP | 3.46E-06 | 0.200609 |  |  |  |  |  |
| 2R_8899851_SNP | 3.59E-06 | 0.200609 |  |  |  |  |  |
| 2R_8899982_SNP | 3.61E-06 | 0.200609 |  |  |  |  |  |
| 2L_3449186_SNP | 3.85E-06 | 0.207844 | FBgn0031534 |  |  |  |  |
| 2R_4524827_SNP | 4.33E-06 | 0.226419 |  |  |  |  |  |
| X_13512987_SNP | 4.47E-06 | 0.226419 | FBgn0030503 | FBgn0030504 |  |  |  |
| 2R_8899678_SNP | 4.55E-06 | 0.226419 |  |  |  |  |  |
| 2L_16054897_SNP | 4.88E-06 | 0.236516 | FBgn0013433 |  |  |  |  |
| 2R_7065690_SNP | 5.41E-06 | 0.254727 | FBgn0003396 |  |  |  |  |
| 2R_8899584_SNP | 5.52E-06 | 0.254727 |  |  |  |  |  |
| 2L_16058280_SNP | 6.38E-06 | 0.276524 | FBgn0013433 |  |  |  |  |
| 3L_9939192_SNP | 6.4E-06 | 0.276524 | FBgn0085385 |  |  |  |  |
| 2R_5033826_SNP | 6.6E-06 | 0.276524 | FBgn0050344 |  |  |  |  |
| 3L_2992563_SNP | 6.64E-06 | 0.276524 |  |  |  |  |  |
| X_10946166_SNP | 6.81E-06 | 0.276524 | FBgn0030258 |  |  |  |  |
| 2L_16054772_SNP | 6.87E-06 | 0.276524 | FBgn0013433 |  |  |  |  |
| 3L_888960_INS | 7.51E-06 | 0.295897 | FBgn0052479 |  |  |  |  |
| 2R_20423839_SNP | 8.12E-06 | 0.310305 | FBgn0035021 | FBgn0035022 |  |  |  |
| 2L_16305028_SNP | 8.43E-06 | 0.310305 | FBgn0028525 | FBgn0015338 |  |  |  |
| 2R_16521194_SNP | 9.11E-06 | 0.310305 | FBgn0250850 | FBgn0034530 |  |  |  |
| 2R_13705136_SNP | 9.21E-06 | 0.310305 | FBgn0259211 |  |  |  |  |
| 3L_937304_DEL | 9.27E-06 | 0.310305 | FBgn0264574 |  |  |  |  |
| 2L_7098974_INS | 9.32E-06 | 0.310305 | FBgn0085407 |  |  |  |  |
| 3L_7331392_SNP | 9.34E-06 | 0.310305 | FBgn0035766 | FBgn0011640 |  |  |  |
| X_13513436_DEL | 9.55E-06 | 0.310305 | FBgn0030504 | FBgn0030503 |  |  |  |
| 2L_16042873_SNP | 9.6E-06 | 0.310305 | FBgn0000182 | FBgn0266459 |  |  |  |
| 2L_9766124_SNP | 9.73E-06 | 0.310305 | FBgn0032143 | FBgn0265001 | FBgn0025117 |  |  |
| 2L_16045275_SNP | 9.85E-06 | 0.310305 | FBgn0266459 | FBgn0000182 |  |  |  |
| 3L_8204998_SNP | 9.92E-06 | 0.310305 | FBgn0035857 |  |  |  |  |
| SNP | *P* | *P*_adjust_ | Flybase ID1 | Flybase ID2 | Flybase ID3 | Flybase ID4 | Flybase ID5 |
| **radius_mean_ sd** | | | | | | | |
| 2R_16322804_SNP | 3.97E-08 | 0.044513 | FBgn0086604 |  |  |  |  |
| X_4361594_SNP | 4.71E-08 | 0.044513 | FBgn0052773 | FBgn0000179 |  |  |  |
| X_4359575_SNP | 4.08E-07 | 0.1546 | FBgn0052773 | FBgn0000179 |  |  |  |
| 2L_16309272_SNP | 4.46E-07 | 0.1546 | FBgn0000339 | FBgn0011708 |  |  |  |
| 2L_16305028_SNP | 4.86E-07 | 0.1546 | FBgn0028525 | FBgn0015338 |  |  |  |
| 2L_16304318_SNP | 5.43E-07 | 0.1546 | FBgn0028525 |  |  |  |  |
| 2L_3449186_SNP | 5.72E-07 | 0.1546 | FBgn0031534 |  |  |  |  |
| X_4359702_SNP | 6.72E-07 | 0.158841 | FBgn0000179 | FBgn0052773 |  |  |  |
| 2L_16367015_SNP | 8.36E-07 | 0.161441 | FBgn0028841 |  |  |  |  |
| 2L_2898419_SNP | 8.89E-07 | 0.161441 | FBgn0041111 |  |  |  |  |
| 2R_12980044_SNP | 9.76E-07 | 0.161441 | FBgn0010591 | FBgn0010226 | FBgn0034191 |  |  |
| 2L_16306935_SNP | 1.13E-06 | 0.161441 | FBgn0028525 | FBgn0015338 | FBgn0011708 |  |  |
| 2L_3577037_SNP | 1.17E-06 | 0.161441 | FBgn0004892 | FBgn0264363 |  |  |  |
| 2L_16046650_SNP | 1.24E-06 | 0.161441 | FBgn0000182 | FBgn0266459 |  |  |  |
| 2L_16304529_SNP | 1.31E-06 | 0.161441 | FBgn0028525 |  |  |  |  |
| 2L_16304312_INS | 1.37E-06 | 0.161441 | FBgn0028525 |  |  |  |  |
| X_10343822_SNP | 1.53E-06 | 0.167351 | FBgn0052683 |  |  |  |  |
| 2L_18818411_SNP | 1.59E-06 | 0.167351 | FBgn0027074 |  |  |  |  |
| 3L_937224_SNP | 1.91E-06 | 0.182884 | FBgn0264574 |  |  |  |  |
| 2L_16054927_SNP | 1.98E-06 | 0.182884 | FBgn0013433 |  |  |  |  |
| 2L_16300118_SNP | 2.26E-06 | 0.182884 | FBgn0028375 | FBgn0028895 |  |  |  |
| 2R_18528113_SNP | 2.29E-06 | 0.182884 | FBgn0261596 | FBgn0034750 |  |  |  |
| 2L_16303958_SNP | 2.41E-06 | 0.182884 | FBgn0028525 |  |  |  |  |
| 2R_16521194_SNP | 2.49E-06 | 0.182884 | FBgn0250850 | FBgn0034530 |  |  |  |
| 2L_17247223_SNP | 2.65E-06 | 0.182884 | FBgn0265546 | FBgn0032629 | FBgn0265545 |  |  |
| 3L_8030270_SNP | 2.71E-06 | 0.182884 | FBgn0011817 |  |  |  |  |
| 3L_11328592_SNP | 2.81E-06 | 0.182884 |  |  |  |  |  |
| X_8687452_SNP | 2.81E-06 | 0.182884 | FBgn0026411 |  |  |  |  |
| 2L_7098974_INS | 3.05E-06 | 0.182884 | FBgn0085407 |  |  |  |  |
| 3L_7331392_SNP | 3.08E-06 | 0.182884 | FBgn0035766 | FBgn0011640 |  |  |  |
| 2R_16204841_SNP | 3.29E-06 | 0.182884 | FBgn0034501 | FBgn0034502 |  |  |  |
| 2R_18276242_SNP | 3.3E-06 | 0.182884 | FBgn0265978 | FBgn0034723 |  |  |  |
| 2R_20423839_SNP | 3.53E-06 | 0.182884 | FBgn0035021 | FBgn0035022 |  |  |  |
| 3L_6357491_SNP | 3.53E-06 | 0.182884 |  |  |  |  |  |
| 2L_16054897_SNP | 3.54E-06 | 0.182884 | FBgn0013433 |  |  |  |  |
| 2L_20049203_SNP | 3.63E-06 | 0.182884 | FBgn0032840 |  |  |  |  |
| X_9675812_SNP | 3.68E-06 | 0.182884 | FBgn0030151 |  |  |  |  |
| 2L_3449578_SNP | 3.94E-06 | 0.182884 | FBgn0031534 |  |  |  |  |
| 2L_16042873_SNP | 3.97E-06 | 0.182884 | FBgn0000182 | FBgn0266459 |  |  |  |
| 2L_9014438_SNP | 3.98E-06 | 0.182884 | FBgn0032088 | FBgn0032089 |  |  |  |
| 2L_17247224_DEL | 4E-06 | 0.182884 | FBgn0032629 | FBgn0265545 | FBgn0265546 |  |  |
| 2L_16045456_SNP | 4.06E-06 | 0.182884 | FBgn0000182 | FBgn0266459 |  |  |  |
| 2L_2487012_SNP | 4.44E-06 | 0.194005 |  |  |  |  |  |
| 2R_3888045_SNP | 4.66E-06 | 0.194005 | FBgn0033249 |  |  |  |  |
| 2R_1716201_SNP | 4.66E-06 | 0.194005 | FBgn0085414 |  |  |  |  |
| 2R_1720275_SNP | 4.75E-06 | 0.194005 | FBgn0085414 |  |  |  |  |
| 2L_3449307_SNP | 4.82E-06 | 0.194005 | FBgn0031534 |  |  |  |  |
| 2L_16054772_SNP | 5.13E-06 | 0.195376 | FBgn0013433 |  |  |  |  |
| 2L_16058280_SNP | 5.23E-06 | 0.195376 | FBgn0013433 |  |  |  |  |
| 3L_14200065_SNP | 5.29E-06 | 0.195376 | FBgn0013718 |  |  |  |  |
| 2L_12758562_SNP | 5.32E-06 | 0.195376 |  |  |  |  |  |
| 2R_1731235_SNP | 5.4E-06 | 0.195376 | FBgn0085414 |  |  |  |  |
| 2L_16054914_SNP | 5.48E-06 | 0.195376 | FBgn0013433 |  |  |  |  |
| 3R_3383635_DEL | 5.59E-06 | 0.195575 |  |  |  |  |  |
| 2L_3449679_SNP | 5.75E-06 | 0.197692 | FBgn0031534 |  |  |  |  |
| 2L_10131205_SNP | 5.93E-06 | 0.199149 | FBgn0265002 |  |  |  |  |
| 3L_8578310_SNP | 6E-06 | 0.199149 | FBgn0262508 |  |  |  |  |
| 2L_13639699_SNP | 6.37E-06 | 0.200653 | FBgn0259984 | FBgn0028546 |  |  |  |
| 2R_9900089_SNP | 6.46E-06 | 0.200653 | FBgn0002643 |  |  |  |  |
| X_10737059_INS | 6.55E-06 | 0.200653 | FBgn0030237 | FBgn0052669 | FBgn0003321 |  |  |
| X_11846956_SNP | 6.57E-06 | 0.200653 | FBgn0263111 |  |  |  |  |
| 2L_12632220_SNP | 6.69E-06 | 0.200653 | FBgn0032439 |  |  |  |  |
| 2L_12632223_SNP | 6.69E-06 | 0.200653 | FBgn0032439 |  |  |  |  |
| 2R_1741175_SNP | 7.26E-06 | 0.207837 | FBgn0085414 |  |  |  |  |
| 2L_16055067_SNP | 7.31E-06 | 0.207837 | FBgn0013433 |  |  |  |  |
| 2L_16045275_SNP | 7.45E-06 | 0.207837 | FBgn0266459 | FBgn0000182 |  |  |  |
| 2L_16054766_SNP | 7.55E-06 | 0.207837 | FBgn0013433 |  |  |  |  |
| 2L_16054971_SNP | 8.05E-06 | 0.207837 | FBgn0013433 |  |  |  |  |
| 3L_9300629_SNP | 8.13E-06 | 0.207837 |  |  |  |  |  |
| 3R_13596133_SNP | 8.13E-06 | 0.207837 | FBgn0262562 |  |  |  |  |
| 2R_7065690_SNP | 8.23E-06 | 0.207837 | FBgn0003396 |  |  |  |  |
| 2L_794993_SNP | 8.38E-06 | 0.207837 |  |  |  |  |  |
| 2R_4730821_SNP | 8.4E-06 | 0.207837 | FBgn0024189 |  |  |  |  |
| 3R_13596118_SNP | 8.44E-06 | 0.207837 | FBgn0262562 |  |  |  |  |
| 2L_16054939_DEL | 8.45E-06 | 0.207837 | FBgn0013433 |  |  |  |  |
| 2L_15617707_SNP | 8.49E-06 | 0.207837 | FBgn0028947 |  |  |  |  |
| X_13454996_SNP | 8.55E-06 | 0.207837 | FBgn0052633 |  |  |  |  |
| 2R_1712204_SNP | 8.57E-06 | 0.207837 | FBgn0085414 |  |  |  |  |
| 2L_16055041_SNP | 8.99E-06 | 0.211316 | FBgn0013433 |  |  |  |  |
| 2L_16205665_SNP | 9.13E-06 | 0.211316 |  |  |  |  |  |
| 2L_14976839_SNP | 9.22E-06 | 0.211316 | FBgn0086711 |  |  |  |  |
| 2L_14593489_SNP | 9.48E-06 | 0.211316 |  |  |  |  |  |
| 2L_16054950_SNP | 9.48E-06 | 0.211316 | FBgn0013433 |  |  |  |  |
| 2L_16301194_SNP | 9.54E-06 | 0.211316 | FBgn0028895 | FBgn0028894 |  |  |  |
| 2L_16301275_SNP | 9.57E-06 | 0.211316 | FBgn0028895 | FBgn0028894 |  |  |  |
| 2L_16054988_SNP | 9.72E-06 | 0.211316 | FBgn0013433 |  |  |  |  |
| X_3317641_INS | 9.83E-06 | 0.211316 | FBgn0029657 | FBgn0266350 |  |  |  |
| X_3317633_SNP | 9.83E-06 | 0.211316 | FBgn0266350 | FBgn0029657 |  |  |  |
| 3L_937216_SNP | 9.96E-06 | 0.211618 | FBgn0264574 |  |  |  |  |
| SNP | *P* | *P*_adjust_ | Flybase ID1 | Flybase ID2 | Flybase ID3 | Flybase ID4 | Flybase ID5 |
| **perimeter sd** | | | | | | | |
| 2R_16322804_SNP | 5.96E-09 | 0.011276 | FBgn0086604 |  |  |  |  |
| X_4359575_SNP | 1.98E-08 | 0.018739 | FBgn0052773 | FBgn0000179 |  |  |  |
| X_4361594_SNP | 3.39E-08 | 0.021393 | FBgn0052773 | FBgn0000179 |  |  |  |
| X_4359702_SNP | 6.39E-08 | 0.030194 | FBgn0000179 | FBgn0052773 |  |  |  |
| X_4360110_SNP | 1.51E-07 | 0.039559 | FBgn0000179 | FBgn0052773 |  |  |  |
| X_4360113_INS | 1.51E-07 | 0.039559 | FBgn0000179 | FBgn0052773 |  |  |  |
| 2L_16046650_SNP | 1.68E-07 | 0.039559 | FBgn0000182 | FBgn0266459 |  |  |  |
| X_4360120_INS | 1.69E-07 | 0.039559 | FBgn0000179 | FBgn0052773 |  |  |  |
| 3L_7331392_SNP | 1.89E-07 | 0.039559 | FBgn0035766 | FBgn0011640 |  |  |  |
| X_4360117_SNP | 2.09E-07 | 0.039559 | FBgn0052773 | FBgn0000179 |  |  |  |
| 3L_8030270_SNP | 2.69E-07 | 0.046294 | FBgn0011817 |  |  |  |  |
| 2L_16058280_SNP | 3.23E-07 | 0.04854 | FBgn0013433 |  |  |  |  |
| 2R_16204841_SNP | 3.34E-07 | 0.04854 | FBgn0034501 | FBgn0034502 |  |  |  |
| 2L_10131205_SNP | 5E-07 | 0.057433 | FBgn0265002 |  |  |  |  |
| 2L_12632220_SNP | 5.32E-07 | 0.057433 | FBgn0032439 |  |  |  |  |
| 2L_12632223_SNP | 5.32E-07 | 0.057433 | FBgn0032439 |  |  |  |  |
| 2L_16042873_SNP | 5.46E-07 | 0.057433 | FBgn0000182 | FBgn0266459 |  |  |  |
| 2L_16045456_SNP | 5.47E-07 | 0.057433 | FBgn0000182 | FBgn0266459 |  |  |  |
| X_4357745_SNP | 7.43E-07 | 0.073907 | FBgn0000179 | FBgn0052773 |  |  |  |
| X_9675812_SNP | 9.97E-07 | 0.094265 | FBgn0030151 |  |  |  |  |
| 2L_16054897_SNP | 1.05E-06 | 0.094458 | FBgn0013433 |  |  |  |  |
| 2R_20423839_SNP | 1.23E-06 | 0.097801 | FBgn0035021 | FBgn0035022 |  |  |  |
| 2L_9014438_SNP | 1.24E-06 | 0.097801 | FBgn0032088 | FBgn0032089 |  |  |  |
| 2L_8915684_SNP | 1.28E-06 | 0.097801 | FBgn0085427 |  |  |  |  |
| 2L_2898419_SNP | 1.29E-06 | 0.097801 | FBgn0041111 |  |  |  |  |
| 2L_14593489_SNP | 1.64E-06 | 0.108334 |  |  |  |  |  |
| 2R_3888045_SNP | 1.67E-06 | 0.108334 | FBgn0033249 |  |  |  |  |
| X_11846956_SNP | 1.73E-06 | 0.108334 | FBgn0263111 |  |  |  |  |
| 2L_16043275_SNP | 1.76E-06 | 0.108334 | FBgn0000182 | FBgn0266459 |  |  |  |
| 2L_16041793_SNP | 1.76E-06 | 0.108334 | FBgn0028644 | FBgn0000182 | FBgn0266459 |  |  |
| 2R_4730821_SNP | 1.78E-06 | 0.108334 | FBgn0024189 |  |  |  |  |
| 2L_16309272_SNP | 1.99E-06 | 0.116268 | FBgn0000339 | FBgn0011708 |  |  |  |
| X_4357669_SNP | 2.13E-06 | 0.116268 | FBgn0000179 | FBgn0052773 |  |  |  |
| 3L_937224_SNP | 2.14E-06 | 0.116268 | FBgn0264574 |  |  |  |  |
| 2L_16205665_SNP | 2.15E-06 | 0.116268 |  |  |  |  |  |
| 2L_2924056_SNP | 2.27E-06 | 0.119182 | FBgn0041111 |  |  |  |  |
| X_10946166_SNP | 2.33E-06 | 0.119182 | FBgn0030258 |  |  |  |  |
| 2R_9900089_SNP | 2.47E-06 | 0.122913 | FBgn0002643 |  |  |  |  |
| 2L_16054772_SNP | 2.73E-06 | 0.132513 | FBgn0013433 |  |  |  |  |
| 3L_9939192_SNP | 3.02E-06 | 0.137132 | FBgn0085385 |  |  |  |  |
| 2L_18818411_SNP | 3.12E-06 | 0.137132 | FBgn0027074 |  |  |  |  |
| 3L_14200065_SNP | 3.24E-06 | 0.137132 | FBgn0013718 |  |  |  |  |
| X_4357519_SNP | 3.38E-06 | 0.137132 | FBgn0052773 | FBgn0000179 |  |  |  |
| 2L_17529444_SNP | 3.4E-06 | 0.137132 | FBgn0032648 | FBgn0032649 |  |  |  |
| 2L_7098974_INS | 3.51E-06 | 0.137132 | FBgn0085407 |  |  |  |  |
| 3L_15811224_SNP | 3.61E-06 | 0.137132 | FBgn0036534 | FBgn0015025 |  |  |  |
| X_9674859_SNP | 3.62E-06 | 0.137132 | FBgn0030151 |  |  |  |  |
| X_9675398_SNP | 3.62E-06 | 0.137132 | FBgn0030151 |  |  |  |  |
| X_9678441_SNP | 3.62E-06 | 0.137132 | FBgn0052698 |  |  |  |  |
| 2L_20049203_SNP | 3.7E-06 | 0.137132 | FBgn0032840 |  |  |  |  |
| 2L_16055067_SNP | 3.72E-06 | 0.137132 | FBgn0013433 |  |  |  |  |
| 2L_2924011_SNP | 3.77E-06 | 0.137132 | FBgn0041111 |  |  |  |  |
| X_9678155_SNP | 4.12E-06 | 0.147067 | FBgn0052698 |  |  |  |  |
| 3L_8578310_SNP | 4.3E-06 | 0.150647 | FBgn0262508 |  |  |  |  |
| 2L_16054766_SNP | 4.56E-06 | 0.156813 | FBgn0013433 |  |  |  |  |
| 2L_11296469_SNP | 4.76E-06 | 0.158432 |  |  |  |  |  |
| 2L_16054914_SNP | 4.85E-06 | 0.158432 | FBgn0013433 |  |  |  |  |
| 3L_4798559_SNP | 4.87E-06 | 0.158432 |  |  |  |  |  |
| 3L_7331522_SNP | 5E-06 | 0.158432 | FBgn0035766 | FBgn0011640 |  |  |  |
| 2L_16367015_SNP | 5.03E-06 | 0.158432 | FBgn0028841 |  |  |  |  |
| 2L_16054939_DEL | 5.18E-06 | 0.160515 | FBgn0013433 |  |  |  |  |
| 2L_16054971_SNP | 5.29E-06 | 0.161281 | FBgn0013433 |  |  |  |  |
| X_10343822_SNP | 5.38E-06 | 0.161483 | FBgn0052683 |  |  |  |  |
| 2L_16054927_SNP | 5.63E-06 | 0.165293 | FBgn0013433 |  |  |  |  |
| 2R_14617445_SNP | 5.82E-06 | 0.165293 | FBgn0053147 | FBgn0034381 |  |  |  |
| 2L_9766124_SNP | 5.86E-06 | 0.165293 | FBgn0032143 | FBgn0265001 | FBgn0025117 |  |  |
| 2L_16043209_SNP | 5.94E-06 | 0.165293 | FBgn0266459 | FBgn0000182 |  |  |  |
| 2L_16054988_SNP | 5.94E-06 | 0.165293 | FBgn0013433 |  |  |  |  |
| 3L_7807639_SNP | 6.1E-06 | 0.167118 | FBgn0016694 |  |  |  |  |
| 2L_16054950_SNP | 6.2E-06 | 0.167405 | FBgn0013433 |  |  |  |  |
| 2L_16054936_SNP | 6.91E-06 | 0.18014 | FBgn0013433 |  |  |  |  |
| 2L_16042821_SNP | 6.93E-06 | 0.18014 | FBgn0000182 | FBgn0266459 |  |  |  |
| 2R_14572645_SNP | 6.99E-06 | 0.18014 | FBgn0265625 | FBgn0265626 | FBgn0265627 |  |  |
| 2L_16058297_SNP | 7.06E-06 | 0.18014 | FBgn0013433 |  |  |  |  |
| 2L_5092670_SNP | 7.17E-06 | 0.18014 | FBgn0031681 |  |  |  |  |
| 2R_13324519_SNP | 7.24E-06 | 0.18014 | FBgn0027506 |  |  |  |  |
| 3L_14481388_SNP | 7.43E-06 | 0.181102 | FBgn0087007 |  |  |  |  |
| X_4357496_SNP | 7.5E-06 | 0.181102 | FBgn0052773 | FBgn0000179 |  |  |  |
| 3L_2692607_SNP | 7.57E-06 | 0.181102 | FBgn0264606 |  |  |  |  |
| 2L_16058307_SNP | 8.12E-06 | 0.186659 | FBgn0013433 |  |  |  |  |
| 3L_937216_SNP | 8.14E-06 | 0.186659 | FBgn0264574 |  |  |  |  |
| 2L_4294028_SNP | 8.16E-06 | 0.186659 | FBgn0010473 |  |  |  |  |
| 3R_17227772_SNP | 8.19E-06 | 0.186659 | FBgn0263003 |  |  |  |  |
| X_9678636_SNP | 8.37E-06 | 0.186917 | FBgn0052698 |  |  |  |  |
| 2L_2487012_SNP | 8.4E-06 | 0.186917 |  |  |  |  |  |
| X_19768593_SNP | 8.53E-06 | 0.187448 | FBgn0052529 |  |  |  |  |
| X_17261510_SNP | 8.8E-06 | 0.191249 |  |  |  |  |  |
| X_9673653_SNP | 8.98E-06 | 0.191923 | FBgn0030151 |  |  |  |  |
| 2L_16045275_SNP | 9.03E-06 | 0.191923 | FBgn0266459 | FBgn0000182 |  |  |  |
| X_4357559_SNP | 9.16E-06 | 0.192235 | FBgn0052773 | FBgn0000179 |  |  |  |
| 2R_7065690_SNP | 9.25E-06 | 0.192235 | FBgn0003396 |  |  |  |  |
| X_9678799_SNP | 9.52E-06 | 0.195654 | FBgn0052698 |  |  |  |  |
| SNP | *P* | *P*_adjust_ | Flybase ID1 | Flybase ID2 | Flybase ID3 | Flybase ID4 | Flybase ID5 |
| **radius_min_ sd** | | | | | | | |
| 2L_12384753_SNP | 3.12E-07 | 0.525419 | FBgn0262475 |  |  |  |  |
| 2L_7722134_DEL | 1.1E-06 | 0.525419 | FBgn0031936 | FBgn0031937 |  |  |  |
| 2L_1370014_SNP | 1.24E-06 | 0.525419 | FBgn0031337 |  |  |  |  |
| 2L_12384797_SNP | 1.35E-06 | 0.525419 | FBgn0262475 |  |  |  |  |
| 3R_5091992_SNP | 1.69E-06 | 0.525419 | FBgn0010803 | FBgn0261241 |  |  |  |
| 2L_3195830_SNP | 1.9E-06 | 0.525419 | FBgn0085422 |  |  |  |  |
| 3R_1297418_DEL | 1.95E-06 | 0.525419 | FBgn0011715 |  |  |  |  |
| 3L_3114013_SNP | 2.69E-06 | 0.634656 | FBgn0026189 | FBgn0266437 |  |  |  |
| 2L_11642828_SNP | 3.28E-06 | 0.688522 |  |  |  |  |  |
| 3R_5352797_SNP | 4.69E-06 | 0.813976 | FBgn0037702 |  |  |  |  |
| 2L_6813795_SNP | 4.74E-06 | 0.813976 | FBgn0264890 |  |  |  |  |
| 2L_11642741_SNP | 5.33E-06 | 0.832404 |  |  |  |  |  |
| 2L_10685025_SNP | 6.12E-06 | 0.832404 | FBgn0032282 |  |  |  |  |
| 2L_8402304_SNP | 6.38E-06 | 0.832404 | FBgn0051898 | FBgn0001084 |  |  |  |
| 2R_12601327_SNP | 6.6E-06 | 0.832404 | FBgn0050463 |  |  |  |  |
| 3R_10532800_SNP | 7.23E-06 | 0.842593 | FBgn0262127 |  |  |  |  |
| 2R_5221788_SNP | 7.58E-06 | 0.842593 | FBgn0033405 | FBgn0033403 |  |  |  |
| 2L_11642762_SNP | 8.54E-06 | 0.896949 |  |  |  |  |  |
| X_4892904_SNP | 9.56E-06 | 0.951455 | FBgn0266096 | FBgn0029740 |  |  |  |
| SNP | *P* | *P*_adjust_ | Flybase ID1 | Flybase ID2 | Flybase ID3 | Flybase ID4 | Flybase ID5 |
| **radius_max_ sd** | | | | | | | |
| 2R_16322804_SNP | 7.33E-08 | 0.138627 | FBgn0086604 |  |  |  |  |
| X_4361594_SNP | 4.1E-07 | 0.2798 | FBgn0052773 | FBgn0000179 |  |  |  |
| X_4359575_SNP | 4.44E-07 | 0.2798 | FBgn0052773 | FBgn0000179 |  |  |  |
| 2L_18818411_SNP | 6.56E-07 | 0.297888 | FBgn0027074 |  |  |  |  |
| X_4359702_SNP | 9.65E-07 | 0.297888 | FBgn0000179 | FBgn0052773 |  |  |  |
| 2L_16367015_SNP | 1.83E-06 | 0.297888 | FBgn0028841 |  |  |  |  |
| 2R_1716201_SNP | 1.87E-06 | 0.297888 | FBgn0085414 |  |  |  |  |
| 2L_17247223_SNP | 1.94E-06 | 0.297888 | FBgn0265546 | FBgn0032629 | FBgn0265545 |  |  |
| 3L_6357491_SNP | 2.01E-06 | 0.297888 |  |  |  |  |  |
| 2L_7098974_INS | 2.64E-06 | 0.297888 | FBgn0085407 |  |  |  |  |
| 2L_17247224_DEL | 2.66E-06 | 0.297888 | FBgn0032629 | FBgn0265545 | FBgn0265546 |  |  |
| 3R_13596118_SNP | 2.69E-06 | 0.297888 | FBgn0262562 |  |  |  |  |
| 3R_13596133_SNP | 2.79E-06 | 0.297888 | FBgn0262562 |  |  |  |  |
| 2R_16521194_SNP | 2.88E-06 | 0.297888 | FBgn0250850 | FBgn0034530 |  |  |  |
| 2L_16309272_SNP | 3E-06 | 0.297888 | FBgn0000339 | FBgn0011708 |  |  |  |
| 2L_16305028_SNP | 3.1E-06 | 0.297888 | FBgn0028525 | FBgn0015338 |  |  |  |
| 2R_4524827_SNP | 3.26E-06 | 0.297888 |  |  |  |  |  |
| 2R_8899982_SNP | 3.84E-06 | 0.297888 |  |  |  |  |  |
| 3R_13596163_SNP | 3.88E-06 | 0.297888 | FBgn0262562 |  |  |  |  |
| 2R_12980044_SNP | 4.04E-06 | 0.297888 | FBgn0010591 | FBgn0010226 | FBgn0034191 |  |  |
| 2L_16304318_SNP | 4.13E-06 | 0.297888 | FBgn0028525 |  |  |  |  |
| 2L_4294028_SNP | 4.18E-06 | 0.297888 | FBgn0010473 |  |  |  |  |
| 2L_3449186_SNP | 4.29E-06 | 0.297888 | FBgn0031534 |  |  |  |  |
| 2L_4646519_SNP | 4.58E-06 | 0.297888 | FBgn0264342 | FBgn0031617 |  |  |  |
| 3L_8030270_SNP | 4.9E-06 | 0.297888 | FBgn0011817 |  |  |  |  |
| 2R_1731235_SNP | 4.92E-06 | 0.297888 | FBgn0085414 |  |  |  |  |
| 2R_1741175_SNP | 5.56E-06 | 0.297888 | FBgn0085414 |  |  |  |  |
| 2L_2487012_SNP | 5.63E-06 | 0.297888 |  |  |  |  |  |
| 2R_8899595_SNP | 5.71E-06 | 0.297888 |  |  |  |  |  |
| 2R_1720275_SNP | 5.72E-06 | 0.297888 | FBgn0085414 |  |  |  |  |
| 3R_13961960_SNP | 5.73E-06 | 0.297888 | FBgn0003499 |  |  |  |  |
| 2R_8899836_SNP | 5.75E-06 | 0.297888 |  |  |  |  |  |
| 3L_937224_SNP | 5.83E-06 | 0.297888 | FBgn0264574 |  |  |  |  |
| 2R_8899844_SNP | 5.99E-06 | 0.297888 |  |  |  |  |  |
| 3R_13595983_SNP | 6.09E-06 | 0.297888 | FBgn0262562 |  |  |  |  |
| 2R_19531407_SNP | 6.24E-06 | 0.297888 | FBgn0004795 |  |  |  |  |
| 2L_12758562_SNP | 6.26E-06 | 0.297888 |  |  |  |  |  |
| 2R_8899678_SNP | 6.47E-06 | 0.297888 |  |  |  |  |  |
| 2L_9235056_SNP | 6.59E-06 | 0.297888 | FBgn0041092 |  |  |  |  |
| 2R_8899851_SNP | 6.62E-06 | 0.297888 |  |  |  |  |  |
| 2R_1712204_SNP | 6.72E-06 | 0.297888 | FBgn0085414 |  |  |  |  |
| 2R_8899584_SNP | 6.98E-06 | 0.297888 |  |  |  |  |  |
| 3L_8578310_SNP | 7.14E-06 | 0.297888 | FBgn0262508 |  |  |  |  |
| 2L_9014438_SNP | 7.27E-06 | 0.297888 | FBgn0032088 | FBgn0032089 |  |  |  |
| 2R_18528113_SNP | 7.35E-06 | 0.297888 | FBgn0261596 | FBgn0034750 |  |  |  |
| 2L_11732243_SNP | 7.4E-06 | 0.297888 |  |  |  |  |  |
| 2L_17707793_SNP | 7.4E-06 | 0.297888 | FBgn0015609 |  |  |  |  |
| 2L_16046650_SNP | 7.8E-06 | 0.306182 | FBgn0000182 | FBgn0266459 |  |  |  |
| 2R_16204841_SNP | 7.93E-06 | 0.306182 | FBgn0034501 | FBgn0034502 |  |  |  |
| 2L_20049203_SNP | 8.52E-06 | 0.31228 | FBgn0032840 |  |  |  |  |
| 3L_19791791_SNP | 8.57E-06 | 0.31228 | FBgn0261283 | FBgn0262393 | FBgn0260874 | FBgn0036906 |  |
| 2L_16304312_INS | 8.93E-06 | 0.31228 | FBgn0028525 |  |  |  |  |
| 2R_3226958_SNP | 9.07E-06 | 0.31228 | FBgn0033159 |  |  |  |  |
| 2L_3577037_SNP | 9.56E-06 | 0.31228 | FBgn0004892 | FBgn0264363 |  |  |  |
| 2R_11385557_SNP | 9.6E-06 | 0.31228 | FBgn0034022 |  |  |  |  |
| 2L_8715468_SNP | 9.69E-06 | 0.31228 | FBgn0003209 |  |  |  |  |
| X_8687452_SNP | 9.93E-06 | 0.31228 | FBgn0026411 |  |  |  |  |
| SNP | *P* | *P*_adjust_ | Flybase ID1 | Flybase ID2 | Flybase ID3 | Flybase ID4 | Flybase ID5 |
| **radius_sd_ sd** | | | | | | | |
| X_4361594_SNP | 1.1E-07 | 0.204295 | FBgn0052773 | FBgn0000179 |  |  |  |
| 2R_16322804_SNP | 3.39E-07 | 0.204295 | FBgn0086604 |  |  |  |  |
| 3L_13835541_SNP | 6.39E-07 | 0.204295 | FBgn0036380 | FBgn0040812 |  |  |  |
| X_4359575_SNP | 7.36E-07 | 0.204295 | FBgn0052773 | FBgn0000179 |  |  |  |
| 2R_16521194_SNP | 7.73E-07 | 0.204295 | FBgn0250850 | FBgn0034530 |  |  |  |
| 3L_12598275_INS | 1.12E-06 | 0.204295 |  |  |  |  |  |
| X_4359702_SNP | 1.37E-06 | 0.204295 | FBgn0000179 | FBgn0052773 |  |  |  |
| 3L_11328592_SNP | 1.46E-06 | 0.204295 |  |  |  |  |  |
| 3L_15386008_SNP | 1.71E-06 | 0.204295 |  |  |  |  |  |
| 2L_3609725_INS | 1.75E-06 | 0.204295 |  |  |  |  |  |
| 2L_16309272_SNP | 1.79E-06 | 0.204295 | FBgn0000339 | FBgn0011708 |  |  |  |
| 3L_7331392_SNP | 1.92E-06 | 0.204295 | FBgn0035766 | FBgn0011640 |  |  |  |
| 2L_16305028_SNP | 2.21E-06 | 0.204295 | FBgn0028525 | FBgn0015338 |  |  |  |
| 3L_14200065_SNP | 2.34E-06 | 0.204295 | FBgn0013718 |  |  |  |  |
| 2L_7098974_INS | 2.4E-06 | 0.204295 | FBgn0085407 |  |  |  |  |
| 2L_15753367_SNP | 2.47E-06 | 0.204295 | FBgn0001986 |  |  |  |  |
| X_9675812_SNP | 2.49E-06 | 0.204295 | FBgn0030151 |  |  |  |  |
| 3L_2692607_SNP | 2.63E-06 | 0.204295 | FBgn0264606 |  |  |  |  |
| 2L_22610747_SNP | 2.64E-06 | 0.204295 | FBgn0058006 |  |  |  |  |
| 2R_12453022_SNP | 2.68E-06 | 0.204295 | FBgn0034137 | FBgn0034136 |  |  |  |
| 2L_16304312_INS | 2.74E-06 | 0.204295 | FBgn0028525 |  |  |  |  |
| X_9674859_SNP | 2.74E-06 | 0.204295 | FBgn0030151 |  |  |  |  |
| X_9675398_SNP | 2.74E-06 | 0.204295 | FBgn0030151 |  |  |  |  |
| X_9678441_SNP | 2.74E-06 | 0.204295 | FBgn0052698 |  |  |  |  |
| 2L_16367015_SNP | 2.77E-06 | 0.204295 | FBgn0028841 |  |  |  |  |
| 2L_16304318_SNP | 2.85E-06 | 0.204295 | FBgn0028525 |  |  |  |  |
| 2L_10794310_SNP | 2.92E-06 | 0.204295 | FBgn0051869 |  |  |  |  |
| X_8687452_SNP | 3.21E-06 | 0.213027 | FBgn0026411 |  |  |  |  |
| 2L_4646519_SNP | 3.27E-06 | 0.213027 | FBgn0264342 | FBgn0031617 |  |  |  |
| 2L_3449186_SNP | 3.81E-06 | 0.224179 | FBgn0031534 |  |  |  |  |
| 2L_16304529_SNP | 3.89E-06 | 0.224179 | FBgn0028525 |  |  |  |  |
| 2R_9900089_SNP | 4.18E-06 | 0.224179 | FBgn0002643 |  |  |  |  |
| 2L_837497_SNP | 4.2E-06 | 0.224179 | FBgn0020304 |  |  |  |  |
| 2L_16306935_SNP | 4.23E-06 | 0.224179 | FBgn0028525 | FBgn0015338 | FBgn0011708 |  |  |
| 2L_2487012_SNP | 4.41E-06 | 0.224179 |  |  |  |  |  |
| 2R_14284032_SNP | 4.46E-06 | 0.224179 | FBgn0063499 |  |  |  |  |
| 3L_19791791_SNP | 4.47E-06 | 0.224179 | FBgn0261283 | FBgn0262393 | FBgn0260874 | FBgn0036906 |  |
| 2L_9235056_SNP | 4.51E-06 | 0.224179 | FBgn0041092 |  |  |  |  |
| 2L_9014438_SNP | 4.94E-06 | 0.230698 | FBgn0032088 | FBgn0032089 |  |  |  |
| 2R_18528113_SNP | 5.09E-06 | 0.230698 | FBgn0261596 | FBgn0034750 |  |  |  |
| 2L_16300118_SNP | 5.11E-06 | 0.230698 | FBgn0028375 | FBgn0028895 |  |  |  |
| 3L_9939192_SNP | 5.88E-06 | 0.230698 | FBgn0085385 |  |  |  |  |
| 3L_937224_SNP | 5.97E-06 | 0.230698 | FBgn0264574 |  |  |  |  |
| 3L_8204998_SNP | 6.01E-06 | 0.230698 | FBgn0035857 |  |  |  |  |
| 2R_8899982_SNP | 6.05E-06 | 0.230698 |  |  |  |  |  |
| 2R_17677991_SNP | 6.06E-06 | 0.230698 | FBgn0034660 |  |  |  |  |
| 2R_15163714_SNP | 6.07E-06 | 0.230698 | FBgn0003254 |  |  |  |  |
| 3L_4018632_DEL | 6.29E-06 | 0.230698 | FBgn0004895 |  |  |  |  |
| 2L_17529444_SNP | 6.4E-06 | 0.230698 | FBgn0032648 | FBgn0032649 |  |  |  |
| 2R_17395557_SNP | 6.46E-06 | 0.230698 | FBgn0043070 |  |  |  |  |
| X_14278085_SNP | 6.53E-06 | 0.230698 | FBgn0052600 |  |  |  |  |
| 2L_16303958_SNP | 6.54E-06 | 0.230698 | FBgn0028525 |  |  |  |  |
| 2L_9234952_DEL | 6.59E-06 | 0.230698 | FBgn0041092 |  |  |  |  |
| 2L_9234970_DEL | 6.59E-06 | 0.230698 | FBgn0041092 |  |  |  |  |
| 3L_8204999_SNP | 6.82E-06 | 0.234343 | FBgn0035857 |  |  |  |  |
| X_9678155_SNP | 7.58E-06 | 0.255821 | FBgn0052698 |  |  |  |  |
| 2L_10794313_SNP | 7.76E-06 | 0.257371 | FBgn0051869 |  |  |  |  |
| 3L_7941823_SNP | 7.95E-06 | 0.25903 | FBgn0041156 |  |  |  |  |
| 2R_4524827_SNP | 8.1E-06 | 0.259543 |  |  |  |  |  |
| 2R_17392016_SNP | 8.53E-06 | 0.26675 | FBgn0043070 |  |  |  |  |
| 2R_1385774_SNP | 8.61E-06 | 0.26675 | FBgn0050438 |  |  |  |  |
| 2L_12758562_SNP | 9.02E-06 | 0.275136 |  |  |  |  |  |
| 2R_1853082_SNP | 9.49E-06 | 0.284786 | FBgn0033048 |  |  |  |  |
| X_9678374_SNP | 9.85E-06 | 0.287931 | FBgn0052698 |  |  |  |  |
