# Supplementary material for: Using Drosophila to identify naturally occurring genetic modifiers of amyloid beta 42- and tau-induced toxicity
Source: G3 (Bethesda). 2023 Jun 13;13(9):jkad132. doi: 10.1093/g3journal/jkad132 (PMC10468303; doi:10.1093/g3journal/jkad132)
Supplement: jkad132_Supplementary_Data [file jkad132_supplementary_data.zip › Table_S2_G3-2023-404168.docx]

**Supplementary Table S2**

| **Supplementary Table 2. Enriched gene ontology categories for suggestive modifiers of Aβ and tau-induced degeneration. Overrepresented gene ontology categories among candidate genes identified across 14 traits. Statistical significance determined by the Benjamini-Hochberg test. GO terms are ranked by Benjamini-Hochberg significance.** | | | |
| --- | --- | --- | --- |
| GO category | GO term | P.adj | Gene ID |
| **Molecular Function (MF)** | |  |  |
| GO:0015370 | solute:sodium symporter activity | 2E-04 | Ndae1/CG13794/CNT1/DAT/CG13795/List/CNT2 |
| GO:0015081 | sodium ion transmembrane transporter activity | 0.002 | nrv2/Ndae1/CG13794/CNT1/DAT/CG13795/ppk18/List/CNT2 |
| GO:0046873 | metal ion transmembrane transporter activity | 0.002 | nrv2/cac/CG5888/Ndae1/SLO2/CG13794/CNT1/DAT/CG13795/ppk18/List/CNT2 |
| GO:0015294 | solute:cation symporter activity | 0.002 | Ndae1/CG13794/CNT1/DAT/CG13795/List/CNT2 |
| GO:0140297 | DNA-binding transcription factor binding | 0.002 | shn/tai/Snr1/lilli/bi/CtBP/retn |
| GO:0015293 | symporter activity | 0.002 | Ndae1/CG13794/CNT1/DAT/CG13795/List/CNT2 |
| GO:0022853 | active ion transmembrane transporter activity | 0.003 | nrv2/Ndae1/Vha44/CG13794/CNT1/DAT/Oatp26F/CG13795/List/CNT2 |
| GO:0061629 | RNA polymerase II-specific DNA-binding transcription factor binding | 0.005 | shn/tai/Snr1/lilli/bi |
| GO:0015077 | monovalent inorganic cation transmembrane transporter activity | 0.005 | nrv2/CG5888/Ndae1/Vha44/SLO2/CG13794/CNT1/DAT/CG13795/ppk18/List/CNT2 |
| GO:0005326 | neurotransmitter transmembrane transporter activity | 0.006 | aralar1/CG13794/DAT/CG13795/List |
| GO:0015291 | secondary active transmembrane transporter activity | 0.009 | Ndae1/CG13794/CNT1/DAT/Oatp26F/CG13795/List/CNT2 |
| GO:0005328 | neurotransmitter:sodium symporter activity | 0.015 | CG13794/DAT/CG13795/List |
| GO:0003700 | DNA-binding transcription factor activity | 0.019 | SREBP/CG17328/shn/lilli/sob/FoxL1/rib/bi/Ets65A/grh/Pdp1/fru/retn/Cf2/Lim1/pros |
| GO:0022890 | inorganic cation transmembrane transporter activity | 0.024 | nrv2/cac/CG5888/Ndae1/Vha44/SLO2/CG13794/CNT1/DAT/CG13795/ppk18/List/CNT2 |
| GO:0008324 | cation transmembrane transporter activity | 0.032 | nrv2/cac/CG5888/Ndae1/Vha44/SLO2/CG13794/CNT1/DAT/CG13795/ppk18/List/CNT2 |
| GO:0000981 | DNA-binding transcription factor activity, RNA polymerase II-specific | 0.038 | SREBP/shn/sob/FoxL1/bi/Ets65A/Pdp1/retn/Cf2/Lim1/pros |
| GO:0022804 | active transmembrane transporter activity | 0.045 | nrv2/Ndae1/Vha44/CG13794/CNT1/DAT/Oatp26F/CG13795/List/CNT2 |
| GO:0003713 | transcription coactivator activity | 0.046 | shn/tai/Snr1/CtBP/mam |
| GO:0005283 | amino acid:sodium symporter activity | 0.046 | CG13794/CG13795/List |
| GO:0005343 | organic acid:sodium symporter activity | 0.046 | CG13794/CG13795/List |
|  |  |  |  |
| **Cellular Components (CC)** | |  |  |
| GO:0045177 | apical part of cell | 5E-04 | dlg1/nuf/cac/scaf/dally/bbg/sano/kibra/RhoGEF64C/fry/sdk/pros |
| GO:0031226 | intrinsic component of plasma membrane | 0.002 | nrv2/dlg1/CadN/tutl/CG5888/Ndae1/AstA-R1/dally/Dscam1/Ac13E/beat-Ic/TyrR/dpr8/sdk/CNT1/DAT/Tsp68C/Oatp26F/Osi23/CNT2 |
| GO:0005887 | integral component of plasma membrane | 0.003 | nrv2/dlg1/CadN/tutl/CG5888/Ndae1/AstA-R1/Dscam1/Ac13E/beat-Ic/TyrR/dpr8/sdk/CNT1/DAT/Tsp68C/Oatp26F/Osi23/CNT2 |
| GO:0005938 | cell cortex | 0.003 | dlg1/nuf/Fife/drongo/sano/igl/kibra/RhoGEF64C/fry/pros/GMF |
| GO:0099738 | cell cortex region | 0.004 | dlg1/nuf/Fife/sano/kibra/RhoGEF64C/pros |
| GO:0098595 | perivitelline space | 0.004 | Sdc/dally/kuz/sdk |
| GO:0045179 | apical cortex | 0.004 | dlg1/nuf/sano/kibra/RhoGEF64C/pros |
| GO:0005911 | cell-cell junction | 0.007 | nrv2/dlg1/CadN/mesh/sns/bbg/FER/kibra |
| GO:0043005 | neuron projection | 0.01 | dpr12/nrv2/dlg1/CadN/tutl/CG44153/plum/Dscam1/CG13794/sNPF/dpr8/fry/DAT/CG13795/CG13506/Ir76a |
| GO:0005918 | septate junction | 0.026 | nrv2/dlg1/mesh/bbg |
| GO:0070161 | anchoring junction | 0.028 | nrv2/dlg1/CadN/mesh/sns/bbg/FER/kibra |
| GO:0030424 | axon | 0.031 | nrv2/dlg1/CadN/tutl/CG44153/plum/Dscam1/sNPF/fry/CG13506 |
| GO:0070160 | tight junction | 0.031 | nrv2/dlg1/mesh/bbg |
| GO:0098590 | plasma membrane region | 0.036 | dpr12/dlg1/nuf/cac/Flo2/CG8176/Fife/scaf/dally/kibra/dpr8/DAT |
|  |  |  |  |
| **Biological Process** |  |  |  |
| GO:0032990 | cell part morphogenesis | 2E-05 | beat-Ia/beat-Vc/shn/CadN/tai/tutl/Snr1/CG44153/Snoo/TrpRS/jbug/Sdc/dally/Dscam1/beat-Ic/kuz/heix/beat-IIIc/fru/retn/exex/FER/RhoGEF64C/fry/pros |
| GO:0048667 | cell morphogenesis involved in neuron differentiation | 2E-05 | beat-Ia/beat-Vc/shn/CadN/tai/tutl/Snr1/CG44153/Snoo/TrpRS/jbug/Sdc/dally/Dscam1/beat-Ic/kuz/beat-IIIc/fru/retn/exex/FER/RhoGEF64C/fry/pros |
| GO:0048812 | neuron projection morphogenesis | 2E-05 | beat-Ia/beat-Vc/shn/CadN/tai/tutl/Snr1/CG44153/Snoo/TrpRS/jbug/Sdc/dally/Dscam1/beat-Ic/kuz/beat-IIIc/fru/retn/exex/FER/RhoGEF64C/fry/pros |
| GO:0120039 | plasma membrane bounded cell projection morphogenesis | 2E-05 | beat-Ia/beat-Vc/shn/CadN/tai/tutl/Snr1/CG44153/Snoo/TrpRS/jbug/Sdc/dally/Dscam1/beat-Ic/kuz/beat-IIIc/fru/retn/exex/FER/RhoGEF64C/fry/pros |
| GO:0048858 | cell projection morphogenesis | 2E-05 | beat-Ia/beat-Vc/shn/CadN/tai/tutl/Snr1/CG44153/Snoo/TrpRS/jbug/Sdc/dally/Dscam1/beat-Ic/kuz/beat-IIIc/fru/retn/exex/FER/RhoGEF64C/fry/pros |
| GO:0007155 | cell adhesion | 2E-05 | beat-Ia/nrv2/dlg1/beat-Vc/CadN/tutl/CG44153/Flo2/mesh/beat-Ic/Loxl2/sns/beat-IIIc/sdk/Invadolysin/CG13506 |
| GO:0098742 | cell-cell adhesion via plasma-membrane adhesion molecules | 2E-05 | beat-Ia/beat-Vc/CadN/tutl/CG44153/beat-Ic/sns/beat-IIIc/sdk/CG13506 |
| GO:0022610 | biological adhesion | 2E-05 | beat-Ia/nrv2/dlg1/beat-Vc/CadN/tutl/CG44153/Flo2/mesh/beat-Ic/Loxl2/sns/beat-IIIc/sdk/Invadolysin/CG13506 |
| GO:0008037 | cell recognition | 2E-05 | beat-Ia/dpr12/CadN/tutl/CG44153/Pvf3/Dscam1/beat-Ic/beat-IIIc/fru/igl/fry |
| GO:0031175 | neuron projection development | 3E-05 | beat-Ia/beat-Vc/shn/CadN/tai/tutl/Snr1/CG44153/Snoo/TrpRS/jbug/Sdc/dally/Dscam1/beat-Ic/kuz/beat-IIIc/fru/retn/exex/FER/RhoGEF64C/fry/pros |
| GO:0097485 | neuron projection guidance | 3E-05 | beat-Ia/beat-Vc/CadN/tutl/Snr1/CG44153/jbug/Sdc/dally/Dscam1/beat-Ic/kuz/beat-IIIc/fru/retn/FER/RhoGEF64C/pros |
| GO:0006935 | chemotaxis | 4E-05 | beat-Ia/beat-Vc/CadN/tutl/Snr1/CG44153/jbug/Sdc/dally/Dscam1/beat-Ic/kuz/beat-IIIc/fru/retn/FER/RhoGEF64C/pros |
| GO:0042330 | taxis | 5E-05 | beat-Ia/nmo/dlg1/beat-Vc/CadN/tutl/Snr1/CG44153/jbug/Sdc/dally/Dscam1/beat-Ic/kuz/beat-IIIc/fru/retn/FER/RhoGEF64C/pros |
| GO:0007411 | axon guidance | 7E-05 | beat-Ia/beat-Vc/CadN/tutl/CG44153/jbug/Sdc/dally/Dscam1/beat-Ic/kuz/beat-IIIc/fru/retn/FER/RhoGEF64C/pros |
| GO:0007409 | axonogenesis | 7E-05 | beat-Ia/beat-Vc/CadN/tai/tutl/CG44153/jbug/Sdc/dally/Dscam1/beat-Ic/kuz/beat-IIIc/fru/retn/exex/FER/RhoGEF64C/pros |
| GO:0008038 | neuron recognition | 7E-05 | beat-Ia/dpr12/CadN/tutl/CG44153/Pvf3/Dscam1/beat-Ic/beat-IIIc/fru/fry |
| GO:0061564 | axon development | 1E-04 | beat-Ia/beat-Vc/CadN/tai/tutl/CG44153/jbug/Sdc/dally/Dscam1/beat-Ic/kuz/beat-IIIc/fru/retn/exex/FER/RhoGEF64C/pros |
| GO:0098609 | cell-cell adhesion | 2E-04 | beat-Ia/dlg1/beat-Vc/CadN/tutl/CG44153/beat-Ic/sns/beat-IIIc/sdk/CG13506 |
| GO:0048813 | dendrite morphogenesis | 4E-04 | shn/CadN/tutl/Snr1/Snoo/TrpRS/dally/Dscam1/fru/retn/fry/pros |
| GO:0045893 | positive regulation of transcription, DNA-templated | 6E-04 | SREBP/CG17328/shn/tai/Snr1/lilli/sob/bi/grh/Pdp1/CtBP/Xpd/retn/Cf2/mam/fry/Lim1/l(2)k09022/Rbfox1/pros |
| GO:1902680 | positive regulation of RNA biosynthetic process | 6E-04 | SREBP/CG17328/shn/tai/Snr1/lilli/sob/bi/grh/Pdp1/CtBP/Xpd/retn/Cf2/mam/fry/Lim1/l(2)k09022/Rbfox1/pros |
| GO:1903508 | positive regulation of nucleic acid-templated transcription | 6E-04 | SREBP/CG17328/shn/tai/Snr1/lilli/sob/bi/grh/Pdp1/CtBP/Xpd/retn/Cf2/mam/fry/Lim1/l(2)k09022/Rbfox1/pros |
| GO:0016358 | dendrite development | 6E-04 | shn/CadN/tutl/Snr1/Snoo/TrpRS/dally/Dscam1/fru/retn/fry/pros |
| GO:0051254 | positive regulation of RNA metabolic process | 0.001 | SREBP/CG17328/shn/tai/Snr1/lilli/sob/bi/grh/Pdp1/CtBP/Xpd/retn/Cf2/mam/fry/Lim1/l(2)k09022/Rbfox1/pros |
| GO:0090066 | regulation of anatomical structure size | 0.001 | nrv2/CadN/lilli/Pvf3/rib/grh/kuz/raw/fru/sano/sNPF/GMF/Syx5/CG6191 |
| GO:0045935 | positive regulation of nucleobase-containing compound metabolic process | 0.001 | SREBP/CG17328/shn/tai/Snr1/lilli/sob/bi/grh/Pdp1/CtBP/Xpd/retn/Cf2/mam/fry/Lim1/l(2)k09022/Rbfox1/pros |
| GO:0048569 | post-embryonic animal organ development | 0.002 | nmo/dlg1/shn/Snr1/sob/scaf/bi/dally/heix/sns/fru/mam/RhoGEF64C/fry/Lim1/Rbfox1/Syx5/CG6191/fy/ics |
| GO:0007157 | heterophilic cell-cell adhesion via plasma membrane cell adhesion molecules | 0.002 | beat-Ia/beat-Vc/beat-Ic/sns/beat-IIIc |
| GO:0022604 | regulation of cell morphogenesis | 0.002 | CadN/tutl/scaf/rib/Dscam1/kuz/raw/FER/fry/pros |
| GO:0007156 | homophilic cell adhesion via plasma membrane adhesion molecules | 0.002 | CadN/tutl/CG44153/sns/sdk/CG13506 |
| GO:0048589 | developmental growth | 0.005 | nmo/dlg1/CadN/tai/cac/scaf/Sdc/bi/plum/dally/Dscam1/kuz/raw/FER/kibra/sNPF |
| GO:0070593 | dendrite self-avoidance | 0.006 | tutl/CG44153/Dscam1/fry |
| GO:0007560 | imaginal disc morphogenesis | 0.007 | nmo/dlg1/shn/Snr1/sob/scaf/bi/dally/fru/mam/RhoGEF64C/fry/Lim1/Rbfox1/CG6191/fy/ics |
| GO:0048563 | post-embryonic animal organ morphogenesis | 0.007 | nmo/dlg1/shn/Snr1/sob/scaf/bi/dally/fru/mam/RhoGEF64C/fry/Lim1/Rbfox1/CG6191/fy/ics |
| GO:0001667 | ameboidal-type cell migration | 0.008 | tai/BicC/sr/rib/Sdc/dally/kuz/Sply/bbg/kibra/GMF |
| GO:0035120 | post-embryonic appendage morphogenesis | 0.008 | nmo/shn/Snr1/sob/bi/dally/fru/mam/RhoGEF64C/fry/Lim1/Rbfox1/CG6191/fy/ics |
| GO:0051960 | regulation of nervous system development | 0.009 | nmo/dlg1/CadN/tutl/cac/Snr1/Sdc/plum/grh/Dscam1/kuz/kibra/fry/sdk/pros |
| GO:0016477 | cell migration | 0.009 | tai/BicC/sr/Pvf3/rib/Sdc/dally/Dscam1/kuz/Sply/bbg/kibra/GMF/Invadolysin |
| GO:0035114 | imaginal disc-derived appendage morphogenesis | 0.01 | nmo/shn/Snr1/sob/bi/dally/fru/mam/RhoGEF64C/fry/Lim1/Rbfox1/CG6191/fy/ics |
| GO:0035107 | appendage morphogenesis | 0.01 | nmo/shn/Snr1/sob/bi/dally/fru/mam/RhoGEF64C/fry/Lim1/Rbfox1/CG6191/fy/ics |
| GO:0010631 | epithelial cell migration | 0.011 | tai/BicC/sr/rib/Sdc/dally/kuz/bbg/kibra/GMF |
| GO:0090132 | epithelium migration | 0.011 | tai/BicC/sr/rib/Sdc/dally/kuz/bbg/kibra/GMF |
| GO:0048737 | imaginal disc-derived appendage development | 0.011 | nmo/shn/Snr1/sob/bi/dally/fru/mam/RhoGEF64C/fry/Lim1/Rbfox1/CG6191/fy/ics |
| GO:0035220 | wing disc development | 0.012 | nmo/nuf/shn/Snr1/lilli/scaf/bi/dally/CtBP/fru/mam/fry/Rbfox1/CG6191/fy/ics |
| GO:0048736 | appendage development | 0.012 | nmo/shn/Snr1/sob/bi/dally/fru/mam/RhoGEF64C/fry/Lim1/Rbfox1/CG6191/fy/ics |
| GO:0051093 | negative regulation of developmental process | 0.012 | dlg1/shn/CadN/Snr1/Snoo/plum/heix/kibra/sdk/pros/lark |
| GO:0090130 | tissue migration | 0.014 | tai/BicC/sr/rib/Sdc/dally/kuz/bbg/kibra/GMF |
| GO:0007394 | dorsal closure, elongation of leading edge cells | 0.014 | scaf/raw/FER |
| GO:0048592 | eye morphogenesis | 0.014 | beat-Ia/nmo/shn/CadN/tutl/lilli/bi/grh/sns/sano/kibra/fry/sdk/pros |
| GO:0090596 | sensory organ morphogenesis | 0.014 | beat-Ia/nmo/shn/CadN/tutl/lilli/bi/grh/sns/sano/kibra/fry/sdk/pros |
| GO:0001654 | eye development | 0.014 | beat-Ia/nmo/shn/CadN/tutl/lilli/bi/dally/grh/sns/sano/kibra/fry/Lim1/sdk/pros |
| GO:0048880 | sensory system development | 0.014 | beat-Ia/nmo/shn/CadN/tutl/lilli/bi/dally/grh/sns/sano/kibra/fry/Lim1/sdk/pros |
| GO:0150063 | visual system development | 0.014 | beat-Ia/nmo/shn/CadN/tutl/lilli/bi/dally/grh/sns/sano/kibra/fry/Lim1/sdk/pros |
| GO:0048707 | instar larval or pupal morphogenesis | 0.015 | nmo/dlg1/shn/Snr1/sob/scaf/bi/dally/fru/mam/RhoGEF64C/fry/Lim1/Rbfox1/CG6191/fy/ics |
| GO:0010558 | negative regulation of macromolecule biosynthetic process | 0.015 | dlg1/SREBP/CG17328/shn/BicC/bru2/sob/bi/CtBP/CG6254/retn/Hers/Lim1/Rbfox1/pros/Rcd-1r/timeout |
| GO:2000113 | negative regulation of cellular macromolecule biosynthetic process | 0.015 | dlg1/SREBP/CG17328/shn/BicC/bru2/sob/bi/CtBP/CG6254/retn/Hers/Lim1/Rbfox1/pros/Rcd-1r/timeout |
| GO:0007414 | axonal defasciculation | 0.015 | beat-Ia/tutl/beat-Ic |
| GO:0008045 | motor neuron axon guidance | 0.016 | beat-Ia/beat-Vc/Sdc/dally/beat-Ic/beat-IIIc |
| GO:0048749 | compound eye development | 0.017 | nmo/shn/CadN/tutl/lilli/bi/dally/grh/sns/sano/kibra/fry/Lim1/sdk/pros |
| GO:0007472 | wing disc morphogenesis | 0.017 | nmo/shn/Snr1/scaf/bi/dally/fru/mam/fry/Rbfox1/CG6191/fy/ics |
| GO:0040007 | growth | 0.017 | nmo/dlg1/CadN/tai/cac/scaf/Sdc/bi/plum/dally/Dscam1/kuz/raw/FER/kibra/sNPF |
| GO:0009890 | negative regulation of biosynthetic process | 0.017 | dlg1/SREBP/CG17328/shn/BicC/bru2/sob/bi/CtBP/CG6254/retn/Hers/Lim1/Rbfox1/pros/Rcd-1r/timeout |
| GO:0031327 | negative regulation of cellular biosynthetic process | 0.017 | dlg1/SREBP/CG17328/shn/BicC/bru2/sob/bi/CtBP/CG6254/retn/Hers/Lim1/Rbfox1/pros/Rcd-1r/timeout |
| GO:0009886 | post-embryonic animal morphogenesis | 0.017 | nmo/dlg1/shn/Snr1/sob/scaf/bi/dally/fru/mam/RhoGEF64C/fry/Lim1/Rbfox1/CG6191/fy/ics |
| GO:0001745 | compound eye morphogenesis | 0.019 | nmo/shn/CadN/tutl/lilli/bi/grh/sns/sano/kibra/fry/sdk/pros |
| GO:0060560 | developmental growth involved in morphogenesis | 0.019 | CadN/tai/scaf/Dscam1/kuz/raw/FER |
| GO:0051241 | negative regulation of multicellular organismal process | 0.021 | dlg1/shn/CadN/Snr1/plum/heix/kibra/sdk/pros/lark |
| GO:0035150 | regulation of tube size | 0.021 | nrv2/rib/grh/raw/sano |
| GO:0048190 | wing disc dorsal/ventral pattern formation | 0.021 | nmo/nuf/shn/lilli/dally |
| GO:0006836 | neurotransmitter transport | 0.022 | cac/Fife/aralar1/CG13794/DAT/Syx5/CG13795/List |
| GO:0022603 | regulation of anatomical structure morphogenesis | 0.022 | shn/CadN/tutl/scaf/rib/Dscam1/kuz/raw/FER/fry/pros |
| GO:0035222 | wing disc pattern formation | 0.023 | nmo/nuf/shn/lilli/bi/dally |
| GO:0007391 | dorsal closure | 0.023 | dlg1/shn/Ack-like/scaf/rib/raw/FER |
| GO:0009792 | embryo development ending in birth or egg hatching | 0.023 | dlg1/shn/sr/Ack-like/scaf/rib/sns/raw/FER/lark |
| GO:0035296 | regulation of tube diameter | 0.023 | nrv2/rib/raw |
| GO:0048870 | cell motility | 0.023 | tai/BicC/sr/Pvf3/rib/Sdc/dally/Dscam1/kuz/Sply/bbg/kibra/GMF/Invadolysin |
| GO:0048814 | regulation of dendrite morphogenesis | 0.024 | CadN/tutl/Dscam1/fry |
| GO:0007447 | imaginal disc pattern formation | 0.024 | nmo/nuf/shn/lilli/bi/dally/Lim1 |
| GO:0007417 | central nervous system development | 0.024 | nrv2/sr/rib/grh/Dscam1/kuz/raw/fru/exex/mam/pros/Invadolysin |
| GO:0042063 | gliogenesis | 0.025 | nrv2/Sdc/kuz/retn/kibra/pros |
| GO:0007450 | dorsal/ventral pattern formation, imaginal disc | 0.025 | nmo/nuf/shn/lilli/dally |
| GO:0051674 | localization of cell | 0.025 | tai/BicC/sr/Pvf3/rib/Sdc/dally/Dscam1/kuz/Sply/bbg/kibra/GMF/Invadolysin |
| GO:0045944 | positive regulation of transcription by RNA polymerase II | 0.025 | SREBP/CG17328/shn/tai/lilli/sob/bi/grh/CtBP/Cf2/mam/Lim1/pros |
| GO:0007392 | initiation of dorsal closure | 0.025 | scaf/raw/FER |
| GO:0034329 | cell junction assembly | 0.025 | nrv2/nmo/dlg1/cac/mesh/Fife/Sdc/plum/sns/pros |
| GO:0045887 | positive regulation of synaptic growth at neuromuscular junction | 0.027 | nmo/dlg1/cac/Sdc |
| GO:0030855 | epithelial cell differentiation | 0.027 | dlg1/tai/BicC/cni/grh/kuz/lama/bbg/exex/Cf2/kibra/mam/fry/GMF/fy |
| GO:0007476 | imaginal disc-derived wing morphogenesis | 0.028 | nmo/shn/Snr1/bi/dally/fru/mam/fry/Rbfox1/CG6191/fy/ics |
| GO:0001738 | morphogenesis of a polarized epithelium | 0.028 | nmo/dlg1/CadN/jbug/grh/sano/fy |
| GO:0048100 | wing disc anterior/posterior pattern formation | 0.029 | shn/bi/dally |
| GO:0048638 | regulation of developmental growth | 0.03 | nmo/dlg1/CadN/cac/Sdc/plum/dally/kuz/kibra/sNPF |
| GO:1904398 | positive regulation of neuromuscular junction development | 0.031 | nmo/dlg1/cac/Sdc |
| GO:0010769 | regulation of cell morphogenesis involved in differentiation | 0.034 | CadN/tutl/Dscam1/kuz/fry/pros |
| GO:0051965 | positive regulation of synapse assembly | 0.036 | nmo/dlg1/cac/Sdc |
| GO:1901890 | positive regulation of cell junction assembly | 0.036 | nmo/dlg1/cac/Sdc |
| GO:0001700 | embryonic development via the syncytial blastoderm | 0.038 | dlg1/shn/sr/Ack-like/scaf/rib/sns/raw/FER |
| GO:0034330 | cell junction organization | 0.038 | dpr12/nrv2/nmo/dlg1/cac/mesh/Fife/Sdc/plum/sns/dpr8/pros/CG13506 |
| GO:0016331 | morphogenesis of embryonic epithelium | 0.039 | dlg1/shn/Ack-like/scaf/rib/raw/FER |
